# Quantification of putative ovarian cancer serum protein biomarkers using a multiplexed targeted mass spectrometry assay

**DOI:** 10.1186/s12014-023-09447-4

**Published:** 2024-01-03

**Authors:** Joohyun Ryu, Kristin L. M. Boylan, Carly A. I. Twigg, Richard Evans, Amy P. N. Skubitz, Stefani N. Thomas

**Affiliations:** 1 Department of Laboratory Medicine and Pathology, University of Minnesota School of Medicine, Minneapolis, MN USA; 2 Clinical and Translational Research Institute, University of Minnesota, Minneapolis, MN USA

**Keywords:** Targeted mass spectrometry, Parallel reaction monitoring (PRM), Diagnostic biomarkers, High-grade serous ovarian cancer (HGSOC), Serum, Insulin-like growth factor-binding protein 2 (IBP2)

## Abstract

**Background:**

Ovarian cancer is the most lethal gynecologic malignancy in women, and high-grade serous ovarian cancer (HGSOC) is the most common subtype. Currently, no clinical test has been approved by the FDA to screen the general population for ovarian cancer. This underscores the critical need for the development of a robust methodology combined with novel technology to detect diagnostic biomarkers for HGSOC in the sera of women. Targeted mass spectrometry (MS) can be used to identify and quantify specific peptides/proteins in complex biological samples with high accuracy, sensitivity, and reproducibility. In this study, we sought to develop and conduct analytical validation of a multiplexed Tier 2 targeted MS parallel reaction monitoring (PRM) assay for the relative quantification of 23 putative ovarian cancer protein biomarkers in sera.

**Methods:**

To develop a PRM method for our target peptides in sera, we followed nationally recognized consensus guidelines for validating fit-for-purpose Tier 2 targeted MS assays. The endogenous target peptide concentrations were calculated using the calibration curves in serum for each target peptide. Receiver operating characteristic (ROC) curves were analyzed to evaluate the diagnostic performance of the biomarker candidates.

**Results:**

We describe an effort to develop and analytically validate a multiplexed Tier 2 targeted PRM MS assay to quantify candidate ovarian cancer protein biomarkers in sera. Among the 64 peptides corresponding to 23 proteins in our PRM assay, 24 peptides corresponding to 16 proteins passed the assay validation acceptability criteria. A total of 6 of these peptides from insulin-like growth factor-binding protein 2 (IBP2), sex hormone-binding globulin (SHBG), and TIMP metalloproteinase inhibitor 1 (TIMP1) were quantified in sera from a cohort of 69 patients with early-stage HGSOC, late-stage HGSOC, benign ovarian conditions, and healthy (non-cancer) controls. Confirming the results from previously published studies using orthogonal analytical approaches, IBP2 was identified as a diagnostic biomarker candidate based on its significantly increased abundance in the late-stage HGSOC patient sera compared to the healthy controls and patients with benign ovarian conditions.

**Conclusions:**

A multiplexed targeted PRM MS assay was applied to detect candidate diagnostic biomarkers in HGSOC sera. To evaluate the clinical utility of the IBP2 PRM assay for HGSOC detection, further studies need to be performed using a larger patient cohort.

**Supplementary Information:**

The online version contains supplementary material available at 10.1186/s12014-023-09447-4.

## Background

Ovarian cancer is the sixth most common cause of cancer death in women [1]. Although the incidence of ovarian cancer is lower than breast cancer, the mortality rate for ovarian cancer is 2.25 times higher than for breast cancer, making it the most lethal gynecologic malignancy in women [1]. High-grade serous ovarian cancer (HGSOC) is the most common histological subtype, accounting for 70% of all types of ovarian cancer [2, 3]. A major challenge in treating HGSOC is that more than 70% of all diagnoses are made when the disease has established regional or distant metastases [4]. This is because the symptoms do not appear until the disease is in an advanced stage [5]. Long-term outcomes of HGSOC treatment have not changed significantly in the past 30 years: the 5-year overall survival is 20–40% for stage III and IV disease. In contrast, patients with stage I disease have a > 90% 5-year overall survival [4, 5]. Therefore, early detection is critical to increase the survival rates of patients with HGSOC [6].

Current clinical HGSOC diagnostic tests rely on measuring serum CA125 (MUC16) levels to test women who have vague symptoms of HGSOC, or to monitor women who have been diagnosed with HGSOC or who are at high risk of developing HGSOC. However, this method is not adequately sensitive or specific to screen for HGSOC in the general population [7–9]. Thus, routine screening such as imaging (transvaginal ultrasound, computed tomography scan, and magnetic resonance imaging) and pelvic exams are recommended for women who are at higher risk, such as those with a family history of the disease or those who carry mutations in the *BRCA1/2* genes. The current lack of a reliable screening test for HGSOC in the general population emphasizes the critical need for more robust diagnostic biomarkers.

Targeted mass spectrometry (MS) methods such as parallel reaction monitoring (PRM) and multiple reaction monitoring (MRM) can identify and quantify specific peptides/proteins with high accuracy, sensitivity, and reproducibility in complex biological samples [10–13]. A promising clinical application of targeted MS is the identification and quantification of peptide or protein cancer biomarkers [14–21]. Recent research has identified serum glycoprotein HGSOC biomarkers using MRM [22].

PRM is a targeted MS method performed using a mass spectrometer with a high-resolution accurate mass (HRAM) analyzer such as the Orbitrap allowing the parallel detection of all product ions from a targeted precursor ion, which is in contrast to MRM wherein typically only 2–3 product ions are monitored [23, 24]. PRM reduces the time for method development by eliminating the need to predetermine product ions and collision energies [25]. In addition, PRM methods generally yield high signal-to-noise ratios due to the high sensitivity of HRAM-MS, indicating that HRAM-MS can minimize interference from co-isolated background ions [26–28]. Therefore, PRM has become a valuable technique in cancer research, enabling the development of new diagnostic approaches [29].

In this study, we sought to develop and validate a multiplexed, fit-for-purpose Tier 2 targeted PRM MS assay for the relative quantification of HGSOC protein biomarker candidates in sera according to nationally recognized consensus guidelines [30, 31]. Subsequently, we identified a set of proteins with differential levels in the sera of patients with HGSOC compared to sera from healthy women or women with benign ovarian conditions, suggesting that these proteins may play an important role in the pathogenesis of HGSOC and could be developed as diagnostic biomarkers. Of note, for our PRM assay, the measurands – defined by the International Vocabulary of Metrology as the quantity intended to be measured – were tryptic peptides from these candidate diagnostic protein biomarkers.

Disparities in ovarian cancer disease presentation, diagnosis, treatment and survival exist between African American women and non-Hispanic white women with equivalent access to health care [32–36]. A potential reason for these disparities is the historical and continual focus of research on Caucasian subjects. The PRM assays presented in our studies were developed using a predominately Caucasian cohort. The continued development of these assays will enable the inclusion of a more diverse group of participants, ensuring the widest applicability of the resulting diagnostic tools for ovarian cancer.

## Methods

### Serum samples

Blood was obtained by the University of Minnesota Tissue Procurement Facility staff with approval by the University of Minnesota Institutional Review Board under Protocol 0407M62504. After signing the consent form, blood was collected immediately before surgery from women with an abdominal mass suspected to be ovarian cancer (for the benign ovarian disease, early-stage HGSOC, and late-stage HGSOC cases) or from women with benign non-gynecological health conditions (e.g., eye surgery, hernia repair, hip replacement, and gallbladder removal) to serve as non-cancer controls. All women fasted overnight prior to their surgery the following morning and their blood collection. Blood was processed into serum by standard operating procedures [37], divided into aliquots, and stored at − 80 °C. Serum samples were selected from each of four groups of patients: (i) non-cancer controls (n = 18), (ii) benign ovarian disease (n = 18), (iii) early-stage I/II HGSOC (n = 16), and (iv) late-stage III/IV HGSOC (n = 17). All the participants in the “benign ovarian disease”, “early-stage HGSOC”, and “late-stage HGSOC” groups were Caucasian, and the median age was 60 years (range: 36 – 83 years). Ethnic membership of the “non-cancerous” group was incompletely documented, and of the 18 participants in this group, at least 5 were Caucasian and 1 was African American (Table 1). CA125 values were abstracted from patients’ medical records for ovarian cancer patients and women with benign conditions or determined by a commercially available ELISA for the non-cancer controls (Table 1) [38]. At the time of patient serum specimen collection (2005 – 2013), incomplete demographic data regarding race and ethnicity was captured, hence the 12 patients in this cohort for whom their race is documented as “unidentified.”

**Table 1:** Clinical information of patients

Category	Non-cancerous	Benign ovarian condition	Early stage serous ovarian cancer	Late stage serous ovarian cancer	Total
n	18	18	16	17	69
Age range (median)	50–83 (62.5)	36–81 (49)	42–83 (61)	53–75 (65)	36–83 (60)
Race (%)					
Caucasian	5	17	13	17	52 (75.4%)
African American	1				5 (7.2%)
Unidentified	12				12 (17.4%)
Stage I			10		
Stage II			6		
Stage III				15	
Stage IV				2	
CA125 range (median)*	0–69 (1.55)	6–413 (14.5)	32–22780 (108)	124–3417 (584)	

^*^CA125 levels were measured as U/mL

### Ovarian cancer biomarkers

A total of 23 proteins were selected from our previous studies and the literature as potential ovarian cancer serum biomarker candidates (Table 2).

**Table 2:** The 23 proteins and their corresponding light- and heavy-isotope labeled peptides used for the PRM assay

UniProt accession number	Protein name	Synthetic Light (Lys0 or Arg0) and Heavy (Lys8 or Arg10) peptides	References
sp|Q8WXI7|MUC16_HUMAN	Mucin-16 (CA125)	LTNDIEELGPYTLDR*, VAIYEEFLR*, VISPVVTSSSIR*, STISSLGTPSISTK*, ELGPYTLDR*	[70, 71]
sp|P22223|CADH3_HUMAN	Cadherin-3	FTQDTFR*, EPLLLPEDDTR*, ETGWLLLNKPLDR*, STGTISVISSGLDR*, LTVTDLDAPNSPAWR*	[72]
sp|P02771|FETA_HUMAN	Alpha-fetoprotein	DFNQFSSGEK*, NIFLASFVHEYSR*	[73, 74]
sp|P09038|FGF2_HUMAN	Fibroblast growth factor 2	LESNNYNTYR*, YTSWYVALK*	[75]
sp|P15328|FOLR1_HUMAN	Folate receptor alpha	DVSYLYR*, GWNWTSGFNK*, VLNVPLCK*	[76]
sp|P19883|FST_HUMAN	Follistatin	LSTSWTEEDVNDNTLFK*, SIGLAYEGK*	[77]
sp|Q99988|GDF15_HUMAN	Growth/differentiation factor 15	YEDLLTR*, AALPEGLPEASR*	[78, 79]
sp|P08833|IBP1_HUMAN	Insulin-like growth factor-binding protein 1	IPGSPEIR*, ALPGEQQPLHALTR*	[80]
sp|P18065|IBP2_HUMAN	Insulin-like growth factor-binding protein 2	LIQGAPTIR*, LEGEACGVYTPR*	[81]
sp|P05231|IL6_HUMAN	Interleukin-6	VLIQFLQK*, NLDAITTPDPTTNASLLTK*, YILDGISALR*, EALAENNLNLPK*	[78, 82]
sp|Q92876|KLK6_HUMAN	Kallikrein-6	LSELIQPLPLER*, PGVYTNVCR*	[78, 83, 84]
sp|Q9UBX7|KLK11_HUMAN	Kallikrein-11	CANITIIEHQK*, LLCGATLIAPR*	[78, 85]
sp|P08727|K1C19_HUMAN	Keratin, type I cytoskeletal 19	AALEDTLAETEAR*, ALEAANGELEVK*, DAEAWFTSR*, ILGATIENSR*, DYSHYYTTIQDLR*	[86]
sp|Q13421|MSLN_HUMAN	Mesothelin	IQSFLGGAPTEDLK*, GLLPVLGQPIIR*, ANVDLLPR*, TDAVLPLTVAEVQK*	[87]
sp|P15941|MUC1_HUMAN	Mucin-1	NYGQLDIFPAR*	[88, 89]
sp|Q96NY8|NECT4_HUMAN	Nectin-4	NPLDGSVLLR*, LDGPLPSGVR*, GDSGEQVGQVAWAR*, VSTFPAGSFQAR*	[90]
sp|P10451|OSTP_HUMAN	Osteopontin	GDSVVYGLR*, AIPVAQDLNAPSDWDSR*, YPDAVATWLNPDPSQK*	[91]
sp|P01236|PRL_HUMAN	Prolactin	LLEGMELIVSQVHPETK*, LSAYYNLLHCLR*	[92]
sp|Q16651|PRSS8_HUMAN	Prostasin	LGAHQLDSYSEDAK*, PLQQLEVPLISR*	[78, 93]
sp|P04278|SHBG_HUMAN	Sex hormone-binding globulin	IALGGLLFPASNLR*, QAEISASAPTSLR*, LDVDQALNR*	[68]
sp|P01033|TIMP1_HUMAN	TIMP Metalloproteinase inhibitor 1	GFQALGDAADIR*, SEEFLIAGK*, EPGLCTWQSLR*	[94]
sp|P25445|TNR6_HUMAN	Tumor necrosis factor receptor superfamily member 6	DITSDSENSNFR*	[95, 96]
sp|P35968|VGFR2_HUMAN	Vascular endothelial growth factor receptor 2	TFEDIPLEEPEVK*, DYVGAIPVDLK*, FLSTLTIDGVTR*	[78, 83, 97, 98]

^*^Chemically synthesized ^13^C/^15^N-labeled peptides are used as internal standards; labeled amino acids are indicated in bold, and these peptides are labeled on the C-terminal Lys as K8 (^13^C_6_, ^15^N_2_) or C-terminal Arg as R10 (^13^C_6_, ^15^N_4_)

### Target peptide synthesis

Stable isotope-labeled (heavy) peptides labeled on the C-terminal lysine (Lys) as K8 (^13^C_6_,^15^N_2_) or C-terminal arginine (Arg) as R10 (^13^C_6_,^15^N_4_) and the corresponding unlabeled (light) peptides were chemically synthesized by Vivitide/Biosynth (Gardner, MA). A total of 128 crude peptides including 64 light and heavy peptide pairs were synthesized, and the chemical purity was estimated to be > 50%. The isotopic purity of each heavy amino acid was > 99%. MS1 analysis was performed to confirm the monoisotopic mass and the most abundant precursor charge states for each of the 64 light and 64 heavy peptides.

### Sample preparation

The total protein concentration of serum from each patient was determined by a BCA assay (Thermo Fisher Scientific, Waltham, MA). Serum protein (100 µg) was used for trypsin digestion. The serum proteins were denatured using 0.1% RapiGest (Waters, Milford, MA) and 50 mM ammonium bicarbonate, reduced with 5 mM dithiothreitol for 30 min at 60 °C, and then alkylated with 15 mM iodoacetamide for 30 min at room temperature in the dark. Subsequently, trypsin (Promega, Madison, WI) was added at an enzyme/protein ratio of 1:50, followed by incubation for 18 h at 37 °C. The digestion was terminated by adding trifluoroacetic acid (Sigma, St. Louis, MO) to a final concentration of 0.5% (v/v) followed by incubation for 30 min at 37 °C. RapiGest was removed by centrifugation at 13,000 rpm for 10 min. After RapiGest removal, the digested serum was desalted and concentrated using C18 Sep-Pak cartridges containing 1 mg sorbent (Waters) according to the manufacturer’s instructions. The eluted peptides were frozen for 30 min at − 80 °C and then dried by vacuum centrifugation. The peptides were resuspended in 5% acetonitrile and 0.1% formic acid, and the peptide concentration was measured using a NanoDrop (Thermo Fisher Scientific).

### Targeted mass spectrometry parallel reaction monitoring (PRM) assay development and validation

A multiplexed targeted PRM MS assay was developed using a Q Exactive Plus Hybrid Quadrupole-Orbitrap mass spectrometer coupled with a Vanquish HPLC system interfaced with an Ion Max HESI-II source (Thermo Fisher Scientific). Mobile phase A and B contained 0.1% formic acid in water and 90% acetonitrile/ 0.1% formic acid, respectively. The peptides were separated on an Accucore RP-MS 100 mm × 2.1 mm, 2.6 µm column (Thermo Fisher Scientific) with a linear gradient of 5–28% mobile phase B for 30 min at a flow rate of 0.5 mL/min. For measurement of the target peptides, the mass spectrometer was operated in PRM mode. Mass spectra were acquired in profile mode with a setting of 35,000 resolution at 200 m/z, 2e^5^ AGC target, 100 ms maximum injection time (IT), 1.6 m/z isolation window, and normalized collision energy of 30. A scheduled method was used with 1-min retention time windows (concurrently monitored precursors within each window: 2 to 18). Ionization source parameters were as follows: spray voltage, 3.8 kV; sheath gas, 26; auxiliary gas, 6; capillary temperature, 380 °C.

The PRM assay was characterized according to the National Cancer Institute’s Clinical Proteomic Tumor Analysis Consortium (CPTAC) consensus guidelines **(**Table 3**)** [30, 31]. Calibration curves were generated in triplicate with 11 standard points ranging from 0.375 to 4800 fmol (0.375, 0.75, 1.5, 3, 6, 12, 24, 48, 96,480, and 4800 fmol) to determine the limit of detection (LOD), limit of quantification (LOQ), and linearity. The standard points were prepared by dilution of the light peptides in serum (1 µg peptide from digested background serum matrix on column with 10 µL injection volume) spiked with a constant amount of heavy internal standard peptides (200 fmol on column with 10 µL injection volume). The LODs were determined from the blanks using the average plus 3 times the standard deviation of the blank signal with accuracy of 80%-120% at each standard point. Once the LODs were determined, the LOQs were manually calculated using the following equation: LOQ = 3 × LOD with a coefficient of variation (CV) < 20% [39].

**Table 3:** Development and validation of a PRM assay to detect ovarian cancer protein biomarkers in serum

Experiments for method characterization	Digested matrix	Standard	Internal standard	Experimental design
Response curve	Sera (1 µg on LC column)	Mixture of 64-light peptides; 0.375 to 4800 fmol	Mixture of 64-heavy peptides; 200 fmol/peptide	Each light + heavy peptide mix was spiked into digested matrix, followed by LC-PRM analysis (n = 3) on the same day. Top 3 transition ions were selected for quantification unless otherwise indicated. LOD was determined using the average plus 3 × standard deviation of the blank signal. LOQ = 3 × LOD
Validation of repeatability	Sera (1 µg on LC column)	Mixture of 64-light peptides: 2x (low), 50x (medium), and 200x (high) LOQ	Mixture of 64-heavy peptides; 200 fmol/peptide	Each sample of 3 concentrations (low, medium, and high) was analyzed in triplicate on 5 different days
Assessment of selectivity	6 biological replicates of sera (1 µg on LC column)	Mixture of 64-light peptides: no spike (blank), 25 × LOQ, and 50 × LOQ	Mixture of 64-heavy peptides; 200 fmol/peptide	Six biological replicates of undepleted sera with spiked-in light and heavy peptides were analyzed in duplicate in the same experimental block
Quantification of endogenous peptides	69 patient sera specimens (20 µg on column)		Mixture of 64-heavy peptides; 200 fmol/peptide	Endogenous analytes with spiked-in heavy peptides were analyzed in duplicate

To assess the repeatability of the assay, digested background serum matrix was spiked with low (2 × LOQ), medium (50 × LOQ), and high (200 × LOQ) amounts of light peptides and a constant amount of heavy internal standard peptides. The order of the samples was randomized in the acquisition queue. The samples were prepared prior to analysis, injected into an LC column in triplicate, and analyzed on 5 different days. The intra-assay variability expressed as the CV at each level (low, medium, high) was calculated by analyzing the triplicate runs from each day for 5 days. The average intra-assay variability was determined by averaging the intra-assay variability over 5 days. The inter-assay variability was calculated by analyzing the variability of each individual injection of each level over 5 days, then calculating the average. The total assay variability was calculated as the square root of the sum of the squares of the average intra-assay and average inter-assay variability. The assay acceptability criteria was defined as total CV < 20%.

To assess whether the assay is specific for the target peptides, the responses of the target peptides were determined in 6 different serum samples representing 6 biological replicates. Each of the 6 different biological serum replicates was spiked with buffer-only (no spike of analyte), medium (50 × LOQ), and half-medium (25 × LOQ) amount of light peptides and a constant amount of heavy internal standard peptides. The samples were prepared and analyzed by PRM in duplicate. The peak area ratio of light to heavy peptides at each level for each biological replicate was averaged across the duplicate runs and was plotted on the linear scale to determine if the slope of the line for each peptide was within 10% of the mean. The peak area ratio of light to heavy peptides for each biological replicate was also averaged across the duplicate runs to determine if the difference between the observed vs. predicted half-medium amount was < 10%. The peak area intensity between each transition ion for the light peptides was averaged from the duplicate runs to determine whether the ion transition ratio of each peptide was within 30% of the mean.

### Data analysis

The .raw files were imported into Skyline (64-bit) 22.2.0.351. All peak integrations were manually reviewed, and any transitions with detected interferences were omitted from the data analysis. Transition ions were manually selected based on their signal intensities. For the ‘DVSYLYR’ peptide corresponding to FOLR1, only 2 transition ions for the light and heavy peptides were selected. For the other peptides, the 3 transition ions for the light and heavy peptides were manually selected in same manner. The sum of these transition ions represented the integrated peak areas that were used for relative quantification.

### Quantification of target peptides and proteins

The heavy peptides as internal standards were spiked into the 69 digested patient serum samples. Each digested patient serum sample (20 µg peptide) containing 200 fmol of spiked heavy peptides (10 µL injection volume) was injected and then analyzed by our PRM assay in duplicate (n = 2 injections per sample). The concentrations of endogenous target peptides (peak area ratio of each light-to-heavy peptide) were calculated using the calibration curves for each target peptide. Only endogenous target peptides that were quantified at levels exceeding their respective LOQs were used for statistical analysis. To calculate the protein concentration, first the molecular weight of each target protein was determined using its UniProt accession number. The target protein concentrations were then calculated considering the measured concentration of the endogenous target peptides and the molecular weight of corresponding target proteins using the following formula: Protein concentration (g/L) = peptide concentration (mol/L) × protein molecular weight (g/mol).

### Statistical analysis

The statistical analysis was conducted in several steps. First, the data was inspected for potential outliers using summary statistics and checked for veracity. Missing data were omitted on a case-wise basis. Quantitative data are presented as the mean ± standard deviation (s.d.) of the replicate injections (n = 2) of each sample. Group [early-stage HGSOC, late-stage HGSOC, benign ovarian conditions, and healthy (non-cancer) controls] differences were evaluated for each biomarker, first using non-parametric ANOVA and then pairwise non-parametric Holm-adjusted post-hoc tests. The Holm method used a family-wise error rate of 0.05 for statistical significance. The diagnostic characteristics of biomarkers and combinations of biomarkers were assessed with accuracy, sensitivity, specificity, and receiver operating characteristic (ROC) curves. To determine the diagnostic characteristics, the biomarkers were used to predict disease status using logistic regressions. Each logistic regression was fit with the independent variables being ≥ 1 biomarker, and the dependent variable being 2 cancer stages (e.g., late-stage and non-cancer). The logistic regression was used to determine the disease classification probabilities for the 2 cancer stages from the subset of biomarkers. The biomarkers were normalized to ensure that the concentrations of all peptides and proteins were on the same scale for the logistic regression. Using 0.5 as a cutoff, cases with probabilities > 0.5 were predicted to be one of the cancer states (e.g., late-stage), and cases with propensity score < 0.5 were predicted to be the other cancer stage (e.g., non-cancer). Those binary predictions along with the true disease classification were used to calculate accuracy, sensitivity, and specificity (and their 95% Delong confidence intervals (CI)) [35]. For a cutoff-free measure of discrimination, the logistic regression probabilities were used to generate ROC curves and calculate areas under the curves (AUC). The *p*-values for the AUC were the whole model tests *p*-value of the logistic regressions. R version 1.2.2 (2022–10-31 UCRT) was used for statistical analysis (https://r-project.org, https://cran.r-project.org/package=caret). A Quarto document containing the scripts used for the analysis is provided as Additional file 1: File S1.

## Results

### Peptide selection from target proteins

Serum protein ovarian cancer biomarker candidates (n=23) were selected based on our previous studies and other publications (Table 2). Candidate peptides for these 23 proteins were prioritized according to their observability by MS from open-source proteomic databases such as Global Proteome Machine (GPM) (https://thegpm.org) and PeptideAtlas (https://peptideatlas.org). Peptides were selected based on the following criteria: (1) Reactive residues: no readily reactive amino acid residues (His, Trp, Asn/Gln followed by Gly) or missed trypsin cleavage-containing peptides; (2) Uniqueness (analyte specificity): unique peptide sequence as determined by BLAST search; (3) Hydropathy: peptide hydropathy score between 10 and 45; and (4) Peptide length: 7–20 amino acids. A total of 1 – 5 peptides were selected for each protein target. A total of 64 peptides were selected for the PRM assay (Table 2).

### Development and validation of a PRM assay

Normal human serum was selected as the background matrix within which to establish the analytical performance of our assay. With a future goal of developing a sample preparation workflow that is in congruence with the streamlined sample preparation workflows in most clinical laboratories, we avoided the inclusion of an immunoaffinity-based protein depletion step for the preparation of the serum matrix. Additionally, the use of non-depleted serum as the background matrix avoids the analytical variability introduced by the inclusion of an additional sample preparation step.

**Fig. 1:**
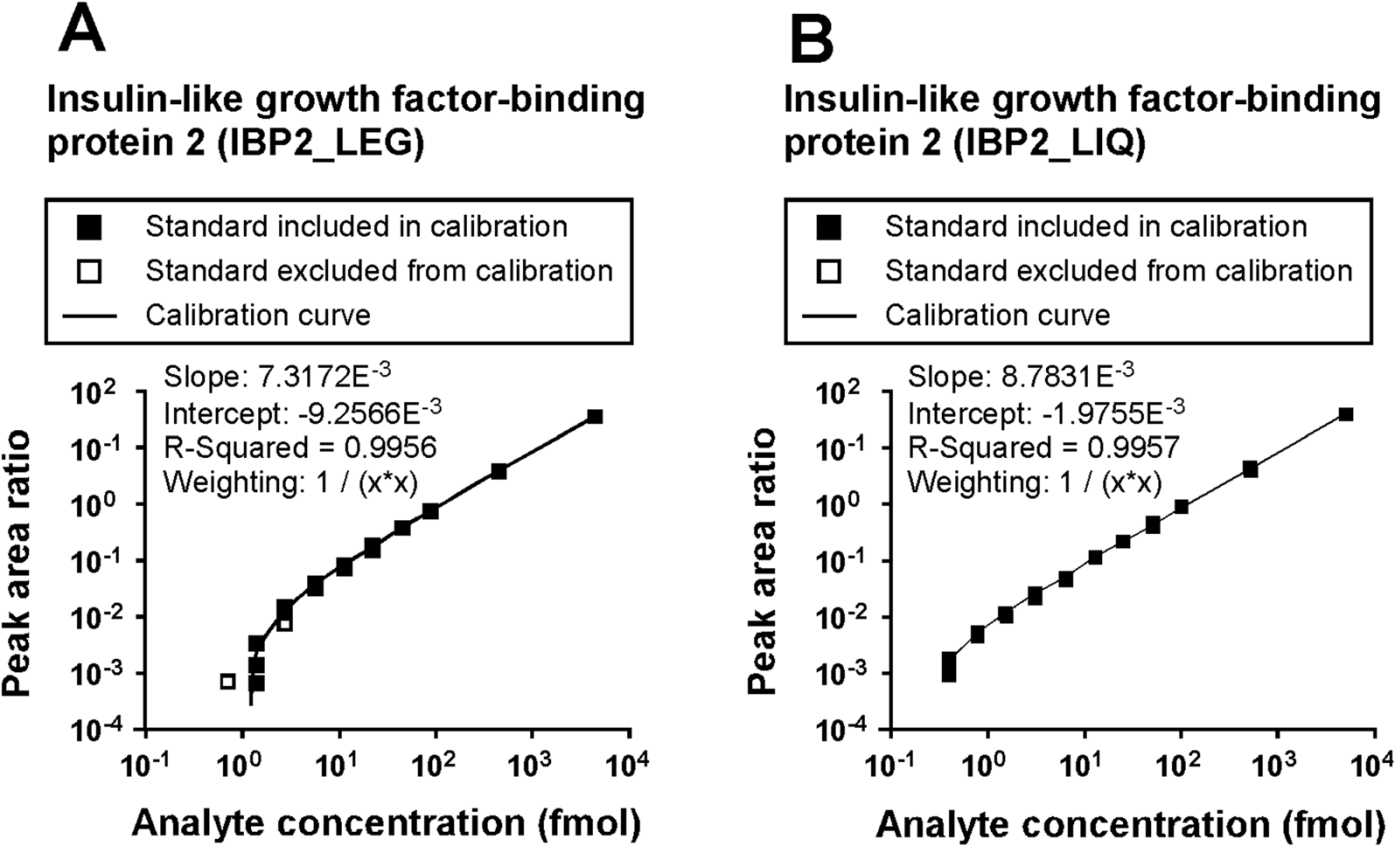
Representative calibration curve data. **A**, **B** Calibration curves for ‘LEGEACGVYTPR’ (IBP2_LEG) and ‘LIQGAPTIR’ (IBP2_LIQ) peptides derived from insulin-like growth factor-binding protein 2 (IBP2). Standard points were excluded from calibration curve if the accuracy was > 20%. Log_10_ value was used for the x-axis and the y-axis

The LOQs for the 64 light peptides were determined based on the calibration curves that spanned 4 orders of magnitude ranging from 0.375 to 4800 fmol (Additional file 2**: **Figure S1). The linearity, LOD, and LOQ data for the 64 peptides are presented in Additional file 28: Table S1. The LOD, LOQ, and linearity (represented by R^2^ values of the linear regression models) of the 64 peptides ranged from 0.22 to 95.15 fmol, 0.67 to 285.41 fmol, and 0.9754 to 0.9997, respectively. The calibration curves for 2 peptides from insulin-like growth factor-binding protein 2 (IBP2), peptides IBP2_LEG and IBP2_LIQ, demonstrate linearity across all 11 standard points (Fig. 1A and B). Calibration curves for the remaining 62 target peptides can be found in Additional file 3: Figure S2-S23.

**Fig. 2:**
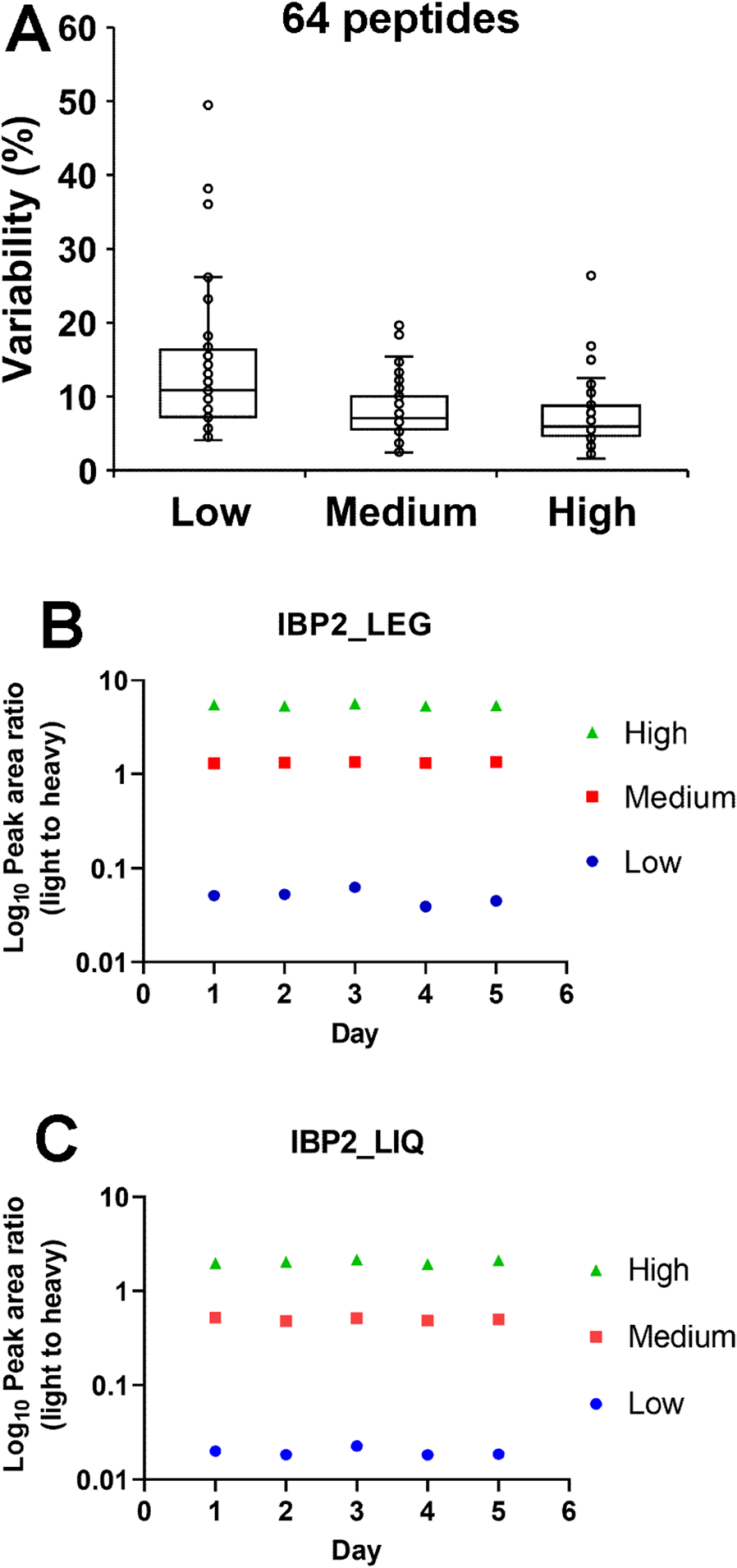
Validation of repeatability demonstrating reproducibility of target peptide measurements. **A** Total variability for 64 peptides at low (2xLOQ), medium (50xLOQ), and high (200xLOQ) levels. **B, C** Light-to-heavy peak area ratio of IBP2_LEG and IBP2_LIQ at low, medium, and high levels across 5 days

To assess the repeatability of the PRM assay, the total CVs of the 64 peptides were determined at each of 3 levels (Fig. 2A). Of the 64 peptides, 56 passed the acceptability criteria of total assay CV < 20%. Among the 64 peptides, 8 did not pass the acceptability criteria at the low level and 1 of the 8 peptides also did not pass the acceptability criteria at the high level (Additional file 29: Table S2). The IBP2_LEG and IBP2_LIQ peptides demonstrated high repeatability at each level over 5 days as shown in Figs. 2B and C. The total assay variability of IBP2_LEG at the low, medium, and high levels over 5 days was 16.80%, 3.40%, and 3.87%, respectively. The total assay variability of IBP2_LIQ at the low, medium, and high levels over 5 days was 13.10%, 6.16%, and 5.45%, respectively (Additional file 29: Table S2). To confirm the specificity of the PRM assay, the responses of 64 peptides were measured in 6 different biological serum replicates using buffer-only (no spike), medium (50 × LOQ), and half-medium (25 × LOQ) levels (selectivity assay). The acceptability criteria were as follows: (1) Individual slopes are within 10% of mean; (2) Observed half-medium level (25 × LOQ) < 10% different from the predicted half-medium level; and (3) Peak area intensity between each transition ion is within 30% of the mean. Of the 64 peptides, 58 peptides (90.6%) showed individual slopes within 10% of the mean (Additional file 30**: **Table S3) and 24 peptides (37.5%) had levels that differed < 10% between the observed 25 × LOQ and predicted 25 × LOQ value (Additional file 31: Table S4). All ion transition ratios for 64 peptides were within 30% of the mean (Additional file 32: Table S5).

To summarize the effort to develop and validate of our PRM assay, the LOD and LOQ of 64 peptides were calculated using calibration curves prepared by spiking digested serum (background matrix) with synthetic light and heavy stable isotope-labeled peptides corresponding to our candidate biomarkers. Repeatability and selectivity experiments were performed according to the CPTAC Tier 2 assay validation guidelines, and 24 of the initial 64 peptides passed the validation acceptability criteria.

### Quantification of candidate biomarkers in HGSOC

The endogenous abundance levels of the 64 target peptides in 69 serum samples (Table 1) from women with non-cancerous conditions (n = 18), benign ovarian conditions (n = 18), early-stage HGSOC (n = 16), and late-stage HGSOC (n = 17) were measured in duplicate using our validated PRM assay. In total, 6 peptides were quantified at levels above their respective LOQs: 2 peptides, IBP2_LEG and IBP2_LIQ, corresponding to insulin-like growth factor-binding protein 2 (IBP2), 2 peptides, SHBG_QAE and SHBG_LVD corresponding to sex hormone-binding globulin (SHBG), and 2 peptides, TIMP1_GFQ and TIMP1_SEE, corresponding to TIMP metalloproteinase inhibitor 1 (TIMP1). From 69 serum samples analyzed in duplicate, only 6 peptides with relative abundance CVs < 20% were selected for further analysis (Additional file 24: Figure S24 and Additional file 33: Table S6). The abundances of the 3 target proteins corresponding to 6 quantified endogenous peptides were calculated based on concentration of the endogenous target peptides within each sample group (Table 4).

**Table 4:** Concentration of 3 target proteins in 69 patients’ serum samples

Protein concentration calculated from concentration of each peptide corresponding to the protein										
UniProt accession #	Peptide sequence	MW (Da)	Peptide concentration in Patients' sera (pmol mL^−1^; mean ± SD)				Protein concentration in Patients' sera (ng mL^−1^; mean ± SD)			
			Non-cancerous	Benign	Early-stage	Late-stage	Non-cancerous	Benign	Early-stage	Late-stage
sp|P18065|IBP2	LEGEACGVYTPR	34814	1.38 ± 0.64	1.32 ± 0.68	1.47 ± 1.05	2.37 ± 1.45	48.10 ± 22.35	46.13 ± 23.50	51.13 ± 36.65	82.44 ± 50.36
	LIQGAPTIR		0.48 ± 0.28	0.48 ± 0.28	0.63 ± 0.46	0.99 ± 0.58	16.60 ± 9.83	16.60 ± 9.83	21.97 ± 16.09	34.45 ± 20.22
sp|P04278|SHBG	QAEISASAPTSLR	43779	8.77 ± 6.04	8.73 ± 4.61	8.56 ± 2.95	10.62 ± 2.95	383.94 ± 264.34	382.03 ± 201.61	374.65 ± 129.13	464.75 ± 129.19
	LDVDQALNR		0.91 ± 0.49	0.54 ± 0.28	0.62 ± 0.31	0.73 ± 0.23	39.72 ± 21.30	23.85 ± 12.31	27.23 ± 13.42	32.13 ± 10.29
sp|P01033|TIMP1	GFQALGDAADIR	23171	0.69 ± 0.10	0.78 ± 0.28	0.83 ± 0.23	1.07 ± 0.43	16.09 ± 2.25	17.98 ± 6.39	19.18 ± 5.39	24.88 ± 9.86
	SEEFLIAGK		1.32 ± 0.14	1.95 ± 1.60	1.69 ± 0.50	1.71 ± 0.60	26.68 ± 10.54	45.20 ± 37.15	39.20 ± 11.56	39.56 ± 13.92

Protein concentration calculated from mean concentration of two peptides corresponding to the protein										
UniProt accession #	Peptide sequence	MW (Da)	Peptide concentration in Patients' sera (pmol mL^−1^; mean ± SD)				Protein concentration in Patients' sera (ng mL^−1^; mean ± SD)			
			Non-cancerous	Benign	Early-stage	Late-stage	Non-cancerous	Benign	Early-stage	Late-stage
sp|P18065|IBP2	LEGEACGVYTPR	34814	0.77 ± 0.51*	0.77 ± 0.40*	1.06 ± 0.73*	1.67 ± 1.00*	26.82 ± 17.82	26.93 ± 14.09	36.83 ± 25.52	58.98 ± 34.67
	LIQGAPTIR									
sp|P04278|SHBG	QAEISASAPTSLR	43779	5.22 ± 3.32*	6.31 ± 4.94*	5.80 ± 2.57*	6.17 ± 1.80*	228.58 ± 145.39	276.05 ± 216.09	254.03 ± 112.43	270.21 ± 78.60
	LDVDQALNR									
sp|P01033|TIMP1	GFQALGDAADIR	23171	0.84 ± 0.21*	1.48 ± 1.49*	1.17 ± 0.38*	1.33 ± 0.43*	19.46 ± 4.88	34.28 ± 34.59	27.13 ± 8.82	30.89 ± 10.02
	SEEFLIAGK									

^*^represents the mean concentration of two peptides corresponding to each target protein

**Fig. 3:**
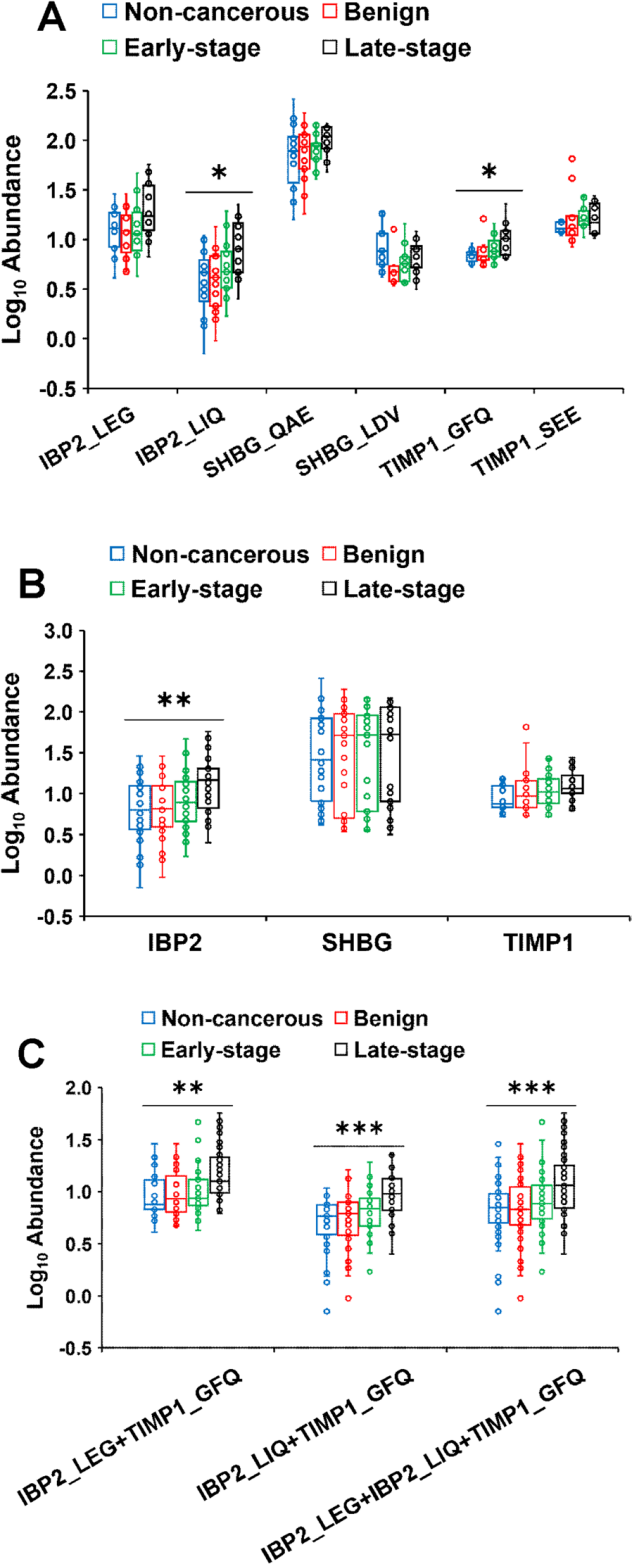
Abundance of six peptides in serum from patient samples. The abundance of each peptide (**A**), each protein (**B**), and combinations of peptides (**C**) was quantified in non-cancerous, benign ovarian condition (benign), early-stage HGSOC (early-stage), and late-stage HGSOC (late-stage) sera using the PRM assay developed in this study. The abundance indicates the on-column concentration based on a 10 µL injection volume. Statistically significant differences were obtained by non-parametric ANOVA. *, **, and *** indicate *p* < 0.05, *p* < 0.01, and *p* < 0.001 respectively

To determine the statistical significance of the differential abundance of each peptide across sample groups, non-parametric ANOVA was performed using mean and interquartile ranges for robustness (Fig. 3). The relative abundances of IBP2_LIQ and TIMP1_GFQ were statistically significant (Fig. 3A). To assess the significance of proteins corresponding to the quantified peptides, all concentration values of peptides from their corresponding proteins were combined, and then statistical analysis was performed in the same manner as in Fig. 3A. The differential abundance of IBP2 including IBP2_LEG and IBP2_LIQ peptides showed statistical significance across the sample groups, but the abundance of SHBG including SHBG_QAE and SHBG_LDV peptides and TIMP1 including TIMP1_GFQ and TIMP1_SEE peptides were not statistically significant (Fig. 3B).

At this stage in our analysis, IBP2 including IBP2_LEG and IBP2_LIQ peptides, were considered candidate diagnostic HGSOC biomarkers since these 2 peptides passed the validation criteria for the repeatability and selectivity assays. TIMP1_GFQ peptide exhibited statistically significant differences in abundance across the sample groups, and failed to meet just 1 of the validation acceptability criteria for the selectivity assays. Namely, the abundance of TIMP1_GFQ exhibited a difference of > 10% between the observed 25 × LOQ and predicted 25 × LOQ in 1 of the 6 different biological serum samples. In spite of this 1 minor failure, we decided to retain TIMP1_GFQ as a diagnostic biomarker candidate along with IBP2_LEG and IBP2_LIQ for further analysis.

The statistical significance of combinations of IBP2_LEG, IBP2_LIQ, and TIMP1_GFQ was investigated by performing non-parametric ANOVA. The 3 combinations from IBP2_LEG, IBP2_LIQ, and TIMP1_GFQ were as follows: (1) IBP2_LEG and TIMP1_GFQ, (2) IBP2_LIQ and TIMP1_GFQ, and (3) IBP2_LEG, IBP2_LIQ, and TIMP1_GFQ. These 3 combinations showed statistically significant differences across the sample groups (Fig. 3C). Interestingly, combination with the TIMP1_GFQ peptide resulted in an increased statistical significance when considering the *p*-value. Based on these results, we expected that IBP2_LEG, IBP2_LIQ, and TIMP1_GFQ would exhibit differential abundance when comparing late-stage vs. non-cancerous or benign sera.

**Fig. 4:**
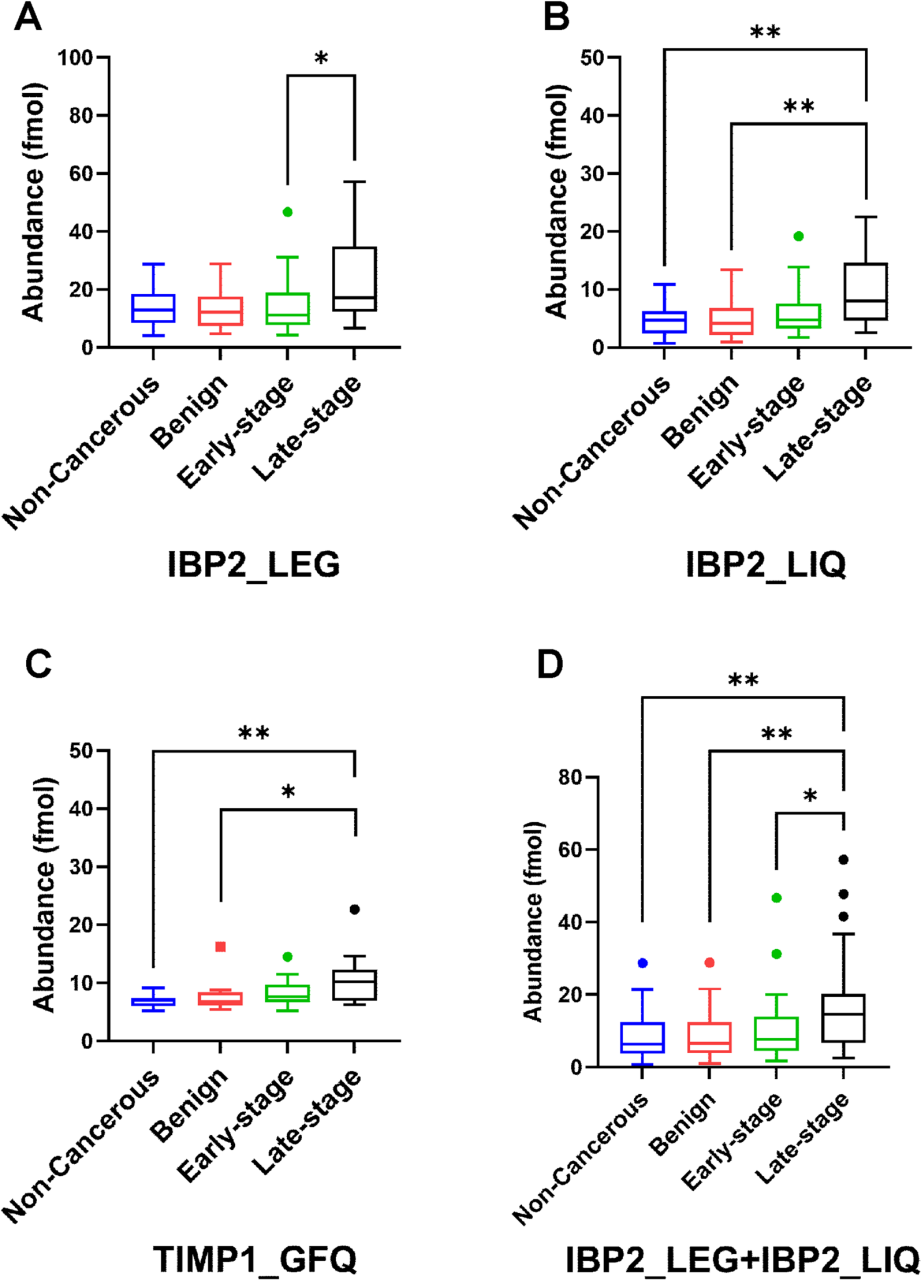
Statistical analysis of relative abundance of IBP2_LEG, IBP2_LIQ, and TIMP1_GFQ peptides in patients’ serum samples. **A** IBP2_LEG peptide. **B** IBP2_LIQ peptide. **C** TIMP1_GFQ peptide. **D** A combination of IBP2_LEG and IBP2_LIQ. A post-hoc test was used for pairwise comparison of the abundance of peptides from non-cancerous, benign ovarian condition (benign), early-stage HGSOC (early-stage), and late-stage HGSOC (late-stage) sera. The abundance indicates the on-column concentration based on a 10 µL injection volume. * indicates *p* < 0.05 and ** indicates *p* < 0.01

Statistically significant differences of the 3 peptides between sample groups were estimated by a post-hoc test (Fig. 4). IBP2_LEG showed significant differences in abundance when comparing early-stage vs. late-stage sera (Fig. 4A). IBP2_LIQ and TIMP1_GFQ were significant when comparing non-cancerous vs. late-stage and benign vs. late-stage sera (Fig. 4B and C). A combination of IBP2_LEG and IBP2_LIQ was significant when comparing non-cancerous vs. late-stage, benign vs. late-stage, and early-stage vs. late-stage sera (Fig. 4D). However, SHBG_QAE, SHBG_LDV, and TIMP1_SEE did not discriminate late-stage from non-cancerous or benign **(**Additional file 25: Figure S25 and Additional file 26: S26).

Overall, our results suggest that the serum levels of IBP2_LEG, IBP2_LIQ, and TIMP1_GFQ, as quantified by our multiplexed PRM assay, could discriminate late-stage HGSOC from non-cancerous or benign ovarian conditions.

### Diagnostic performance of candidate biomarkers

**Fig. 5:**
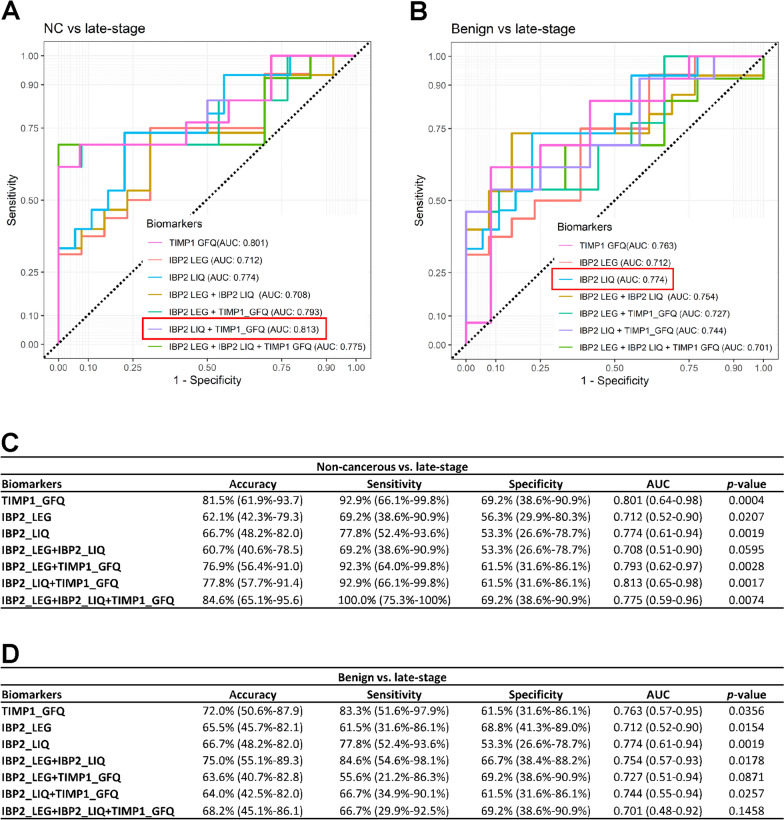
Evaluation of identified candidate diagnostic biomarkers in HGSOC. **A, B** Comparison of receiver operating characteristic (ROC) curves from IBP2_LEG, IBP2_LIQ, TIMP1_GFQ, and combinations of the three peptides in non-cancerous vs. late-stage and benign vs. late-stage sera. **C, D** Summary of diagnostic performance of IBP2_LEG, IBP2_LIQ, TIMP1_GFQ, and combinations of the three peptides in non-cancerous vs. late-stage and benign vs. late-stage sera

To evaluate the diagnostic performance of individual peptides and combinations of peptides in HGSOC, we determined AUC values from ROC curves for IBP2_LEG, IBP2_LIQ, TIMP1_GFQ, and combinations of the 3 peptides in non-cancerous vs. late-stage HGSOC and benign vs. late-stage HGSOC conditions (Fig. 5). When comparing non-cancerous vs. late-stage sera, the AUCs of IBP2_LEG, IBP2_LIQ, and TIMP1_GFQ were 0.712, 0.774, and 0.801, respectively. IBP2_LEG and IBP2_LIQ showed improved diagnostic performance when they were combined with TIMP1_GFQ than when they were analyzed as individual peptides (AUC of IBP2_LEG and TIMP1_GFQ: 0.793, AUC of IBP2_LIQ and TIMP1_GFQ: 0.813). The highest AUC of 0.813 was achieved from a combination of IBP_LIQ and TIMP1_GFQ (Fig. 5A and C). When comparing benign vs. late-stage sera, the AUCs of IBP2_LEG, IBP2_LIQ, and TIMP1_GFQ were 0.712, 0.774, and 0.763, respectively. Interestingly, combinations with TIMP1_GFQ did not show any synergistic effect when comparing benign vs. late-stage conditions. The highest AUC of 0.774 was achieved from IBP2_LIQ alone (Fig. 5B and D). The diagnostic performance of IBP2_LEG, IBP2_LIQ, TIMP1_GFQ, and combinations of peptides was assessed based on accuracy, sensitivity, specificity, AUC, and *p*-value (Fig. 5C and D). An important caveat of this analysis is that the confidence intervals around the sensitivity, specificity, and AUC of these peptides nearly completely overlap, suggesting a lack of statistical significance when evaluating their diagnostic performance.

CA125 (MUC16) is commonly used for clinical HGSOC diagnostic tests. However, the specificity (50%) and sensitivity (78%) of CA125 are not adequate in clinical applications to screen for HGSOC at early disease stages [40]. To overcome the limitation of using CA125 alone, approaches combining CA125 with other biomarkers have exhibited an improvement in the diagnosis of ovarian cancer. In vitro Diagnostic Multivariate Index Assays (IVDMIA) such as OVA1, ROMA, and OVERA that include multiple biomarkers demonstrate improved clinical performance vs. CA125 alone, indicating that the best diagnostic method for ovarian cancer could entail the combination of multiple biomarkers [41–46]. Therefore, we evaluated the diagnostic performance of combining the clinical measurements of CA125 that were obtained from patients’ medical records with our biomarker candidates IBP2_LEG and IBP2_LIQ in benign conditions vs. late-stage HGSOC compared to using CA125 alone (Additional file 34: Table S7). The accuracy, sensitivity, specificity, and AUC of CA125 to discriminate late-stage HGSOC from benign ovarian conditions were 94.3%, 94.4%, 94.1%, and 0.987 respectively. The combination of CA125, IBP2_LEG, and IBP2_LIQ showed improved accuracy (96.4%) and sensitivity (100%) with slightly lower specificity (93.3%) and AUC (0.985) compared to CA125 alone. Although the CA125 values alone had outstanding performance in our sample groups, the addition of our biomarker candidates IBP2_LEG and IBP2_LIQ increased the accuracy and sensitivity to detect late-stage HGSOC while maintaining similar specificity and AUC.

The sensitivity and specificity of the diagnostic performance of CA125 in our patient cohort is higher than the literature-reported values of 78% and 50%, respectively [40], due to the markedly elevated serum levels of CA125 in the cancer cases in our cohort who were all diagnosed with HGSOC. Further studies are required to evaluate the diagnostic performance of our biomarker candidates, including their combined diagnostic performance with CA125, using a larger number of samples inclusive of women who do not have elevated CA125 levels.

Overall, the AUC of individual peptides and combinations of peptides was > 0.712 when evaluating the differentiation between late-stage vs. non-cancerous or benign conditions. These results suggest that IBP2_LEG, IBP2_LIQ, and TIMP1_GFQ measured using our PRM assay could have clinical utility for HGSOC diagnosis.

## Discussion

The overall incidence of ovarian cancer in the U.S. has been gradually decreasing since 1975 when the number of cases per 100,000 individuals was 15.9 [47]. In 2020, the incidence per 100,000 individuals had fallen to 9.2. The overall ovarian cancer mortality has also decreased. However, these statistics do not disclose the racial, ethnic, age, menopausal status, and socioeconomic disparities related to ovarian cancer incidence, mortality, survival rates, and diagnosis [32–36]. An acknowledged limitation of our study is the over-representation of non-Hispanic White women.

Ovarian cancer is a disease primarily of post-menopausal women. It can often be difficult for women within this demographic to receive timely and effective treatment when seeking medical treatment due to non-specific symptoms of ovarian cancer. Rather unfortunately, these women are often faced with an unduly prolonged medical journey while suffering from undiagnosed ovarian cancer. An effective biomarker-based diagnostic test would have the potential to improve the quality of life for all women who have nonspecific symptoms of ovarian cancer and consequently do not receive effective medical care.

**Fig. 6:**
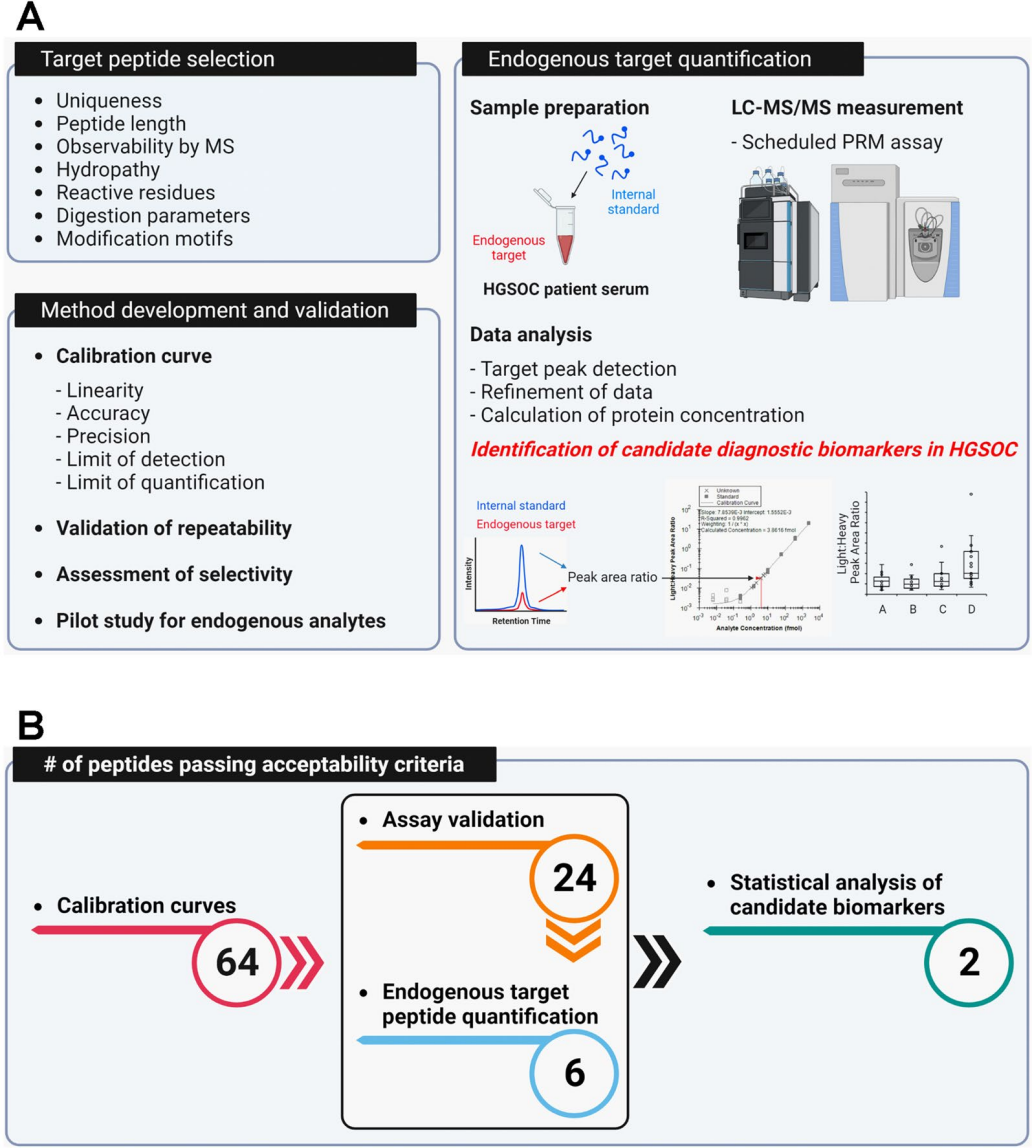
Workflow of method development and validation to quantify target proteins using PRM assay in HGSOC. **A** Schematic of the experimental design. This study is divided into (1) Target peptide selection, (2) Method development and validation, and (3) Endogenous target quantification to identify candidate diagnostic biomarkers in HGSOC. **B** Assay validation acceptability criteria. The flowchart shows the number of peptides passing acceptability criteria

To identify and quantify candidate diagnostic biomarkers of HGSOC, in the current study, we sought to develop and validate a PRM assay in three phases as follows: (1) target peptide selection, (2) method development and validation, and (3) endogenous target quantification (Fig. 6A). In the first phase, 64 target peptides were selected from 23 serum protein ovarian cancer biomarker candidates. Stable isotope (^13^C/^15^N)-labeled heavy peptides on their C-terminal Lys or Arg and corresponding unlabeled light peptides were chemically synthesized. In the second phase, a PRM assay was characterized via generating calibration curves and repeatability and selectivity assay according to CPTAC Tier 2 assay validation guidelines. In the third phase, endogenous target peptides were quantified using our validated PRM method in 69 serum samples including 18 non-cancerous conditions, 18 benign ovarian conditions, 16 early-stage HGSOC, and 17 late-stage HGSOC serum samples. The target peptides were subjected to assay validation acceptability criteria to identify candidate diagnostic biomarkers for method development and validation and endogenous target quantification (Fig. 6B). The calibration curves for 64 light peptides corresponding to 23 candidate protein biomarkers were generated and linearity, LOD, and LOQ were measured using the PRM assay. The repeatability and specificity of the PRM assay for 64 target peptides were validated according to acceptability criteria. Of the initial 64 peptides, 24 peptides passed the validation acceptability criteria. Of 24 peptides, 6 peptides were quantified using the validated PRM assay. IBP2 (namely IBP2_LEG and IBP2_LIQ peptides) was identified as a diagnostic HGSOC biomarker candidate.

During our analytical validation of the PRM assay, we did not evaluate the potential presence of matrix effects related to the presence of salts, lipids, metabolites and incompletely-digested proteins. It is possible that matrix effects could have influenced the lack of detection of some of the candidate biomarkers. Additionally, many of the candidate biomarkers are glycoproteins, and the presence of glycans of various sizes and structures could have impeded the proteolytic digestion of the proteins and the MS detection of the target peptides.

One of the most significant challenges in the quantification of biomarkers using PRM is the lack of sufficient sensitivity for detecting low abundance peptides/proteins in complex biological samples. Therefore, several methods have been used to improve the detection capabilities of low abundance peptides/proteins by reducing the sample complexity: (1) immunoaffinity enrichment of low abundance proteins of interest, (2) immunodepletion of high abundance proteins, and (3) fractionation to reduce the sample complexity [48]. However, these approaches tend to introduce variability during sample preparation [49]. Refined enrichment and fractionation methods can enhance an analyte’s LOQ by 50 – 100-fold [50]. Utilizing a simplified sample preparation workflow will be beneficial in terms of translating this type of assay to a clinical laboratory.

The diagnostic performance of the IBP2_LEG and IBP2_LIQ peptides corresponding to IBP2 protein was evaluated based on the AUC values from ROC curves. We also evaluated the diagnostic performance of the TIMP1_GFQ peptide corresponding to the TIMP1 protein, even though TIMP1_GFQ did not pass 1 of the 3 validation acceptability criteria in the selectivity experiment. The TIMP1_GFQ peptide exhibited a difference of > 10% between the observed 25 × LOQ and predicted 25 × LOQ in 1 of the 6 different serum specimens that were used for the selectivity experiment.

Insulin-like growth factor-binding protein 2 (IBP2) is considered to be an oncogene and it has been proposed as a potential biomarker in various cancers including ovarian, glioma, prostate, colorectal, pancreatic, breast, and liver [52, 53]. Although the function of IBP2 in cancers is not clear, high expression of IBP2 has been associated with *PTEN* mutations in glioblastoma, breast, and prostate cancers [51–54] and *KRAS* mutations in lung cancer [55]. IBP2 promotes cell proliferation and cancer cell invasion, and it suppresses apoptosis [56–58]. In addition, IBP2 overexpression has been demonstrated in HGSOC compared to other types of ovarian tumors and normal surface epithelium, indicating that IBP2 is differentially regulated in different types of ovarian cancer [59]. This might suggest that IBP2 expression correlates with tumor stage and subtype and has an oncogenic function in the development and progression of HGSOC.

Metalloproteinase inhibitor 1 (TIMP1) has been implicated in various biological processes, including cancers [60–62]. TIMP1 might be important for tumor growth and metastasis. In the analysis of circulating tumor cells from patients with advanced stage HGSOC, TIMP1 has been suggested to be used as a therapeutic target. TIMP1-deficient conditions decreased tumor growth in vitro and in vivo [63]. Ovarian cancer often develops resistance to chemotherapy, leading to treatment failure. TIMP-1 is associated with chemotherapy resistance in ovarian cancer cells [64]. However, the mechanisms of how TIMP1 contributes to ovarian cancer are not yet fully understood.

Interestingly, we found that the mass spectrometry response of peptides derived from the same protein differed among our sample groups: non-cancerous, benign ovarian condition, early-stage HGSOC, and late-stage HGSOC. Considering the AUC values, a combination of IBP2_LEG and IBP2_LIQ peptides did not show a synergistic effect when comparing non-cancerous vs. late-stage and benign vs. late-stage sera. The IBP2_LIQ peptide alone showed a slightly higher AUC of 0.774 compared to the combination of IBP2_LEG and IBP2_LIQ peptides (AUC: 0.708 and 0.754 in non-cancerous vs. late-stage HGSOC and benign vs. late-stage HGSOC, respectively).

Similar to IBP2_LEG and IBP2_LIQ peptides, the diagnostic performance of the 2 TIMP1_GFQ and TIMP1_SEE peptides corresponding to TIMP1 proteins was not identical. TIMP1_GFQ exhibited a significant difference in abundance comparing the non-cancerous vs. late-stage HGSOC and benign vs. late-stage HGSOC sera. The TIMP1_GFQ peptide also exhibited good diagnostic performance as a single biomarker. However, the TIMP1_SEE peptide could not pass most of the assay validation acceptability criteria, indicating that the TIMP1_SEE peptide is not suitable for the quantification of TIMP1 protein. The total assay variability of TIMP1_SEE was 49.40% at the low concentration in the repeatability experiment. This peptide had slopes that were not within 10% of the mean in 2 of the 6 different biological sera that were used for the selectivity experiment, and it exhibited a difference of > 10% between the observed 25 × LOQ and predicted 25 × LOQ, indicating that TIMP1_SEE failed the assay validation acceptability criteria in 5 out of 6 different biological sera for the selectivity experiment. Potentially related to its sub-optimal analytical performance, TIMP1_SEE did not discriminate late-stage HGSOC from non-cancerous or benign conditions. It is important to note that biochemical and technical reasons could account for the discrepant performance of the 2 TIMP1 peptides. Incomplete release of the TIMP1_SEE peptide from the TIMP1 protein could have occurred during the process of enzymatic digestion with trypsin. Furthermore, the MS detection of this peptide could have been negatively impacted by the presence of peptides of similar masses that chromatographically co-eluted and yielded product ion fragments that were similar to those from the target peptide of interest.

This discrepancy between the analytical performance of peptides derived from same protein could be due to the different MS response of each peptide. Similarly, in another study that used a PRM assay to quantify proteins extracted from formalin-fixed paraffin-embedded tissue slides, multiple peptides from the HER2 protein exhibited differential performance in discriminating breast cancer subtypes among a cohort of 51 patients [65]. This report suggests that selecting target peptides that exhibit good MS response to represent target proteins is a critical factor to maximize the diagnostic performance of biomarker protein candidates. Therefore, selecting multiple peptides for each target protein prior to developing a targeted MS assay will be essential to finalize the selection of the best peptide for a target protein.

## Conclusion

PRM allows the simultaneous quantification of multiple target peptides/proteins in complex biological samples such as serum, that may prove useful in diagnostic biomarker development due to its: (1) high sensitivity, specificity, accuracy, and reproducibility, (2) multiplexing capability, and (3) utility in the validation and verification of biomarkers using a large number of samples [66, 67]. PRM assays that are developed through rigorous assay optimization can facilitate the development and implementation of diagnostic biomarkers for clinical utility.

One reason for the inability of candidate biomarkers to achieve clinical utility is their low specificity in large cohorts. To improve the clinical utility of our assay as a screening tool for women in the general population and to increase the specificity of the diagnostic performance of our assay, it would be essential to include sera from women who do not have elevated levels of CA125. For example, a nested case–control study using samples from the United Kingdom Collaborative Trial of Ovarian Cancer Screening trial, including serial samples from women up to 7 years pre-diagnosis, demonstrated that an ELISA-based combined biomarker panel comprised of IGFBP2, LCAT and CA125 outperformed the performance of CA125 alone up to 3 years pre-diagnosis [68]. Another strategy to improve the diagnostic performance of our PRM assay would be to stratify our patient cohort by menopausal status and to age-match the cases and controls, thereby enhancing the assay’s clinical utility.

In our study, we sought to develop and validate a multiplexed targeted MS PRM assay for diagnostic biomarker analysis in HGSOC. IBP2 was identified as a diagnostic biomarker candidate to differentiate late-stage HGSOC patients from healthy controls and patients with benign ovarian conditions. Our study was conducted using a limited sample size (n = 69); therefore, additional studies need to be performed using a significantly larger number of samples to confirm the diagnostic performance of the IBP2 PRM assay, particularly in detecting early-stage HGSOC. The frontier of clinical diagnostics is expanding to encompass new technologies such as PRM MS in clinical laboratories.

### Supplementary Information

**Additional file 1: File S1. **Quarto document with scripts used for candidate biomarker data analysis. The ROC curves can be generated after rendering the document using the data in Table S6 (Concentration of 6 endogenous peptides corresponding to 3 proteins in serum samples from 69 patients).

**Additional file 2: Figure S1.** Calibration curve-based assay performance for 64 peptides. **A** LOD and LOQ. The horizontal red and blue lines in each box represent the median peptide LOD and LOQ in each matrix. The y-axis indicates the on-column concentration based on a 10 µL injection volume. The median LOD and LOQ is included in the table below the plot. **B **Evaluation of the accuracy of the observed abundance of each peptide standard. Enlarged range of the calibration curve from 6 - 4800 fmol.

**Additional file 3: Figure S2.** Calibration curves of peptides corresponding to MUC16 protein. Gray square indicates each standard point. White square indicates standard points that were excluded from calibration curve with the accuracy of > 20%. Black line indicates the calibration curves.

**Additional file 4: Figure S3.** Calibration curves of peptides corresponding to CADH3 protein. Gray square indicates each standard point. White square indicates standard points that were excluded from calibration curve with the accuracy of > 20%. Black line indicates the calibration curves.

**Additional file 5: Figure S4.** Calibration curves of peptides corresponding to FETA protein. Gray square indicates each standard point. White square indicates standard points that were excluded from calibration curve with the accuracy of > 20%. Black line indicates the calibration curves.

**Additional file 6: Figure S5.** Calibration curves of peptides corresponding to FGF2 protein. Gray square indicates each standard point. White square indicates standard points that were excluded from calibration curve with the accuracy of > 20%. Black line indicates the calibration curves.

**Additional file 7: Figure S6.** Calibration curves of peptides corresponding to FOLR1 protein. Gray square indicates each standard point. White square indicates standard points that were excluded from calibration curve with the accuracy of > 20%. Black line indicates the calibration curves.

**Additional file 8: Figure S7.** Calibration curves of peptides corresponding to FST protein. Gray square indicates each standard point. White square indicates standard points that were excluded from calibration curve with the accuracy of > 20%. Black line indicates the calibration curves.

**Additional file 9: Figure S8.** Calibration curves of peptides corresponding to GDF15 protein. Gray square indicates each standard point. White square indicates standard points that were excluded from calibration curve with the accuracy of > 20%. Black line indicates the calibration curves.

**Additional file 10: Figure S9.** Calibration curves of peptides corresponding to IBP1 protein. Gray square indicates each standard point. White square indicates standard points that were excluded from calibration curve with the accuracy of > 20%. Black line indicates the calibration curves.

**Additional file 11: Figure S10.** Calibration curves of peptides corresponding to IL6 protein. Gray square indicates each standard point. White square indicates standard points that were excluded from calibration curve with the accuracy of > 20%. Black line indicates the calibration curves.

**Additional file 12: Figure S11.** Calibration curves of peptides corresponding to KLK6 protein. Gray square indicates each standard point. White square indicates standard points that were excluded from calibration curve with the accuracy of > 20%. Black line indicates the calibration curves.

**Additional file 13: Figure S12.** Calibration curves of peptides corresponding to KLK11 protein. Gray square indicates each standard point. White square indicates standard points that were excluded from calibration curve with the accuracy of > 20%. Black line indicates the calibration curves.

**Additional file 14: Figure S13.** Calibration curves of peptides corresponding to K1C19 protein. Gray square indicates each standard point. White square indicates standard points that were excluded from calibration curve with the accuracy of > 20%. Black line indicates the calibration curves.

**Additional file 15: Figure S14.** Calibration curves of peptides corresponding to MSLN protein. Gray square indicates each standard point. White square indicates standard points that were excluded from calibration curve with the accuracy of > 20%. Black line indicates the calibration curves.

**Additional file 16: Figure S15.** Calibration curves of a peptide corresponding to MUC1 protein. Gray square indicates each standard point. White square indicates standard points that were excluded from calibration curve with the accuracy of > 20%. Black line indicates the calibration curves.

**Additional file 17: Figure S16.** Calibration curves of peptides corresponding to NECT4 protein. Gray square indicates each standard point. White square indicates standard points that were excluded from calibration curve with the accuracy of > 20%. Black line indicates the calibration curves.

**Additional file 18: Figure S17.** Calibration curves of peptides corresponding to OSTP protein. Gray square indicates each standard point. White square indicates standard points that were excluded from calibration curve with the accuracy of > 20%. Black line indicates the calibration curves.

**Additional file 19: Figure S18.** Calibration curves of peptides corresponding to PRL protein. Gray square indicates each standard point. White square indicates standard points that were excluded from calibration curve with the accuracy of > 20%. Black line indicates the calibration curves.

**Additional file 20: Figure S19.** Calibration curves of peptides corresponding to PRSS8 protein. Gray square indicates each standard point. White square indicates standard points that were excluded from calibration curve with the accuracy of > 20%. Black line indicates the calibration curves.

**Additional file 21: Figure S20.** Calibration curves of peptides corresponding to SHBG protein. Gray square indicates each standard point. White square indicates standard points that were excluded from calibration curve with the accuracy of > 20%. Black line indicates the calibration curves.

**Additional file 22: Figure S21.** Calibration curves of peptides corresponding to TIMP1 protein. Gray square indicates each standard point. White square indicates standard points that were excluded from calibration curve with the accuracy of > 20%. Black line indicates the calibration curves.

**Additional file 23: Figure S22.** Calibration curves of a peptide corresponding to TNR6 protein. Gray square indicates each standard point. White square indicates standard points that were excluded from calibration curve with the accuracy of > 20%. Black line indicates the calibration curves.

**Additional file 24: Figure S23.** Calibration curves of peptides corresponding to VGFR2 protein. Gray square indicates each standard point. White square indicates standard points that were excluded from calibration curve with the accuracy of > 20%. Black line indicates the calibration curves.

**Additional file 25: Figure S24.** Evaluation of precision of quantified peptides. Variability for each peptide from all 69 serum samples including non-cancerous (n=18), benign ovarian condition (n=18), early-stage serous ovarian (n=16), and late-stage serous ovarian (n=17) patients.

**Additional file 26: Figure S25.** Statistical analysis of SHBG_QAE and SHBG_LDV corresponding to SHBG protein. **A** SHBG_QAE peptide. **B** SHBG_LDV peptide. A post-hoc test was used for pairwise comparison of the abundance of peptides from non-cancerous, benign ovarian condition (benign), early-stage serous ovarian cancer (early-stage), and late-stage serous ovarian cancer (late-stage) sera. The abundance indicates the on-column concentration based on an injection volume of 10 µL. * indicates *p* < 0.05.

**Additional file 27: Figure S26.** Statistical analysis of TIMP1_SEE corresponding to TIMP1 protein. The abundance indicates the on-column concentration based on an injection volume of 10 µL.

**Additional file 28: Table S1. **Linearity, LOD, and LOQ values for the 64 peptides corresponding to the 23 proteins.

**Additional file 29: Table S2. **Validation of repeatability to evaluate the PRM assay performance.

**Additional file 30: Table S3. **Selectivity to evaluate the PRM assay specificity of the 64 peptides: Slope values.

**Additional file 31: Table S4. **Selectivity to evaluate the PRM assay specificity of the 64 peptides: Half-medium light-to-heavy ratios.

**Additional file 32: Table S5. **Selectivity to evaluate the PRM assay specificity of the 64 peptides: Ion transition ratios.

**Additional file 33: Table S6. **Concentration of 6 endogenous peptides corresponding to 3 proteins in serum samples from 69 patients.

**Additional file 34: Table S7. **Evaluation of diagnostic performance of IBP2_LEG and IBP2_LIQ with CA125.

## Data Availability

The mass spectrometry proteomics data have been deposited to the ProteomeXchange Consortium via the PRIDE [69] partner repository with the dataset identifier PXD047188. All of the annotated Skyline data files from the PRM MS datasets (extracted ion chromatograms from the calibration curves, repeatability experiment, selectivity experiment, and endogenous quantification) are available via Panorama Public using the following link: https://panoramaweb.org/D1WcWz.url. The analyzed data are included as Supplementary Information.

## Abbreviations

HGSOC: High-grade serous ovarian cancer
PRM: Parallel reaction monitoring
HRAM: High-resolution accurate mass
MS: Mass spectrometer
CPTAC: Clinical Proteomic Tumor Analysis Consortium
LOD: Limit of detection
LOQ: Limit of quantification
ROC: Receiver operating characteristic
AUC: Area under the curve
CI: Confidence interval
IBP2: Insulin-like growth factor-binding protein 2
SHBG: Sex hormone-binding globulin
TIMP1: Metalloproteinase inhibitor 1
IVDMIA: In vitro diagnostic multivariate index assay
