# Supplementary material for: Quantification of putative ovarian cancer serum protein biomarkers using a multiplexed targeted mass spectrometry assay
Source: Clin Proteomics. 2024 Jan 3;21:1. doi: 10.1186/s12014-023-09447-4 (PMC10762856; doi:10.1186/s12014-023-09447-4)
Supplement: Supplementary file 1 — Additional file 1: File S1. Quarto document with scripts used for candidate biomarker data analysis. The ROC curves can be generated after rendering the document using the data in Table S6 (Concentration of 6 endogenous peptides corresponding to 3 proteins in serum samples from 69 patients). [file 12014_2023_9447_MOESM1_ESM.docx]

Statistical methods for cancer protein biomarkers

## Overview

The statistics section is separated into sections, which are expanded with additional comments and code, below. The sections are as follows:

1. All quantitative data are presented as the mean ± standard deviation (S.D.). R version 1.2.2 (2022-10-31 UCRT) was used for statistical analysis.
2. Statistically significant differences of each target peptide in the sample groups were estimated by non- parametric ANOVA or pairwise post-hoc test. p-values $\leq$ 0.05 from ANOVA and post-hoc tests were considered to indicate statistical significance.
3. To evaluate candidate biomarkers with receiver operating characteristic (ROC) curves, the data was normalized to ensure that the concentrations of all peptides and proteins were on the same scale.
4. Logistic regression classifiers were used to determine the propensity scores for cancer stages from various combinations of biomarkers. The scores were used to generate ROC curves and calculate areas under curves (AUC). The p-values for the AUC were calculated from the whole model tests of the logistic regressions.
5. Accuracy, sensitivity, and specificity, and their 95% Delong confidence intervals (CI), for combinations of biomarkers, were calculated using a 0.5 cutoff based on the scores from the logistic regressions.

## The data for this document

To keep the analysis understandable and organized, we included one example of each statistical analysis (omitting graph generation) at the beginning of the document, but included the entire analysis in the **Appendix**. The logistic regression part of this document describes the analysis of investigating the three peptides to distinguish between no cancer and late-stage cancer cases, but all the regressions are in the appendix.

The R code for the plots reported in the manuscript are in the Quarto document, but the plot themselves were exported to files, and do not appear in the rendered Quarto document. The quality of some of the plots in the manuscript were improved using another software program.

## The analysis

### Section 1

Statistical analysis All quantitative data are presented as the mean ± standard deviation (S.D.). R version 1.2.2 (2022-10-31 UCRT) was used for statistical analysis. See [Table 2](#tbl-2), below.

### Section 2

Statistically significant differences of each target peptide in the sample groups were estimated by non-parametric ANOVA or pairwise post-hoc tests. p-values $\leq$ 0.05 from ANOVA and post-hoc tests were considered to indicate statistical significance.

Table 1 Summaries by group. Medians and interquartile ranges used for robustness. The p-values are for non-parametric ANOVA. P<0.05 indicates that at least one group (NC, L, B, E) is different from another group.

mycontrols <- tableby.control(test=TRUE, total=FALSE,
 numeric.test="kwt", cat.test="chisq",
 numeric.stats=c("N", "median", "q1q3"),
 # numeric.stats=c("N", "meansd"),
 cat.stats=c("countpct"),
 stats.labels=list(N='Count', median='Median', q1q3='Q1,Q3'))
tab2 <- tableby(sample_group ~ ., data=df, control=mycontrols) #-2 removes sample id
summary(tab2)

Table 1: Data Summaries and Kruskal-Wallis tests

|  | Benign (N=18) | Early-stage (N=16) | Late-stage (N=17) | Non-cancerous (N=18) | p value |
| --- | --- | --- | --- | --- | --- |
| **ibp_leg** |  |  |  |  | 0.091 |
| Count | 13 | 16 | 16 | 13 |  |
| Median | 12.244 | 11.244 | 17.180 | 12.937 |  |
| Q1,Q3 | 8.618, 16.591 | 7.736, 16.648 | 14.022, 31.604 | 8.744, 18.096 |  |
| **ibp_liq** |  |  |  |  | 0.023 |
| Count | 18 | 14 | 15 | 18 |  |
| Median | 4.684 | 4.712 | 8.003 | 4.684 |  |
| Q1,Q3 | 2.804, 5.697 | 3.442, 6.831 | 5.350, 14.106 | 2.804, 5.697 |  |
| **shbg_qae** |  |  |  |  | 0.264 |
| Count | 15 | 16 | 16 | 16 |  |
| Median | 85.863 | 88.184 | 109.347 | 77.909 |  |
| Q1,Q3 | 53.681, 109.987 | 66.177, 92.057 | 84.343, 133.726 | 51.386, 106.138 |  |
| **shbg_ldv** |  |  |  |  | 0.067 |
| Count | 8 | 11 | 14 | 11 |  |
| Median | 4.701 | 5.712 | 7.995 | 7.698 |  |
| Q1,Q3 | 3.798, 5.140 | 3.839, 6.555 | 5.710, 8.391 | 5.750, 9.993 |  |
| **timp1_gfq** |  |  |  |  | 0.022 |
| Count | 12 | 15 | 13 | 14 |  |
| Median | 6.818 | 7.628 | 10.196 | 7.006 |  |
| Q1,Q3 | 6.272, 8.102 | 6.789, 9.211 | 7.057, 11.989 | 6.250, 7.453 |  |
| **timp1_see** |  |  |  |  | 0.472 |
| Count | 12 | 12 | 10 | 7 |  |
| Median | 12.986 | 15.232 | 14.821 | 12.886 |  |
| Q1,Q3 | 11.489, 16.848 | 14.245, 17.846 | 11.702, 22.224 | 12.369, 14.235 |  |

Table 2 Summaries by group, means and standard deviations. The p-values are for ANOVA. P<0.05 indicates that at least one group is different from another group.

mycontrols <- tableby.control(test=TRUE, total=FALSE,
 numeric.test="kwt", cat.test="chisq",
 #numeric.stats=c("N", "median", "q1q3"),
 numeric.stats=c("N", "meansd", "meanse"),
 cat.stats=c("countpct"),
 stats.labels=list(N='Count', median='Median', q1q3='Q1,Q3'))
tab2 <- tableby(sample_group ~ ., data=df, control=mycontrols)
summary(tab2)

Table 2: Data Summaries and Kruskal-Wallis tests

|  | Benign (N=18) | Early-stage (N=16) | Late-stage (N=17) | Non-cancerous (N=18) | p value |
| --- | --- | --- | --- | --- | --- |
| **ibp_leg** |  |  |  |  | 0.091 |
| Count | 13 | 16 | 16 | 13 |  |
| Mean (SD) | 13.250 (7.026) | 14.685 (10.871) | 23.679 (14.941) | 13.817 (6.683) |  |
| Mean (SE) | 13.250 (1.949) | 14.685 (2.718) | 23.679 (3.735) | 13.817 (1.853) |  |
| **ibp_liq** |  |  |  |  | 0.023 |
| Count | 18 | 14 | 15 | 18 |  |
| Mean (SD) | 4.769 (2.905) | 6.310 (4.798) | 9.895 (6.012) | 4.769 (2.905) |  |
| Mean (SE) | 4.769 (0.685) | 6.310 (1.282) | 9.895 (1.552) | 4.769 (0.685) |  |
| **shbg_qae** |  |  |  |  | 0.264 |
| Count | 15 | 16 | 16 | 16 |  |
| Mean (SD) | 87.263 (47.668) | 85.576 (30.464) | 106.158 (30.476) | 87.699 (62.361) |  |
| Mean (SE) | 87.263 (12.308) | 85.576 (7.616) | 106.158 (7.619) | 87.699 (15.590) |  |
| **shbg_ldv** |  |  |  |  | 0.067 |
| Count | 8 | 11 | 14 | 11 |  |
| Mean (SD) | 5.447 (3.006) | 6.221 (3.215) | 7.340 (2.438) | 9.074 (5.103) |  |
| Mean (SE) | 5.447 (1.063) | 6.221 (0.969) | 7.340 (0.652) | 9.074 (1.539) |  |
| **timp1_gfq** |  |  |  |  | 0.022 |
| Count | 12 | 15 | 13 | 14 |  |
| Mean (SD) | 7.761 (2.881) | 8.279 (2.407) | 10.739 (4.430) | 6.943 (1.008) |  |
| Mean (SE) | 7.761 (0.832) | 8.279 (0.621) | 10.739 (1.229) | 6.943 (0.269) |  |
| **timp1_see** |  |  |  |  | 0.472 |
| Count | 12 | 12 | 10 | 7 |  |
| Mean (SD) | 19.508 (16.746) | 16.917 (5.210) | 17.071 (6.333) | 13.160 (1.524) |  |
| Mean (SE) | 19.508 (4.834) | 16.917 (1.504) | 17.071 (2.003) | 13.160 (0.576) |  |

These are pairwise Wilcoxon rank-sum tests. False Discovery is corrected with the Holm method.

- The first column is the biomarker
- The second and third columns are the pairs of groups being tested.
- The adjusted p-value is in the second-to-last column.
- The last column gives a “heads up” about which pairs of groups are statistically significant.

pairwise_wilcox_test(df,ibp_leg ~ sample_group, p.adjust.method = "holm")%>%
 kable() %>% kable_paper()

| .y. | group1 | group2 | n1 | n2 | statistic | p | p.adj | p.adj.signif |
| --- | --- | --- | --- | --- | --- | --- | --- | --- |
| ibp_leg | Benign | Early-stage | 13 | 16 | 106 | 0.948 | 1.000 | ns |
| ibp_leg | Benign | Late-stage | 13 | 16 | 60 | 0.056 | 0.278 | ns |
| ibp_leg | Benign | Non-cancerous | 13 | 13 | 78 | 0.762 | 1.000 | ns |
| ibp_leg | Early-stage | Late-stage | 16 | 16 | 71 | 0.032 | 0.191 | ns |
| ibp_leg | Early-stage | Non-cancerous | 16 | 13 | 96 | 0.746 | 1.000 | ns |
| ibp_leg | Late-stage | Non-cancerous | 16 | 13 | 148 | 0.056 | 0.278 | ns |

pairwise_wilcox_test(df,ibp_liq ~ sample_group, p.adjust.method = "holm")%>%
 kable() %>% kable_paper()

| .y. | group1 | group2 | n1 | n2 | statistic | p | p.adj | p.adj.signif |
| --- | --- | --- | --- | --- | --- | --- | --- | --- |
| ibp_liq | Benign | Early-stage | 18 | 14 | 106 | 0.464 | 1.000 | ns |
| ibp_liq | Benign | Late-stage | 18 | 15 | 61 | 0.007 | 0.040 | * |
| ibp_liq | Benign | Non-cancerous | 18 | 18 | 162 | 1.000 | 1.000 | ns |
| ibp_liq | Early-stage | Late-stage | 14 | 15 | 62 | 0.063 | 0.252 | ns |
| ibp_liq | Early-stage | Non-cancerous | 14 | 18 | 146 | 0.464 | 1.000 | ns |
| ibp_liq | Late-stage | Non-cancerous | 15 | 18 | 209 | 0.007 | 0.040 | * |

pairwise_wilcox_test(df,shbg_qae ~ sample_group, p.adjust.method = "holm")%>%
 kable() %>% kable_paper()

| .y. | group1 | group2 | n1 | n2 | statistic | p | p.adj | p.adj.signif |
| --- | --- | --- | --- | --- | --- | --- | --- | --- |
| shbg_qae | Benign | Early-stage | 15 | 16 | 118 | 0.953 | 1.000 | ns |
| shbg_qae | Benign | Late-stage | 15 | 16 | 88 | 0.216 | 0.864 | ns |
| shbg_qae | Benign | Non-cancerous | 15 | 16 | 129 | 0.740 | 1.000 | ns |
| shbg_qae | Early-stage | Late-stage | 16 | 16 | 84 | 0.102 | 0.510 | ns |
| shbg_qae | Early-stage | Non-cancerous | 16 | 16 | 141 | 0.642 | 1.000 | ns |
| shbg_qae | Late-stage | Non-cancerous | 16 | 16 | 175 | 0.080 | 0.478 | ns |

pairwise_wilcox_test(df,shbg_ldv ~ sample_group, p.adjust.method = "holm")%>% kable() %>% kable_paper()

| .y. | group1 | group2 | n1 | n2 | statistic | p | p.adj | p.adj.signif |
| --- | --- | --- | --- | --- | --- | --- | --- | --- |
| shbg_ldv | Benign | Early-stage | 8 | 11 | 33 | 0.395 | 0.790 | ns |
| shbg_ldv | Benign | Late-stage | 8 | 14 | 29 | 0.070 | 0.349 | ns |
| shbg_ldv | Benign | Non-cancerous | 8 | 11 | 16 | 0.020 | 0.122 | ns |
| shbg_ldv | Early-stage | Late-stage | 11 | 14 | 50 | 0.149 | 0.447 | ns |
| shbg_ldv | Early-stage | Non-cancerous | 11 | 11 | 35 | 0.101 | 0.404 | ns |
| shbg_ldv | Late-stage | Non-cancerous | 14 | 11 | 72 | 0.809 | 0.809 | ns |

pairwise_wilcox_test(df,timp1_gfq ~ sample_group, p.adjust.method = "holm")%>%
 kable() %>% kable_paper()

| .y. | group1 | group2 | n1 | n2 | statistic | p | p.adj | p.adj.signif |
| --- | --- | --- | --- | --- | --- | --- | --- | --- |
| timp1_gfq | Benign | Early-stage | 12 | 15 | 71 | 0.373 | 0.746 | ns |
| timp1_gfq | Benign | Late-stage | 12 | 13 | 37 | 0.026 | 0.128 | ns |
| timp1_gfq | Benign | Non-cancerous | 12 | 14 | 88 | 0.860 | 0.860 | ns |
| timp1_gfq | Early-stage | Late-stage | 15 | 13 | 60 | 0.088 | 0.354 | ns |
| timp1_gfq | Early-stage | Non-cancerous | 15 | 14 | 144 | 0.093 | 0.354 | ns |
| timp1_gfq | Late-stage | Non-cancerous | 13 | 14 | 147 | 0.006 | 0.034 | * |

pairwise_wilcox_test(df,timp1_see ~ sample_group, p.adjust.method = "holm")%>%
 kable() %>% kable_paper()

| .y. | group1 | group2 | n1 | n2 | statistic | p | p.adj | p.adj.signif |
| --- | --- | --- | --- | --- | --- | --- | --- | --- |
| timp1_see | Benign | Early-stage | 12 | 12 | 52 | 0.266 | 1.000 | ns |
| timp1_see | Benign | Late-stage | 12 | 10 | 53 | 0.674 | 1.000 | ns |
| timp1_see | Benign | Non-cancerous | 12 | 7 | 42 | 1.000 | 1.000 | ns |
| timp1_see | Early-stage | Late-stage | 12 | 10 | 64 | 0.821 | 1.000 | ns |
| timp1_see | Early-stage | Non-cancerous | 12 | 7 | 63 | 0.083 | 0.499 | ns |
| timp1_see | Late-stage | Non-cancerous | 10 | 7 | 43 | 0.475 | 1.000 | ns |

### Section 3

To evaluate candidate biomarkers with receiver operating characteristic (ROC) curves, the data was normalized to ensure that the concentrations of all peptides and proteins were on the same scale. Consequently, all the peptides had equal weight in multivariate logistic regressions. Each peptide was normalized to have a mean = 0 and standard deviation = 1.

#scale the columns
df2<-df %>% mutate(across(where(is.double),scale))

# relabel the groups
df2<-df2 %>% mutate(sample_group2=case_when(
 sample_group=="Late-stage" ~ "L",
 sample_group=="Early-stage" ~ "E",
 sample_group=="Benign" ~"B",
 sample_group=="Non-cancerous" ~ "NC")) %>% select(-sample_group) %>%
 rename(sample_group = sample_group2)

# select the grouping variable and some peptides
df2<-df2 %>% select(sample_group,ibp_leg,ibp_liq,timp1_gfq)

### Section 4

Logistic regression classifiers were used to determine the propensity scores for cancer stages from various combinations of biomarkers. The dependent variable was two cancer stages, for example, no cancer (NC) and late-stage cancer (L). The independent variables were univariate and multivariate combinations of peptides. The propensity scores are the probabilities of the case belonging to a cancer stage.

Using 0.5 as a propensity-score cutoff, scores above 0.5 were assigned 1, and scores below 0.5 were assigned 0. For example, if the logistic regression dependent variable used NC and L, then cases with propensity score above 0.5 would be assigned 1 and predicted to be L, and cases with propensity score below 0.5 would be assigned 0 and predicted to be NC.

So, at this point, we have the true disease classifications (e.g., NC and L), we have predicted values for NC and L (using the 0.5 cutoff) which are used to estimate sensitivity and specificity, and the propensity scores, which are used to calculate ROC curves and calculate areas under curves (AUC). The p-values for the AUC were the p-values of the whole model tests of the logistic regressions.

All this one done with one R function

classifierfun<-function(df_temp){
df_temp<- df_temp %>% tidyr::drop_na()

#the logistic model
model <- glm(labelz ~.,family=binomial(link='logit'),data=df_temp)


#This calculates the overall p.value for the logistic regression against null model

null.mod <- glm(labelz~1
 , family =binomial(link='logit')
 ,data=df_temp) # null model
lrt.p.value<-anova(null.mod, model, test = 'LRT')$`Pr(>Chi)`[2]
#-----------------------

# propensity scores
fitted.results <- predict(model,newdata=df_temp,type='response')

# 0 or 1 predictions using 0.5
fitted.results_0_1 <- ifelse(fitted.results > 0.5,1,0)

#THis finds the accuracy stats------------------------
acc_out<-caret::confusionMatrix(as.factor(fitted.results_0_1)
 ,reference=as.factor(df_temp$labelz),positive="1")
acc<- acc_out[[3]][1]
acc_low<- acc_out[[3]][3]
acc_up<- acc_out[[3]][4]

#this finds the se and sp stats----------------------------
dat_mat <- as.table(acc_out[[2]], nrow = 2, byrow = TRUE)
rval <- epi.tests(dat_mat, conf.level = 0.95)

se<-rval$detail$est[3]
se_low<-rval$detail$lower[3]
se_up<-rval$detail$upper[3]

sp<-rval$detail$est[4]
sp_low<-rval$detail$lower[4]
sp_up<-rval$detail$upper[4]

# ROC
roc_empirical <- ROCit::rocit(score = fitted.results, class = df_temp$labelz,
 negref = 0)
auc_ci<-ROCit::ciAUC(roc_empirical)

auc_low<-auc_ci[5]$lower
auc_up<-auc_ci[6]$upper
auc<-auc_ci[1]$AUC

#-------------------------------------------------

out_df<-data.frame(
 accuracy = c(acc_low,acc,acc_up),
 sensitivity = c(se_low,se,se_up),
 specificity= c(sp_low,sp, sp_up),
 AUC = c(auc_low,auc, auc_up),
 p_value = c(lrt.p.value,NA,NA)
)

rownames(out_df)<-c("lower bound","estimate","upper bound")

#replace NA with blank for looks in the output table
out_df<-out_df %>%
 mutate_at("p_value",round,4) %>%
 mutate(p_value=as.character(p_value)) %>%
 mutate_at("p_value",~replace_na(.,""))

return(out_df)

}

### Section 5

Accuracy, sensitivity, and specificity, and their 95% Delong confidence intervals (CI), for combinations of biomarkers, were calculated using a 0.5 cutoff based on the scores from the logistic regressions .

This section shows the results for NC and L only, but the same methods were used for all the other pairs of cancer stages.

**NC vs L**

**IBP2_LEG only**

classifierfun(df3 %>% dplyr::select(labelz,ibp_leg)) %>% kable(digits=4) %>% kable_classic(full_width=F)

|  | accuracy | sensitivity | specificity | AUC | p_value |
| --- | --- | --- | --- | --- | --- |
| lower bound | 0.4226 | 0.3857 | 0.2988 | 0.5233 | 0.0207 |
| estimate | 0.6207 | 0.6923 | 0.5625 | 0.7115 |  |
| upper bound | 0.7931 | 0.9091 | 0.8025 | 0.8998 |  |

**IBP2_LIQ only**

classifierfun(df3 %>% dplyr::select(labelz,ibp_liq)) %>% kable(digits=4) %>% kable_classic(full_width=F)

|  | accuracy | sensitivity | specificity | AUC | p_value |
| --- | --- | --- | --- | --- | --- |
| lower bound | 0.4817 | 0.5236 | 0.2659 | 0.6087 | 0.0019 |
| estimate | 0.6667 | 0.7778 | 0.5333 | 0.7741 |  |
| upper bound | 0.8204 | 0.9359 | 0.7873 | 0.9395 |  |

**TIMP1_GFQ only**

classifierfun(df3 %>% dplyr::select(labelz,timp1_gfq))%>% kable(digits=4) %>% kable_classic(full_width=F)

|  | accuracy | sensitivity | specificity | AUC | p_value |
| --- | --- | --- | --- | --- | --- |
| lower bound | 0.6192 | 0.6613 | 0.3857 | 0.6384 | 4e-04 |
| estimate | 0.8148 | 0.9286 | 0.6923 | 0.8077 |  |
| upper bound | 0.9370 | 0.9982 | 0.9091 | 0.9770 |  |

**Combination of IBP2_LEG and IBP2_LIQ**

classifierfun(df3 %>% dplyr::select(labelz,ibp_leg,ibp_liq))%>% kable(digits=4) %>% kable_classic(full_width=F)

|  | accuracy | sensitivity | specificity | AUC | p_value |
| --- | --- | --- | --- | --- | --- |
| lower bound | 0.4058 | 0.3857 | 0.2659 | 0.5147 | 0.0595 |
| estimate | 0.6071 | 0.6923 | 0.5333 | 0.7077 |  |
| upper bound | 0.7850 | 0.9091 | 0.7873 | 0.9007 |  |

**Combination of IBP2_LEG and TIMP1_GFQ**

classifierfun(df3 %>% dplyr::select(labelz,ibp_leg,timp1_gfq))%>% kable(digits=4) %>% kable_classic(full_width=F)

|  | accuracy | sensitivity | specificity | AUC | p_value |
| --- | --- | --- | --- | --- | --- |
| lower bound | 0.5635 | 0.6397 | 0.3158 | 0.6162 | 0.0028 |
| estimate | 0.7692 | 0.9231 | 0.6154 | 0.7929 |  |
| upper bound | 0.9103 | 0.9981 | 0.8614 | 0.9696 |  |

**Combination of IBP2_LIQ and TIMP1_GFQ**

classifierfun(df3 %>% dplyr::select(labelz,ibp_liq,timp1_gfq))%>% kable(digits=4) %>% kable_classic(full_width=F)

|  | accuracy | sensitivity | specificity | AUC | p_value |
| --- | --- | --- | --- | --- | --- |
| lower bound | 0.5774 | 0.6613 | 0.3158 | 0.6460 | 0.0017 |
| estimate | 0.7778 | 0.9286 | 0.6154 | 0.8132 |  |
| upper bound | 0.9138 | 0.9982 | 0.8614 | 0.9804 |  |

**Combination of IBP2_LEG, IBP2_LIQ and TIMP1_GFQ**

classifierfun(df3 %>% dplyr::select(labelz,ibp_liq,ibp_leg,timp1_gfq))%>% kable(digits=4) %>% kable_classic(full_width=F)

|  | accuracy | sensitivity | specificity | AUC | p_value |
| --- | --- | --- | --- | --- | --- |
| lower bound | 0.6513 | 0.7529 | 0.3857 | 0.5923 | 0.0074 |
| estimate | 0.8462 | 1.0000 | 0.6923 | 0.7751 |  |
| upper bound | 0.9564 | 1.0000 | 0.9091 | 0.9580 |  |

#

# Appendix

This appendix contains code for all the data analysis. Some of the Figures in the manuscript were created with other software, and are not included in this document.

# NC vs L

#make the labelz

df3 <- df2 %>% filter(sample_group=="NC" | sample_group=="L") %>%
 mutate(labelz=case_when(sample_group=="NC" ~ 0L,TRUE~1L,)) %>%
 dplyr::select(-sample_group)

## IBP2_LEG only

classifierfun(df3 %>% dplyr::select(labelz,ibp_leg)) %>% kable(digits=4) %>% kable_classic(full_width=F)

|  | accuracy | sensitivity | specificity | AUC | p_value |
| --- | --- | --- | --- | --- | --- |
| lower bound | 0.4226 | 0.3857 | 0.2988 | 0.5233 | 0.0207 |
| estimate | 0.6207 | 0.6923 | 0.5625 | 0.7115 |  |
| upper bound | 0.7931 | 0.9091 | 0.8025 | 0.8998 |  |

## IBP2_LIQ only

classifierfun(df3 %>% dplyr::select(labelz,ibp_liq)) %>% kable(digits=4) %>% kable_classic(full_width=F)

|  | accuracy | sensitivity | specificity | AUC | p_value |
| --- | --- | --- | --- | --- | --- |
| lower bound | 0.4817 | 0.5236 | 0.2659 | 0.6087 | 0.0019 |
| estimate | 0.6667 | 0.7778 | 0.5333 | 0.7741 |  |
| upper bound | 0.8204 | 0.9359 | 0.7873 | 0.9395 |  |

## TIMP1_GFQ only

classifierfun(df3 %>% dplyr::select(labelz,timp1_gfq))%>% kable(digits=4) %>% kable_classic(full_width=F)

|  | accuracy | sensitivity | specificity | AUC | p_value |
| --- | --- | --- | --- | --- | --- |
| lower bound | 0.6192 | 0.6613 | 0.3857 | 0.6384 | 4e-04 |
| estimate | 0.8148 | 0.9286 | 0.6923 | 0.8077 |  |
| upper bound | 0.9370 | 0.9982 | 0.9091 | 0.9770 |  |

## Combination of IBP2_LEG and IBP2_LIQ

classifierfun(df3 %>% dplyr::select(labelz,ibp_leg,ibp_liq))%>% kable(digits=4) %>% kable_classic(full_width=F)

|  | accuracy | sensitivity | specificity | AUC | p_value |
| --- | --- | --- | --- | --- | --- |
| lower bound | 0.4058 | 0.3857 | 0.2659 | 0.5147 | 0.0595 |
| estimate | 0.6071 | 0.6923 | 0.5333 | 0.7077 |  |
| upper bound | 0.7850 | 0.9091 | 0.7873 | 0.9007 |  |

## Combination of IBP2_LEG and TIMP1_GFQ

classifierfun(df3 %>% dplyr::select(labelz,ibp_leg,timp1_gfq))%>% kable(digits=4) %>% kable_classic(full_width=F)

|  | accuracy | sensitivity | specificity | AUC | p_value |
| --- | --- | --- | --- | --- | --- |
| lower bound | 0.5635 | 0.6397 | 0.3158 | 0.6162 | 0.0028 |
| estimate | 0.7692 | 0.9231 | 0.6154 | 0.7929 |  |
| upper bound | 0.9103 | 0.9981 | 0.8614 | 0.9696 |  |

## Combination of IBP2_LIQ and TIMP1_GFQ

classifierfun(df3 %>% dplyr::select(labelz,ibp_liq,timp1_gfq))%>% kable(digits=4) %>% kable_classic(full_width=F)

|  | accuracy | sensitivity | specificity | AUC | p_value |
| --- | --- | --- | --- | --- | --- |
| lower bound | 0.5774 | 0.6613 | 0.3158 | 0.6460 | 0.0017 |
| estimate | 0.7778 | 0.9286 | 0.6154 | 0.8132 |  |
| upper bound | 0.9138 | 0.9982 | 0.8614 | 0.9804 |  |

## Combination of IBP2_LEG, IBP2_LIQ and TIMP1_GFQ

classifierfun(df3 %>% dplyr::select(labelz,ibp_liq,ibp_leg,timp1_gfq))%>% kable(digits=4) %>% kable_classic(full_width=F)

|  | accuracy | sensitivity | specificity | AUC | p_value |
| --- | --- | --- | --- | --- | --- |
| lower bound | 0.6513 | 0.7529 | 0.3857 | 0.5923 | 0.0074 |
| estimate | 0.8462 | 1.0000 | 0.6923 | 0.7751 |  |
| upper bound | 0.9564 | 1.0000 | 0.9091 | 0.9580 |  |

# B vs L

#make the labelz

df3 <- df2 %>% filter(sample_group=="B" | sample_group=="L") %>%
 mutate(labelz=case_when(sample_group=="B" ~ 0L,TRUE~1L,)) %>%
 dplyr::select(-sample_group)

## IBP2_LEG only

classifierfun(df3 %>% dplyr::select(labelz,ibp_leg)) %>% kable(digits=4) %>% kable_classic(full_width=F)

|  | accuracy | sensitivity | specificity | AUC | p_value |
| --- | --- | --- | --- | --- | --- |
| lower bound | 0.4567 | 0.3158 | 0.4134 | 0.5233 | 0.0154 |
| estimate | 0.6552 | 0.6154 | 0.6875 | 0.7115 |  |
| upper bound | 0.8206 | 0.8614 | 0.8898 | 0.8998 |  |

## IBP2_LIQ only

classifierfun(df3 %>% dplyr::select(labelz,ibp_liq)) %>% kable(digits=4) %>% kable_classic(full_width=F)

|  | accuracy | sensitivity | specificity | AUC | p_value |
| --- | --- | --- | --- | --- | --- |
| lower bound | 0.4817 | 0.5236 | 0.2659 | 0.6087 | 0.0019 |
| estimate | 0.6667 | 0.7778 | 0.5333 | 0.7741 |  |
| upper bound | 0.8204 | 0.9359 | 0.7873 | 0.9395 |  |

## TIMP1_GFQ only

classifierfun(df3 %>% dplyr::select(labelz,timp1_gfq))%>% kable(digits=4) %>% kable_classic(full_width=F)

|  | accuracy | sensitivity | specificity | AUC | p_value |
| --- | --- | --- | --- | --- | --- |
| lower bound | 0.5061 | 0.5159 | 0.3158 | 0.5733 | 0.0356 |
| estimate | 0.7200 | 0.8333 | 0.6154 | 0.7628 |  |
| upper bound | 0.8793 | 0.9791 | 0.8614 | 0.9523 |  |

## Combination of IBP2_LEG and IBP2_LIQ

classifierfun(df3 %>% dplyr::select(labelz,ibp_leg,ibp_liq))%>% kable(digits=4) %>% kable_classic(full_width=F)

|  | accuracy | sensitivity | specificity | AUC | p_value |
| --- | --- | --- | --- | --- | --- |
| lower bound | 0.5513 | 0.5455 | 0.3838 | 0.5732 | 0.0178 |
| estimate | 0.7500 | 0.8462 | 0.6667 | 0.7538 |  |
| upper bound | 0.8931 | 0.9808 | 0.8818 | 0.9345 |  |

## Combination of IBP2_LEG and TIMP1_GFQ

classifierfun(df3 %>% dplyr::select(labelz,ibp_leg,timp1_gfq))%>% kable(digits=4) %>% kable_classic(full_width=F)

|  | accuracy | sensitivity | specificity | AUC | p_value |
| --- | --- | --- | --- | --- | --- |
| lower bound | 0.4066 | 0.2120 | 0.3857 | 0.5137 | 0.0871 |
| estimate | 0.6364 | 0.5556 | 0.6923 | 0.7265 |  |
| upper bound | 0.8280 | 0.8630 | 0.9091 | 0.9393 |  |

## Combination of IBP2_LIQ and TIMP1_GFQ

classifierfun(df3 %>% dplyr::select(labelz,ibp_liq,timp1_gfq))%>% kable(digits=4) %>% kable_classic(full_width=F)

|  | accuracy | sensitivity | specificity | AUC | p_value |
| --- | --- | --- | --- | --- | --- |
| lower bound | 0.4252 | 0.3489 | 0.3158 | 0.5481 | 0.0257 |
| estimate | 0.6400 | 0.6667 | 0.6154 | 0.7436 |  |
| upper bound | 0.8203 | 0.9008 | 0.8614 | 0.9391 |  |

## Combination of IBP2_LEG, IBP2_LIQ and TIMP1_GFQ

classifierfun(df3 %>% dplyr::select(labelz,ibp_liq,ibp_leg,timp1_gfq))%>% kable(digits=4) %>% kable_classic(full_width=F)

|  | accuracy | sensitivity | specificity | AUC | p_value |
| --- | --- | --- | --- | --- | --- |
| lower bound | 0.4513 | 0.2993 | 0.3857 | 0.4804 | 0.1458 |
| estimate | 0.6818 | 0.6667 | 0.6923 | 0.7009 |  |
| upper bound | 0.8614 | 0.9251 | 0.9091 | 0.9213 |  |

# NC vs B

#make the labelz

df3 <- df2 %>% filter(sample_group=="B" | sample_group=="NC") %>%
 mutate(labelz=case_when(sample_group=="B" ~ 0L,TRUE~1L,)) %>%
 dplyr::select(-sample_group)

## IBP2_LEG only

classifierfun(df3 %>% dplyr::select(labelz,ibp_leg)) %>% kable(digits=4) %>% kable_classic(full_width=F)

|  | accuracy | sensitivity | specificity | AUC | p_value |
| --- | --- | --- | --- | --- | --- |
| lower bound | 0.3337 | 0.3158 | 0.1922 | 0.3131 | 0.8264 |
| estimate | 0.5385 | 0.6154 | 0.4615 | 0.5385 |  |
| upper bound | 0.7341 | 0.8614 | 0.7487 | 0.7638 |  |

## IBP2_LIQ only

classifierfun(df3 %>% dplyr::select(labelz,ibp_liq)) %>% kable(digits=4) %>% kable_classic(full_width=F)

|  | accuracy | sensitivity | specificity | AUC | p_value |
| --- | --- | --- | --- | --- | --- |
| lower bound | 0.3292 | 0.5236 | 0.0641 | 0.3088 | 1 |
| estimate | 0.5000 | 0.7778 | 0.2222 | 0.5000 |  |
| upper bound | 0.6708 | 0.9359 | 0.4764 | 0.6912 |  |

## TIMP1_GFQ only

classifierfun(df3 %>% dplyr::select(labelz,timp1_gfq))%>% kable(digits=4) %>% kable_classic(full_width=F)

|  | accuracy | sensitivity | specificity | AUC | p_value |
| --- | --- | --- | --- | --- | --- |
| lower bound | 0.4057 | 0.0992 | 0.5719 | 0.2976 | 0.2924 |
| estimate | 0.6154 | 0.3333 | 0.8571 | 0.5238 |  |
| upper bound | 0.7977 | 0.6511 | 0.9822 | 0.7501 |  |

## Combination of IBP2_LEG and IBP2_LIQ

classifierfun(df3 %>% dplyr::select(labelz,ibp_leg,ibp_liq))%>% kable(digits=4) %>% kable_classic(full_width=F)

|  | accuracy | sensitivity | specificity | AUC | p_value |
| --- | --- | --- | --- | --- | --- |
| lower bound | 0.2993 | 0.1922 | 0.2513 | 0.3193 | 0.8176 |
| estimate | 0.5000 | 0.4615 | 0.5385 | 0.5444 |  |
| upper bound | 0.7007 | 0.7487 | 0.8078 | 0.7695 |  |

## Combination of IBP2_LEG and TIMP1_GFQ

classifierfun(df3 %>% dplyr::select(labelz,ibp_leg,timp1_gfq))%>% kable(digits=4) %>% kable_classic(full_width=F)

|  | accuracy | sensitivity | specificity | AUC | p_value |
| --- | --- | --- | --- | --- | --- |
| lower bound | 0.4066 | 0.0281 | 0.6397 | 0.4063 | 0.2291 |
| estimate | 0.6364 | 0.2222 | 0.9231 | 0.6410 |  |
| upper bound | 0.8280 | 0.6001 | 0.9981 | 0.8758 |  |

## Combination of IBP2_LIQ and TIMP1_GFQ

classifierfun(df3 %>% dplyr::select(labelz,ibp_liq,timp1_gfq))%>% kable(digits=4) %>% kable_classic(full_width=F)

|  | accuracy | sensitivity | specificity | AUC | p_value |
| --- | --- | --- | --- | --- | --- |
| lower bound | 0.3337 | 0.0549 | 0.4920 | 0.3226 | 0.5511 |
| estimate | 0.5385 | 0.2500 | 0.7857 | 0.5476 |  |
| upper bound | 0.7341 | 0.5719 | 0.9534 | 0.7727 |  |

## Combination of IBP2_LEG, IBP2_LIQ and TIMP1_GFQ

classifierfun(df3 %>% dplyr::select(labelz,ibp_liq,ibp_leg,timp1_gfq))%>% kable(digits=4) %>% kable_classic(full_width=F)

|  | accuracy | sensitivity | specificity | AUC | p_value |
| --- | --- | --- | --- | --- | --- |
| lower bound | 0.4066 | 0.0749 | 0.5455 | 0.5137 | 0.294 |
| estimate | 0.6364 | 0.3333 | 0.8462 | 0.7265 |  |
| upper bound | 0.8280 | 0.7007 | 0.9808 | 0.9393 |  |

# NC vs E+L

#make the labelz

df3 <- df2 %>% filter(sample_group=="E" | sample_group=="L"| sample_group=="NC") %>%
 mutate(labelz=case_when(sample_group=="NC" ~ 0L,TRUE~1L,)) %>%
 dplyr::select(-sample_group)

## IBP2_LEG only

classifierfun(df3 %>% dplyr::select(labelz,ibp_leg)) %>% kable(digits=4) %>% kable_classic(full_width=F)

|  | accuracy | sensitivity | specificity | AUC | p_value |
| --- | --- | --- | --- | --- | --- |
| lower bound | 0.5569 | 0.0000 | 0.8911 | 0.4063 | 0.1446 |
| estimate | 0.7111 | 0.0000 | 1.0000 | 0.5865 |  |
| upper bound | 0.8363 | 0.2471 | 1.0000 | 0.7668 |  |

## IBP2_LIQ only

classifierfun(df3 %>% dplyr::select(labelz,ibp_liq)) %>% kable(digits=4) %>% kable_classic(full_width=F)

|  | accuracy | sensitivity | specificity | AUC | p_value |
| --- | --- | --- | --- | --- | --- |
| lower bound | 0.5069 | 0.1730 | 0.6423 | 0.5268 | 0.0127 |
| estimate | 0.6596 | 0.3889 | 0.8276 | 0.6801 |  |
| upper bound | 0.7914 | 0.6425 | 0.9415 | 0.8333 |  |

## TIMP1_GFQ only

classifierfun(df3 %>% dplyr::select(labelz,timp1_gfq))%>% kable(digits=4) %>% kable_classic(full_width=F)

|  | accuracy | sensitivity | specificity | AUC | p_value |
| --- | --- | --- | --- | --- | --- |
| lower bound | 0.5045 | 0.0839 | 0.6733 | 0.5919 | 0.0022 |
| estimate | 0.6667 | 0.2857 | 0.8571 | 0.7423 |  |
| upper bound | 0.8043 | 0.5810 | 0.9597 | 0.8928 |  |

## Combination of IBP2_LEG and IBP2_LIQ

classifierfun(df3 %>% dplyr::select(labelz,ibp_leg,ibp_liq))%>% kable(digits=4) %>% kable_classic(full_width=F)

|  | accuracy | sensitivity | specificity | AUC | p_value |
| --- | --- | --- | --- | --- | --- |
| lower bound | 0.5291 | 0.0000 | 0.8806 | 0.4795 | 0.225 |
| estimate | 0.6905 | 0.0000 | 1.0000 | 0.6525 |  |
| upper bound | 0.8238 | 0.2471 | 1.0000 | 0.8256 |  |

## Combination of IBP2_LEG and TIMP1_GFQ

classifierfun(df3 %>% dplyr::select(labelz,ibp_leg,timp1_gfq))%>% kable(digits=4) %>% kable_classic(full_width=F)

|  | accuracy | sensitivity | specificity | AUC | p_value |
| --- | --- | --- | --- | --- | --- |
| lower bound | 0.4941 | 0.0504 | 0.6733 | 0.5778 | 0.0154 |
| estimate | 0.6585 | 0.2308 | 0.8571 | 0.7335 |  |
| upper bound | 0.7992 | 0.5381 | 0.9597 | 0.8893 |  |

## Combination of IBP2_LIQ and TIMP1_GFQ

classifierfun(df3 %>% dplyr::select(labelz,ibp_liq,timp1_gfq))%>% kable(digits=4) %>% kable_classic(full_width=F)

|  | accuracy | sensitivity | specificity | AUC | p_value |
| --- | --- | --- | --- | --- | --- |
| lower bound | 0.4580 | 0.1276 | 0.5635 | 0.6057 | 0.0058 |
| estimate | 0.6250 | 0.3571 | 0.7692 | 0.7555 |  |
| upper bound | 0.7727 | 0.6486 | 0.9103 | 0.9053 |  |

## Combination of IBP2_LEG, IBP2_LIQ and TIMP1_GFQ

classifierfun(df3 %>% dplyr::select(labelz,ibp_liq,ibp_leg,timp1_gfq))%>% kable(digits=4) %>% kable_classic(full_width=F)

|  | accuracy | sensitivity | specificity | AUC | p_value |
| --- | --- | --- | --- | --- | --- |
| lower bound | 0.5243 | 0.1386 | 0.6513 | 0.6365 | 0.0155 |
| estimate | 0.6923 | 0.3846 | 0.8462 | 0.7811 |  |
| upper bound | 0.8298 | 0.6842 | 0.9564 | 0.9256 |  |

# B vs E+L

#make the labelz

df3 <- df2 %>% filter(sample_group=="B" | sample_group=="L"| sample_group=="E") %>%
 mutate(labelz=case_when(sample_group=="B" ~ 0L,TRUE~1L,)) %>%
 dplyr::select(-sample_group)

## IBP2_LEG only

classifierfun(df3 %>% dplyr::select(labelz,ibp_leg)) %>% kable(digits=4) %>% kable_classic(full_width=F)

|  | accuracy | sensitivity | specificity | AUC | p_value |
| --- | --- | --- | --- | --- | --- |
| lower bound | 0.5569 | 0.0000 | 0.8911 | 0.4227 | 0.1062 |
| estimate | 0.7111 | 0.0000 | 1.0000 | 0.6010 |  |
| upper bound | 0.8363 | 0.2471 | 1.0000 | 0.7792 |  |

## IBP2_LIQ only

classifierfun(df3 %>% dplyr::select(labelz,ibp_liq)) %>% kable(digits=4) %>% kable_classic(full_width=F)

|  | accuracy | sensitivity | specificity | AUC | p_value |
| --- | --- | --- | --- | --- | --- |
| lower bound | 0.5069 | 0.1730 | 0.6423 | 0.5268 | 0.0127 |
| estimate | 0.6596 | 0.3889 | 0.8276 | 0.6801 |  |
| upper bound | 0.7914 | 0.6425 | 0.9415 | 0.8333 |  |

## TIMP1_GFQ only

classifierfun(df3 %>% dplyr::select(labelz,timp1_gfq))%>% kable(digits=4) %>% kable_classic(full_width=F)

|  | accuracy | sensitivity | specificity | AUC | p_value |
| --- | --- | --- | --- | --- | --- |
| lower bound | 0.5347 | 0.0000 | 0.8766 | 0.5055 | 0.1196 |
| estimate | 0.7000 | 0.0000 | 1.0000 | 0.6786 |  |
| upper bound | 0.8344 | 0.2646 | 1.0000 | 0.8517 |  |

## Combination of IBP2_LEG and IBP2_LIQ

classifierfun(df3 %>% dplyr::select(labelz,ibp_leg,ibp_liq))%>% kable(digits=4) %>% kable_classic(full_width=F)

|  | accuracy | sensitivity | specificity | AUC | p_value |
| --- | --- | --- | --- | --- | --- |
| lower bound | 0.5045 | 0.0000 | 0.8224 | 0.4732 | 0.1157 |
| estimate | 0.6667 | 0.0000 | 0.9655 | 0.6472 |  |
| upper bound | 0.8043 | 0.2471 | 0.9991 | 0.8212 |  |

## Combination of IBP2_LEG and TIMP1_GFQ

classifierfun(df3 %>% dplyr::select(labelz,ibp_leg,timp1_gfq))%>% kable(digits=4) %>% kable_classic(full_width=F)

|  | accuracy | sensitivity | specificity | AUC | p_value |
| --- | --- | --- | --- | --- | --- |
| lower bound | 0.5880 | 0.0000 | 0.8766 | 0.3920 | 0.3208 |
| estimate | 0.7568 | 0.0000 | 1.0000 | 0.5992 |  |
| upper bound | 0.8823 | 0.3363 | 1.0000 | 0.8064 |  |

## Combination of IBP2_LIQ and TIMP1_GFQ

classifierfun(df3 %>% dplyr::select(labelz,ibp_liq,timp1_gfq))%>% kable(digits=4) %>% kable_classic(full_width=F)

|  | accuracy | sensitivity | specificity | AUC | p_value |
| --- | --- | --- | --- | --- | --- |
| lower bound | 0.5410 | 0.0209 | 0.8036 | 0.4574 | 0.1235 |
| estimate | 0.7105 | 0.1667 | 0.9615 | 0.6410 |  |
| upper bound | 0.8458 | 0.4841 | 0.9990 | 0.8246 |  |

## Combination of IBP2_LEG, IBP2_LIQ and TIMP1_GFQ

classifierfun(df3 %>% dplyr::select(labelz,ibp_liq,ibp_leg,timp1_gfq))%>% kable(digits=4) %>% kable_classic(full_width=F)

|  | accuracy | sensitivity | specificity | AUC | p_value |
| --- | --- | --- | --- | --- | --- |
| lower bound | 0.5674 | 0.0000 | 0.8677 | 0.4036 | 0.3996 |
| estimate | 0.7429 | 0.0000 | 1.0000 | 0.6111 |  |
| upper bound | 0.8751 | 0.3363 | 1.0000 | 0.8186 |  |

# NC+B vs E+L

#make the labelz

df3 <- df2 %>%
 mutate(labelz=case_when((sample_group=="B" |sample_group=="NC") ~ 0L
 ,(sample_group=="E" |sample_group=="L")~1L,)) %>%
 dplyr::select(-sample_group)

## IBP2_LEG only

classifierfun(df3 %>% dplyr::select(labelz,ibp_leg)) %>% kable(digits=4) %>% kable_classic(full_width=F)

|  | accuracy | sensitivity | specificity | AUC | p_value |
| --- | --- | --- | --- | --- | --- |
| lower bound | 0.3822 | 0.2335 | 0.4064 | 0.4473 | 0.0461 |
| estimate | 0.5172 | 0.4231 | 0.5938 | 0.5938 |  |
| upper bound | 0.6505 | 0.6308 | 0.7630 | 0.7402 |  |

## IBP2_LIQ only

classifierfun(df3 %>% dplyr::select(labelz,ibp_liq)) %>% kable(digits=4) %>% kable_classic(full_width=F)

|  | accuracy | sensitivity | specificity | AUC | p_value |
| --- | --- | --- | --- | --- | --- |
| lower bound | 0.4710 | 0.6085 | 0.2069 | 0.5475 | 0.002 |
| estimate | 0.6000 | 0.7778 | 0.3793 | 0.6801 |  |
| upper bound | 0.7196 | 0.8988 | 0.5774 | 0.8127 |  |

## TIMP1_GFQ only

classifierfun(df3 %>% dplyr::select(labelz,timp1_gfq))%>% kable(digits=4) %>% kable_classic(full_width=F)

|  | accuracy | sensitivity | specificity | AUC | p_value |
| --- | --- | --- | --- | --- | --- |
| lower bound | 0.5062 | 0.5221 | 0.3718 | 0.5757 | 0.0058 |
| estimate | 0.6481 | 0.7308 | 0.5714 | 0.7129 |  |
| upper bound | 0.7732 | 0.8843 | 0.7554 | 0.8501 |  |

## Combination of IBP2_LEG and IBP2_LIQ

classifierfun(df3 %>% dplyr::select(labelz,ibp_leg,ibp_liq))%>% kable(digits=4) %>% kable_classic(full_width=F)

|  | accuracy | sensitivity | specificity | AUC | p_value |
| --- | --- | --- | --- | --- | --- |
| lower bound | 0.5142 | 0.5635 | 0.3569 | 0.4683 | 0.0577 |
| estimate | 0.6545 | 0.7692 | 0.5517 | 0.6167 |  |
| upper bound | 0.7776 | 0.9103 | 0.7355 | 0.7651 |  |

## Combination of IBP2_LEG and TIMP1_GFQ

classifierfun(df3 %>% dplyr::select(labelz,ibp_leg,timp1_gfq))%>% kable(digits=4) %>% kable_classic(full_width=F)

|  | accuracy | sensitivity | specificity | AUC | p_value |
| --- | --- | --- | --- | --- | --- |
| lower bound | 0.4919 | 0.3635 | 0.4765 | 0.5270 | 0.0583 |
| estimate | 0.6400 | 0.5909 | 0.6786 | 0.6753 |  |
| upper bound | 0.7708 | 0.7929 | 0.8412 | 0.8236 |  |

## Combination of IBP2_LIQ and TIMP1_GFQ

classifierfun(df3 %>% dplyr::select(labelz,ibp_liq,timp1_gfq))%>% kable(digits=4) %>% kable_classic(full_width=F)

|  | accuracy | sensitivity | specificity | AUC | p_value |
| --- | --- | --- | --- | --- | --- |
| lower bound | 0.4896 | 0.6065 | 0.2659 | 0.5621 | 0.0105 |
| estimate | 0.6346 | 0.8077 | 0.4615 | 0.7041 |  |
| upper bound | 0.7638 | 0.9345 | 0.6663 | 0.8462 |  |

## Combination of IBP2_LEG, IBP2_LIQ and TIMP1_GFQ

classifierfun(df3 %>% dplyr::select(labelz,ibp_liq,ibp_leg,timp1_gfq))%>% kable(digits=4) %>% kable_classic(full_width=F)

|  | accuracy | sensitivity | specificity | AUC | p_value |
| --- | --- | --- | --- | --- | --- |
| lower bound | 0.4527 | 0.5463 | 0.2659 | 0.5212 | 0.0808 |
| estimate | 0.6042 | 0.7727 | 0.4615 | 0.6731 |  |
| upper bound | 0.7423 | 0.9218 | 0.6663 | 0.8249 |  |

**Note**

This section of the Quarto document contains code that outputs files of plots for publication. The plots do not appear in the rendered document.

# NC vs L

#make the labelz

df3 <- df2 %>% filter(sample_group=="NC" | sample_group=="L") %>%
 mutate(labelz=case_when(sample_group=="NC" ~ 0L,TRUE~1L,)) %>%
 dplyr::select(-sample_group)

ibp_leg<-classifierfun(df3 %>% dplyr::select(labelz,ibp_leg))

ibp_liq<-classifierfun(df3 %>% dplyr::select(labelz,ibp_liq))

ca125<-classifierfun(df3 %>% dplyr::select(labelz,ca125))

ibp_leg.ibp_liq<-classifierfun(df3 %>% dplyr::select(labelz,ibp_leg,ibp_liq))

ibp_leg.ca125<-classifierfun(df3 %>% dplyr::select(labelz,ibp_leg,ca125))

ibp_liq.ca125<-classifierfun(df3 %>% dplyr::select(labelz,ibp_liq,ca125))

ibp_leg.ibp_liq.ca125<-classifierfun(df3 %>% dplyr::select(labelz,ibp_leg,ibp_liq,ca125))

foo<-bind_rows(
bind_cols(Biomarkers=rep("ca125",nrow(ca125)),ca125),
bind_cols(Biomarkers=rep("ibp_leg",nrow(ibp_leg)),ibp_leg),
bind_cols(Biomarkers=rep("ibp_liq",nrow(ibp_liq)),ibp_liq),
bind_cols(Biomarkers=rep("ibp_leg.ibp_liq",nrow(ibp_leg.ibp_liq)),ibp_leg.ibp_liq)
)

tiff("p1.tiff", units="in", width=5, height=5, res=600)

ggplot(foo,aes(d=df_temp.labelz,m=fitted.results,color=Biomarkers))+
 geom_roc(n.cuts=0
 ,show.legend = FALSE) + ####turn off or on legend
 style_roc(theme = theme_bw
 , xlab = "1 - Specificity"
 , ylab="Sensitivity") +
 scale_color_discrete(breaks=c('ca125','ibp_leg','ibp_liq',
 'ibp_leg.ibp_liq'),
 labels=c('CA125 (AUC: 0.00)'
 ,'IBP2 LEG (AUC: 0.021)'
 ,'IBP2 LIQ (AUC: 0.002)'
 ,'IBP2 LEG + IBP2 LIQ (AUC: 0.06)'))+
 ggtitle("NC vs late-stage")#+
 #theme(legend.justification = c(1, 0), legend.position = c(1, .5))
dev.off()

png
 2

foo<-bind_rows(
bind_cols(Biomarkers=rep("ca125",nrow(ca125)),ca125),
bind_cols(Biomarkers=rep("ibp_leg",nrow(ibp_leg)),ibp_leg),
bind_cols(Biomarkers=rep("ibp_liq",nrow(ibp_liq)),ibp_liq),
bind_cols(Biomarkers=rep("ibp_leg.ibp_liq",nrow(ibp_leg.ibp_liq)),ibp_leg.ibp_liq)
)

tiff("p2.tiff", units="in", width=5, height=5, res=600)

ggplot(foo,aes(d=df_temp.labelz,m=fitted.results,color=Biomarkers))+
 geom_roc(n.cuts=0
 ,show.legend = TRUE) +
 style_roc(theme = theme_bw
 , xlab = "1 - Specificity"
 , ylab="Sensitivity") +
 scale_color_discrete(breaks=c('ca125','ibp_leg','ibp_liq',
 'ibp_leg.ibp_liq'),
 labels=c('CA125 (AUC: 1.00)'
 ,'IBP2 LEG (AUC: 0.712)'
 ,'IBP2 LIQ (AUC: 0.774)'
 ,'IBP2 LEG + IBP2 LIQ (AUC: 0.701)'))+
 ggtitle("NC vs late-stage")#+
 #theme(legend.justification = c(1, 0), legend.position = c(1, .5))
dev.off()

png
 2

foo<-bind_rows(
bind_cols(Biomarkers=rep("ca125",nrow(ca125)),ca125),
bind_cols(Biomarkers=rep("ibp_leg.ca125",nrow(ibp_leg.ca125)),ibp_leg.ca125),
bind_cols(Biomarkers=rep("ibp_liq.ca125",nrow(ibp_liq.ca125)),ibp_liq.ca125)
)

tiff("p3.tiff", units="in", width=5, height=5, res=600)

ggplot(foo,aes(d=df_temp.labelz,m=fitted.results,color=Biomarkers))+
 geom_roc(n.cuts=0
 ,show.legend = FALSE) + ####turn off or on legend
 style_roc(theme = theme_bw
 , xlab = "1 - Specificity"
 , ylab="Sensitivity") +
 scale_color_discrete(breaks=c('ca125','ibp_leg.ca125','ibp_liq.ca125'
 ),
 labels=c('CA125 (AUC: 0.00)'
 ,'IBP2 LEG + CA125 (AUC: 0.00)'
 ,'IBP2 LIQ + CA125 (AUC: 0.00)'))+
 ggtitle("Late-stage vs late-stage")#+
 #theme(legend.justification = c(1, 0), legend.position = c(1, .5))
dev.off()

png
 2

foo<-bind_rows(
bind_cols(Biomarkers=rep("ca125",nrow(ca125)),ca125),
bind_cols(Biomarkers=rep("ibp_leg.ca125",nrow(ibp_leg.ca125)),ibp_leg.ca125),
bind_cols(Biomarkers=rep("ibp_liq.ca125",nrow(ibp_liq.ca125)),ibp_liq.ca125)
)

tiff("p4.tiff", units="in", width=5, height=5, res=600)

ggplot(foo,aes(d=df_temp.labelz,m=fitted.results,color=Biomarkers))+
 geom_roc(n.cuts=0
 ,show.legend = TRUE) + ####turn off or on legend
 style_roc(theme = theme_bw
 , xlab = "1 - Specificity"
 , ylab="Sensitivity") +
 scale_color_discrete(breaks=c('ca125','ibp_leg.ca125','ibp_liq.ca125'
 ),
 labels=c('CA125 (AUC: 1.00)'
 ,'IBP2 LEG + CA125 (AUC: 1.00)'
 ,'IBP2 LIQ + CA125 (AUC: 1.00)'))+
 ggtitle("Late-stage vs late-stage")#+
 #theme(legend.justification = c(1, 0), legend.position = c(1, .5))
dev.off()

png
 2

foo<-bind_rows(
bind_cols(Biomarkers=rep("ca125",nrow(ca125)),ca125),
bind_cols(Biomarkers=rep("ibp_leg.ibp_liq.ca125"
 ,nrow(ibp_leg.ibp_liq.ca125))
 ,ibp_leg.ibp_liq.ca125))

tiff("p5.tiff", units="in", width=5, height=5, res=600)

ggplot(foo,aes(d=df_temp.labelz,m=fitted.results,color=Biomarkers))+
 geom_roc(n.cuts=0
 ,show.legend = FALSE) + ####turn off or on legend
 style_roc(theme = theme_bw
 , xlab = "1 - Specificity"
 , ylab="Sensitivity") +
 scale_color_discrete(breaks=c('ca125','ibp.leg.ibp_liq.ca125'
 ),
 labels=c('CA125 (AUC: 0.00)'
 ,'IBP2 LEG +IBP2 LIQ + CA125 (AUC: 0.00 )'))+
 ggtitle("Late-stage vs late-stage")#+
 #theme(legend.justification = c(1, 0), legend.position = c(1, .5))
dev.off()

png
 2

foo<-bind_rows(
bind_cols(Biomarkers=rep("ca125",nrow(ca125)),ca125),
bind_cols(Biomarkers=rep("ibp_leg.ibp_liq.ca125"
 ,nrow(ibp_leg.ibp_liq.ca125))
 ,ibp_leg.ibp_liq.ca125))

tiff("p6.tiff", units="in", width=5, height=5, res=600)

ggplot(foo,aes(d=df_temp.labelz,m=fitted.results,color=Biomarkers))+
 geom_roc(n.cuts=0
 ,show.legend = TRUE) + ####turn off or on legend
 style_roc(theme = theme_bw
 , xlab = "1 - Specificity"
 , ylab="Sensitivity") +
 scale_color_discrete(breaks=c('ca125','ibp_leg.ibp_liq.ca125'
 ),
 labels=c('CA125 (AUC: 1.00)'
 ,'IBP2 LEG +IBP2 LIQ + CA125 (AUC: 1.00)'))+
 ggtitle("Late-stage vs late-stage")#+
 #theme(legend.justification = c(1, 0), legend.position = c(1, .5))
dev.off()

png
 2

#make the labelz

df3 <- df2 %>% filter(sample_group=="B" | sample_group=="L") %>%
 mutate(labelz=case_when(sample_group=="B" ~ 0L,TRUE~1L,)) %>%
 dplyr::select(-sample_group)

ibp_leg<-classifierfun(df3 %>% dplyr::select(labelz,ibp_leg))

ibp_liq<-classifierfun(df3 %>% dplyr::select(labelz,ibp_liq))

ca125<-classifierfun(df3 %>% dplyr::select(labelz,ca125))

ibp_leg.ibp_liq<-classifierfun(df3 %>% dplyr::select(labelz,ibp_leg,ibp_liq))

ibp_leg.ca125<-classifierfun(df3 %>% dplyr::select(labelz,ibp_leg,ca125))

ibp_liq.ca125<-classifierfun(df3 %>% dplyr::select(labelz,ibp_liq,ca125))

ibp_leg.ibp_liq.ca125<-classifierfun(df3 %>% dplyr::select(labelz,ibp_leg,ibp_liq,ca125))

foo<-bind_rows(
bind_cols(Biomarkers=rep("ca125",nrow(ca125)),ca125),
bind_cols(Biomarkers=rep("ibp_leg",nrow(ibp_leg)),ibp_leg),
bind_cols(Biomarkers=rep("ibp_liq",nrow(ibp_liq)),ibp_liq),
bind_cols(Biomarkers=rep("ibp_leg.ibp_liq",nrow(ibp_leg.ibp_liq)),ibp_leg.ibp_liq)
)

tiff("p7.tiff", units="in", width=5, height=5, res=600)

ggplot(foo,aes(d=df_temp.labelz,m=fitted.results,color=Biomarkers))+
 geom_roc(n.cuts=0
 ,show.legend = FALSE) + ####turn off or on legend
 style_roc(theme = theme_bw
 , xlab = "1 - Specificity"
 , ylab="Sensitivity") +
 scale_color_discrete(breaks=c('ca125','ibp_leg','ibp_liq',
 'ibp_leg.ibp_liq'),
 labels=c('CA125 (AUC: 0.00)'
 ,'IBP2 LEG (AUC: 0.00)'
 ,'IBP2 LIQ (AUC: 0.00 )'
 ,'IBP2 LEG + IBP2 LIQ (AUC: 0.00)'))+
 ggtitle("Benign vs late-stage")#+
 #theme(legend.justification = c(1, 0), legend.position = c(1, .5))
dev.off()

png
 2

foo<-bind_rows(
bind_cols(Biomarkers=rep("ca125",nrow(ca125)),ca125),
bind_cols(Biomarkers=rep("ibp_leg",nrow(ibp_leg)),ibp_leg),
bind_cols(Biomarkers=rep("ibp_liq",nrow(ibp_liq)),ibp_liq),
bind_cols(Biomarkers=rep("ibp_leg.ibp_liq",nrow(ibp_leg.ibp_liq)),ibp_leg.ibp_liq)
)

tiff("p8.tiff", units="in", width=5, height=5, res=600)

ggplot(foo,aes(d=df_temp.labelz,m=fitted.results,color=Biomarkers))+
 geom_roc(n.cuts=0
 ,show.legend = TRUE) +
 style_roc(theme = theme_bw
 , xlab = "1 - Specificity"
 , ylab="Sensitivity") +
 scale_color_discrete(breaks=c('ca125','ibp_leg','ibp_liq',
 'ibp_leg.ibp_liq'),
 labels=c('CA125 (AUC: 0.987)'
 ,'IBP2 LEG (AUC: 0.712)'
 ,'IBP2 LIQ (AUC: 0.774)'
 ,'IBP2 LEG + IBP2 LIQ (AUC: 0.754)'))+
 ggtitle("Benign vs late-stage")#+
 #theme(legend.justification = c(1, 0), legend.position = c(1, .5))
dev.off()

png
 2

foo<-bind_rows(
bind_cols(Biomarkers=rep("ca125",nrow(ca125)),ca125),
bind_cols(Biomarkers=rep("ibp_leg.ca125",nrow(ibp_leg.ca125)),ibp_leg.ca125),
bind_cols(Biomarkers=rep("ibp_liq.ca125",nrow(ibp_liq.ca125)),ibp_liq.ca125)
)

tiff("p9.tiff", units="in", width=5, height=5, res=600)

ggplot(foo,aes(d=df_temp.labelz,m=fitted.results,color=Biomarkers))+
 geom_roc(n.cuts=0
 ,show.legend = FALSE) + ####turn off or on legend
 style_roc(theme = theme_bw
 , xlab = "1 - Specificity"
 , ylab="Sensitivity") +
 scale_color_discrete(breaks=c('ca125','ibp_leg.ca125','ibp_liq.ca125'
 ),
 labels=c('CA125 (AUC: 0.00)'
 ,'IBP2 LEG + CA125 (AUC: 0.00)'
 ,'IBP2 LIQ + CA125 (AUC: 0.00 )'))+
 ggtitle("Benign vs late-stage")#+
 #theme(legend.justification = c(1, 0), legend.position = c(1, .5))
dev.off()

png
 2

foo<-bind_rows(
bind_cols(Biomarkers=rep("ca125",nrow(ca125)),ca125),
bind_cols(Biomarkers=rep("ibp_leg.ca125",nrow(ibp_leg.ca125)),ibp_leg.ca125),
bind_cols(Biomarkers=rep("ibp_liq.ca125",nrow(ibp_liq.ca125)),ibp_liq.ca125)
)

tiff("p10.tiff", units="in", width=5, height=5, res=600)

ggplot(foo,aes(d=df_temp.labelz,m=fitted.results,color=Biomarkers))+
 geom_roc(n.cuts=0
 ,show.legend = TRUE) + ####turn off or on legend
 style_roc(theme = theme_bw
 , xlab = "1 - Specificity"
 , ylab="Sensitivity") +
 scale_color_discrete(breaks=c('ca125','ibp_leg.ca125','ibp_liq.ca125'
 ),
 labels=c('CA125 (AUC: 0.987)'
 ,'IBP2 LEG + CA125 (AUC: 0.981)'
 ,'IBP2 LIQ + CA125 (AUC: 0.982)'))+
 ggtitle("Benign vs late-stage")#+
 #theme(legend.justification = c(1, 0), legend.position = c(1, .5))
dev.off()

png
 2

foo<-bind_rows(
bind_cols(Biomarkers=rep("ca125",nrow(ca125)),ca125),
bind_cols(Biomarkers=rep("ibp_leg.ibp_liq.ca125"
 ,nrow(ibp_leg.ibp_liq.ca125))
 ,ibp_leg.ibp_liq.ca125))

tiff("p11.tiff", units="in", width=5, height=5, res=600)

ggplot(foo,aes(d=df_temp.labelz,m=fitted.results,color=Biomarkers))+
 geom_roc(n.cuts=0
 ,show.legend = FALSE) + ####turn off or on legend
 style_roc(theme = theme_bw
 , xlab = "1 - Specificity"
 , ylab="Sensitivity") +
 scale_color_discrete(breaks=c('ca125','ibp.leg.ibp_liq.ca125'
 ),
 labels=c('CA125 (AUC: 0.00)'
 ,'IBP2 LEG +IBP2 LIQ + CA125 (AUC: 0.00 )'))+
 ggtitle("Benign vs late-stage")#+
 #theme(legend.justification = c(1, 0), legend.position = c(1, .5))
dev.off()

png
 2

foo<-bind_rows(
bind_cols(Biomarkers=rep("ca125",nrow(ca125)),ca125),
bind_cols(Biomarkers=rep("ibp_leg.ibp_liq.ca125"
 ,nrow(ibp_leg.ibp_liq.ca125))
 ,ibp_leg.ibp_liq.ca125))

tiff("p12.tiff", units="in", width=5, height=5, res=600)

ggplot(foo,aes(d=df_temp.labelz,m=fitted.results,color=Biomarkers))+
 geom_roc(n.cuts=0
 ,show.legend = TRUE) + ####turn off or on legend
 style_roc(theme = theme_bw
 , xlab = "1 - Specificity"
 , ylab="Sensitivity") +
 scale_color_discrete(breaks=c('ca125','ibp_leg.ibp_liq.ca125'
 ),
 labels=c('CA125 (AUC: 0.987)'
 ,'IBP2 LEG +IBP2 LIQ + CA125 (AUC: 0.985)'))+
 ggtitle("Benign vs late-stage")#+
 #theme(legend.justification = c(1, 0), legend.position = c(1, .5))
dev.off()

png
 2

**PART 1**

# 1. Summaries

Table 1.0 Table of summaries for the dataset. The dataset name is *df*. This table is overall summaries, not broken down by sample_group. It is primarily used to identify any problems in the dataset (e.g., Is the number of rows correct, and the number of columns? Is there the right number of character and numeric data columns? Do the distributions of the variable look correct?)

Data summary

Name

df

Number of rows

69

Number of columns

7

_______________________

Column type frequency:

character

1

numeric

6

________________________

Group variables

None

**Variable type: character**

skim_variable

n_missing

complete_rate

min

max

empty

n_unique

whitespace

sample_group

0

1

6

13

0

4

0

**Variable type: numeric**

skim_variable

n_missing

complete_rate

mean

sd

p0

p25

p50

p75

p100

hist

ibp_leg

11

0.84

16.65

11.36

4.08

8.92

13.51

19.69

57.20

▇▃▁▁▁

ibp_liq

4

0.94

6.28

4.63

0.71

3.29

5.34

8.00

22.49

▇▅▂▁▁

shbg_qae

6

0.91

91.74

44.45

15.84

61.49

88.59

114.83

260.02

▅▇▃▁▁

shbg_ldv

25

0.64

7.15

3.67

3.15

4.64

6.26

8.35

18.84

▇▆▁▁▁

timp1_gfq

15

0.78

8.41

3.15

5.24

6.70

7.44

9.06

22.70

▇▂▁▁▁

timp1_see

28

0.59

17.07

9.92

8.41

11.92

14.41

17.04

65.12

▇▂▁▁▁

Table 1.1 Summaries by group. Medians and interquartile ranges used for robustness. The p-values are for non-parametric ANOVA. P<0.05 indicates that at least one group (NC, L, B, E) is different from another group.

mycontrols <- tableby.control(test=TRUE, total=FALSE,
 numeric.test="kwt", cat.test="chisq",
 numeric.stats=c("N", "median", "q1q3"),
 # numeric.stats=c("N", "meansd"),
 cat.stats=c("countpct"),
 stats.labels=list(N='Count', median='Median', q1q3='Q1,Q3'))

tab2 <- tableby(sample_group ~ ., data=df, control=mycontrols) #-2 removes sample id
summary(tab2)

|  | Benign (N=18) | Early-stage (N=16) | Late-stage (N=17) | Non-cancerous (N=18) | p value |
| --- | --- | --- | --- | --- | --- |
| **ibp_leg** |  |  |  |  | 0.091 |
| Count | 13 | 16 | 16 | 13 |  |
| Median | 12.244 | 11.244 | 17.180 | 12.937 |  |
| Q1,Q3 | 8.618, 16.591 | 7.736, 16.648 | 14.022, 31.604 | 8.744, 18.096 |  |
| **ibp_liq** |  |  |  |  | 0.023 |
| Count | 18 | 14 | 15 | 18 |  |
| Median | 4.684 | 4.712 | 8.003 | 4.684 |  |
| Q1,Q3 | 2.804, 5.697 | 3.442, 6.831 | 5.350, 14.106 | 2.804, 5.697 |  |
| **shbg_qae** |  |  |  |  | 0.264 |
| Count | 15 | 16 | 16 | 16 |  |
| Median | 85.863 | 88.184 | 109.347 | 77.909 |  |
| Q1,Q3 | 53.681, 109.987 | 66.177, 92.057 | 84.343, 133.726 | 51.386, 106.138 |  |
| **shbg_ldv** |  |  |  |  | 0.067 |
| Count | 8 | 11 | 14 | 11 |  |
| Median | 4.701 | 5.712 | 7.995 | 7.698 |  |
| Q1,Q3 | 3.798, 5.140 | 3.839, 6.555 | 5.710, 8.391 | 5.750, 9.993 |  |
| **timp1_gfq** |  |  |  |  | 0.022 |
| Count | 12 | 15 | 13 | 14 |  |
| Median | 6.818 | 7.628 | 10.196 | 7.006 |  |
| Q1,Q3 | 6.272, 8.102 | 6.789, 9.211 | 7.057, 11.989 | 6.250, 7.453 |  |
| **timp1_see** |  |  |  |  | 0.472 |
| Count | 12 | 12 | 10 | 7 |  |
| Median | 12.986 | 15.232 | 14.821 | 12.886 |  |
| Q1,Q3 | 11.489, 16.848 | 14.245, 17.846 | 11.702, 22.224 | 12.369, 14.235 |  |

Table 1.2 Summaries by group, means and standard deviations. The p-values are for ANOVA. P<0.05 indicates that at least one group is different from another group.

mycontrols <- tableby.control(test=TRUE, total=FALSE,
 numeric.test="kwt", cat.test="chisq",
 #numeric.stats=c("N", "median", "q1q3"),
 numeric.stats=c("N", "meansd", "meanse"),
 cat.stats=c("countpct"),
 stats.labels=list(N='Count', median='Median', q1q3='Q1,Q3'))

tab2 <- tableby(sample_group ~ ., data=df, control=mycontrols)
summary(tab2)

|  | Benign (N=18) | Early-stage (N=16) | Late-stage (N=17) | Non-cancerous (N=18) | p value |
| --- | --- | --- | --- | --- | --- |
| **ibp_leg** |  |  |  |  | 0.091 |
| Count | 13 | 16 | 16 | 13 |  |
| Mean (SD) | 13.250 (7.026) | 14.685 (10.871) | 23.679 (14.941) | 13.817 (6.683) |  |
| Mean (SE) | 13.250 (1.949) | 14.685 (2.718) | 23.679 (3.735) | 13.817 (1.853) |  |
| **ibp_liq** |  |  |  |  | 0.023 |
| Count | 18 | 14 | 15 | 18 |  |
| Mean (SD) | 4.769 (2.905) | 6.310 (4.798) | 9.895 (6.012) | 4.769 (2.905) |  |
| Mean (SE) | 4.769 (0.685) | 6.310 (1.282) | 9.895 (1.552) | 4.769 (0.685) |  |
| **shbg_qae** |  |  |  |  | 0.264 |
| Count | 15 | 16 | 16 | 16 |  |
| Mean (SD) | 87.263 (47.668) | 85.576 (30.464) | 106.158 (30.476) | 87.699 (62.361) |  |
| Mean (SE) | 87.263 (12.308) | 85.576 (7.616) | 106.158 (7.619) | 87.699 (15.590) |  |
| **shbg_ldv** |  |  |  |  | 0.067 |
| Count | 8 | 11 | 14 | 11 |  |
| Mean (SD) | 5.447 (3.006) | 6.221 (3.215) | 7.340 (2.438) | 9.074 (5.103) |  |
| Mean (SE) | 5.447 (1.063) | 6.221 (0.969) | 7.340 (0.652) | 9.074 (1.539) |  |
| **timp1_gfq** |  |  |  |  | 0.022 |
| Count | 12 | 15 | 13 | 14 |  |
| Mean (SD) | 7.761 (2.881) | 8.279 (2.407) | 10.739 (4.430) | 6.943 (1.008) |  |
| Mean (SE) | 7.761 (0.832) | 8.279 (0.621) | 10.739 (1.229) | 6.943 (0.269) |  |
| **timp1_see** |  |  |  |  | 0.472 |
| Count | 12 | 12 | 10 | 7 |  |
| Mean (SD) | 19.508 (16.746) | 16.917 (5.210) | 17.071 (6.333) | 13.160 (1.524) |  |
| Mean (SE) | 19.508 (4.834) | 16.917 (1.504) | 17.071 (2.003) | 13.160 (0.576) |  |

# 2. Post-hoc tests

These are pairwise Wilcoxon rank-sum tests. False Discovery is corrected with the Holm method.

- The first column is the biomarker
- The second and third columns are the pairs of groups being tested.
- The adjusted p-value is in the second-to-last column.
- The last column gives a “heads up” about which pairs of groups are statistically significant.

For example, in Table 2.0, the biomarker is ibp2_leg. In the first row, group B is compared to group E. The sample sizes are 13 and 14 respectively. The p-value is 0.825, which is not statistically significant.

Table 2.0

| .y. | group1 | group2 | n1 | n2 | statistic | p | p.adj | p.adj.signif |
| --- | --- | --- | --- | --- | --- | --- | --- | --- |
| ibp_leg | Benign | Early-stage | 13 | 16 | 106 | 0.948 | 1.000 | ns |
| ibp_leg | Benign | Late-stage | 13 | 16 | 60 | 0.056 | 0.278 | ns |
| ibp_leg | Benign | Non-cancerous | 13 | 13 | 78 | 0.762 | 1.000 | ns |
| ibp_leg | Early-stage | Late-stage | 16 | 16 | 71 | 0.032 | 0.191 | ns |
| ibp_leg | Early-stage | Non-cancerous | 16 | 13 | 96 | 0.746 | 1.000 | ns |
| ibp_leg | Late-stage | Non-cancerous | 16 | 13 | 148 | 0.056 | 0.278 | ns |

Table 2.1

| .y. | group1 | group2 | n1 | n2 | statistic | p | p.adj | p.adj.signif |
| --- | --- | --- | --- | --- | --- | --- | --- | --- |
| ibp_liq | Benign | Early-stage | 18 | 14 | 106 | 0.464 | 1.000 | ns |
| ibp_liq | Benign | Late-stage | 18 | 15 | 61 | 0.007 | 0.040 | * |
| ibp_liq | Benign | Non-cancerous | 18 | 18 | 162 | 1.000 | 1.000 | ns |
| ibp_liq | Early-stage | Late-stage | 14 | 15 | 62 | 0.063 | 0.252 | ns |
| ibp_liq | Early-stage | Non-cancerous | 14 | 18 | 146 | 0.464 | 1.000 | ns |
| ibp_liq | Late-stage | Non-cancerous | 15 | 18 | 209 | 0.007 | 0.040 | * |

Table 2.2

| .y. | group1 | group2 | n1 | n2 | statistic | p | p.adj | p.adj.signif |
| --- | --- | --- | --- | --- | --- | --- | --- | --- |
| shbg_qae | Benign | Early-stage | 15 | 16 | 118 | 0.953 | 1.000 | ns |
| shbg_qae | Benign | Late-stage | 15 | 16 | 88 | 0.216 | 0.864 | ns |
| shbg_qae | Benign | Non-cancerous | 15 | 16 | 129 | 0.740 | 1.000 | ns |
| shbg_qae | Early-stage | Late-stage | 16 | 16 | 84 | 0.102 | 0.510 | ns |
| shbg_qae | Early-stage | Non-cancerous | 16 | 16 | 141 | 0.642 | 1.000 | ns |
| shbg_qae | Late-stage | Non-cancerous | 16 | 16 | 175 | 0.080 | 0.478 | ns |

Table 2.3

| .y. | group1 | group2 | n1 | n2 | statistic | p | p.adj | p.adj.signif |
| --- | --- | --- | --- | --- | --- | --- | --- | --- |
| shbg_ldv | Benign | Early-stage | 8 | 11 | 33 | 0.395 | 0.790 | ns |
| shbg_ldv | Benign | Late-stage | 8 | 14 | 29 | 0.070 | 0.349 | ns |
| shbg_ldv | Benign | Non-cancerous | 8 | 11 | 16 | 0.020 | 0.122 | ns |
| shbg_ldv | Early-stage | Late-stage | 11 | 14 | 50 | 0.149 | 0.447 | ns |
| shbg_ldv | Early-stage | Non-cancerous | 11 | 11 | 35 | 0.101 | 0.404 | ns |
| shbg_ldv | Late-stage | Non-cancerous | 14 | 11 | 72 | 0.809 | 0.809 | ns |

Table 2.4

| .y. | group1 | group2 | n1 | n2 | statistic | p | p.adj | p.adj.signif |
| --- | --- | --- | --- | --- | --- | --- | --- | --- |
| timp1_gfq | Benign | Early-stage | 12 | 15 | 71 | 0.373 | 0.746 | ns |
| timp1_gfq | Benign | Late-stage | 12 | 13 | 37 | 0.026 | 0.128 | ns |
| timp1_gfq | Benign | Non-cancerous | 12 | 14 | 88 | 0.860 | 0.860 | ns |
| timp1_gfq | Early-stage | Late-stage | 15 | 13 | 60 | 0.088 | 0.354 | ns |
| timp1_gfq | Early-stage | Non-cancerous | 15 | 14 | 144 | 0.093 | 0.354 | ns |
| timp1_gfq | Late-stage | Non-cancerous | 13 | 14 | 147 | 0.006 | 0.034 | * |

Table 2.5

| .y. | group1 | group2 | n1 | n2 | statistic | p | p.adj | p.adj.signif |
| --- | --- | --- | --- | --- | --- | --- | --- | --- |
| timp1_see | Benign | Early-stage | 12 | 12 | 52 | 0.266 | 1.000 | ns |
| timp1_see | Benign | Late-stage | 12 | 10 | 53 | 0.674 | 1.000 | ns |
| timp1_see | Benign | Non-cancerous | 12 | 7 | 42 | 1.000 | 1.000 | ns |
| timp1_see | Early-stage | Late-stage | 12 | 10 | 64 | 0.821 | 1.000 | ns |
| timp1_see | Early-stage | Non-cancerous | 12 | 7 | 63 | 0.083 | 0.499 | ns |
| timp1_see | Late-stage | Non-cancerous | 10 | 7 | 43 | 0.475 | 1.000 | ns |

# 3. Graphs

The boxplots give some idea of the data distributions. However, the larger values are distorting the scale, making the plots difficult to read.

plot(p2)


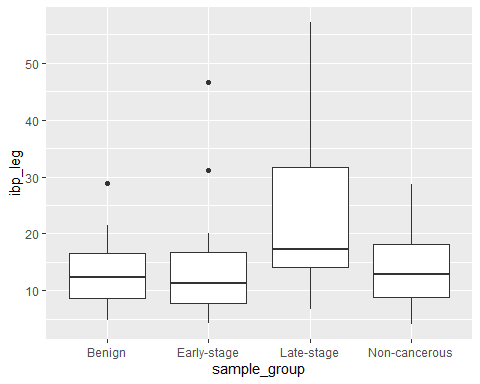


plot(p4)


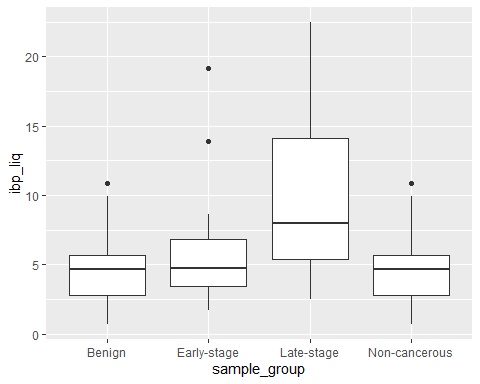


plot(p8)


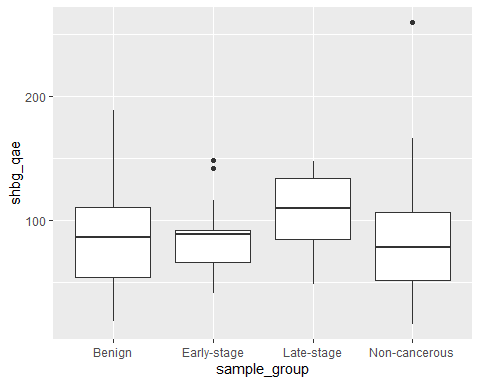


plot(p10)


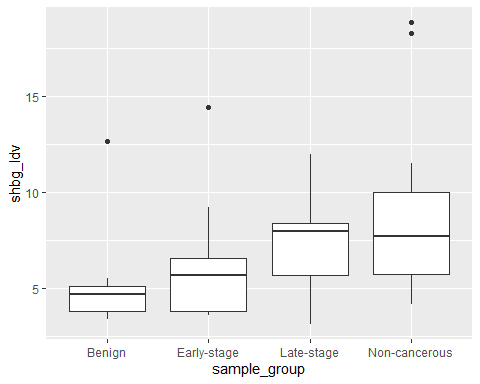


plot(p14)


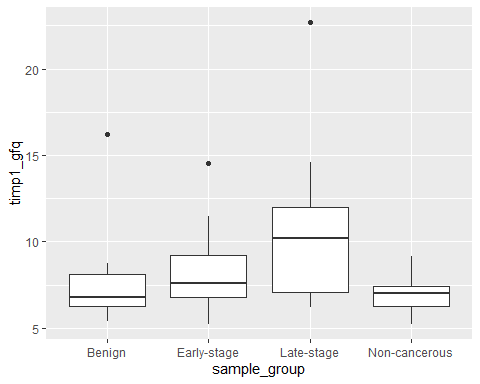


plot(p16)


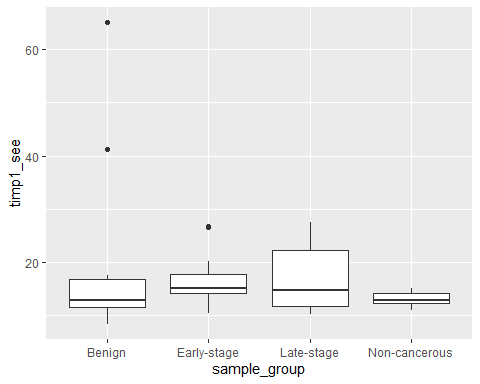


df2<-df %>%
 mutate(row_id=row_number()) %>%
 relocate(row_id) %>%
 pivot_longer(c("ibp_leg"
 ,"ibp_liq"
 ,"shbg_qae"
 ,"shbg_ldv"
 ,"timp1_gfq"
 ,"timp1_see")
 , names_to = c("combined.peptide", "extension")
 , names_sep = "_", values_to = "val") %>%
 pivot_wider(names_from="combined.peptide"
 ,values_from = "val") %>%
 unnest(cols = c("ibp","shbg","timp1")) %>%
 dplyr::select(-extension,-row_id)

**PART 2**

Data summary

Name

df2

Number of rows

414

Number of columns

4

_______________________

Column type frequency:

character

1

numeric

3

________________________

Group variables

None

**Variable type: character**

skim_variable

n_missing

complete_rate

min

max

empty

n_unique

whitespace

sample_group

0

1

6

13

0

4

0

**Variable type: numeric**

skim_variable

n_missing

complete_rate

mean

sd

p0

p25

p50

p75

p100

hist

ibp

291

0.30

11.17

9.93

0.71

4.71

8.12

14.48

57.20

▇▃▁▁▁

shbg

307

0.26

56.96

53.94

3.15

7.28

47.72

91.70

260.02

▇▅▂▁▁

timp1

319

0.23

12.15

8.13

5.24

7.16

10.28

14.47

65.12

▇▁▁▁▁

Table 1.1 Summaries by group. Medians and interquartile ranges used for robustness. The p-values are for non-parametric ANOVA. P<0.05 indicates that at least one group (NC, L, B, E) is different from another group.

mycontrols <- tableby.control(test=TRUE, total=FALSE,
 numeric.test="kwt", cat.test="chisq",
 numeric.stats=c("N", "median", "q1q3"),
 # numeric.stats=c("N", "meansd"),
 cat.stats=c("countpct"),
 stats.labels=list(N='Count', median='Median', q1q3='Q1,Q3'))

tab2 <- tableby(sample_group ~ ., data=df2, control=mycontrols)
summary(tab2)

|  | Benign (N=108) | Early-stage (N=96) | Late-stage (N=102) | Non-cancerous (N=108) | p value |
| --- | --- | --- | --- | --- | --- |
| **ibp** |  |  |  |  | 0.003 |
| Count | 31 | 30 | 31 | 31 |  |
| Median | 5.813 | 7.730 | 14.625 | 6.396 |  |
| Q1,Q3 | 4.144, 11.332 | 4.711, 13.682 | 7.366, 19.269 | 3.880, 12.218 |  |
| **shbg** |  |  |  |  | 0.938 |
| Count | 23 | 27 | 30 | 27 |  |
| Median | 51.400 | 52.233 | 53.684 | 25.925 |  |
| Q1,Q3 | 5.264, 93.113 | 6.260, 90.603 | 8.078, 111.059 | 8.275, 81.595 |  |
| **timp1** |  |  |  |  | 0.072 |
| Count | 24 | 27 | 23 | 21 |  |
| Median | 9.265 | 10.506 | 11.545 | 7.467 |  |
| Q1,Q3 | 6.850, 13.679 | 7.583, 14.843 | 10.180, 15.635 | 6.777, 11.921 |  |

Table 1.2 Summaries by group, means and standard deviations. The p-values are for ANOVA. P<0.05 indicates that at least one group is different from another group.

mycontrols <- tableby.control(test=TRUE, total=FALSE,
 numeric.test="kwt", cat.test="chisq",
 # numeric.stats=c("N", "median", "q1q3"),
 numeric.stats=c("N", "meansd"),
 cat.stats=c("countpct"),
 stats.labels=list(N='Count', median='Median', q1q3='Q1,Q3'))

tab2 <- tableby(sample_group ~ ., data=df2, control=mycontrols)
summary(tab2)

|  | Benign (N=108) | Early-stage (N=96) | Late-stage (N=102) | Non-cancerous (N=108) | p value |
| --- | --- | --- | --- | --- | --- |
| **ibp** |  |  |  |  | 0.003 |
| Count | 31 | 30 | 31 | 31 |  |
| Mean (SD) | 8.325 (6.529) | 10.777 (9.461) | 17.009 (13.323) | 8.563 (6.576) |  |
| **shbg** |  |  |  |  | 0.938 |
| Count | 23 | 27 | 30 | 27 |  |
| Mean (SD) | 58.806 (55.103) | 53.246 (46.024) | 60.043 (54.748) | 55.667 (61.672) |  |
| **timp1** |  |  |  |  | 0.072 |
| Count | 24 | 27 | 23 | 21 |  |
| Mean (SD) | 13.635 (13.194) | 12.118 (5.808) | 13.492 (6.117) | 9.015 (3.221) |  |

# 3. Post-hoc tests

These are pairwise Wilcoxon rank-sum tests. False Discovery is corrected with the Holm method.

- The first column is the biomarker
- The second and third columns are the pairs of groups being tested.
- The adjusted p-value is in the second-to-last column.
- The last column gives a “heads up” about which pairs of groups are statistically significant.

For example, in Table 2.0, the biomarker is ibp2_leg. In the first row, group B is compared to group E. The sample sizes are 13 and 14 respectively. The p-value is 0.825, which is not statistically significant.

Table 3.0

| .y. | group1 | group2 | n1 | n2 | statistic | p | p.adj | p.adj.signif |
| --- | --- | --- | --- | --- | --- | --- | --- | --- |
| ibp | Benign | Early-stage | 31 | 30 | 391 | 0.291 | 0.873 | ns |
| ibp | Benign | Late-stage | 31 | 31 | 254 | 0.001 | 0.007 | ** |
| ibp | Benign | Non-cancerous | 31 | 31 | 467 | 0.855 | 0.873 | ns |
| ibp | Early-stage | Late-stage | 30 | 31 | 306 | 0.021 | 0.086 | ns |
| ibp | Early-stage | Non-cancerous | 30 | 31 | 529 | 0.362 | 0.873 | ns |
| ibp | Late-stage | Non-cancerous | 31 | 31 | 703 | 0.001 | 0.007 | ** |

Table 3.1

| .y. | group1 | group2 | n1 | n2 | statistic | p | p.adj | p.adj.signif |
| --- | --- | --- | --- | --- | --- | --- | --- | --- |
| shbg | Benign | Early-stage | 23 | 27 | 316 | 0.923 | 1 | ns |
| shbg | Benign | Late-stage | 23 | 30 | 327 | 0.756 | 1 | ns |
| shbg | Benign | Non-cancerous | 23 | 27 | 308 | 0.969 | 1 | ns |
| shbg | Early-stage | Late-stage | 27 | 30 | 358 | 0.460 | 1 | ns |
| shbg | Early-stage | Non-cancerous | 27 | 27 | 352 | 0.837 | 1 | ns |
| shbg | Late-stage | Non-cancerous | 30 | 27 | 423 | 0.781 | 1 | ns |

Table 3.2

| .y. | group1 | group2 | n1 | n2 | statistic | p | p.adj | p.adj.signif |
| --- | --- | --- | --- | --- | --- | --- | --- | --- |
| timp1 | Benign | Early-stage | 24 | 27 | 295 | 0.594 | 0.812 | ns |
| timp1 | Benign | Late-stage | 24 | 23 | 215 | 0.200 | 0.800 | ns |
| timp1 | Benign | Non-cancerous | 24 | 21 | 304 | 0.244 | 0.800 | ns |
| timp1 | Early-stage | Late-stage | 27 | 23 | 267 | 0.406 | 0.812 | ns |
| timp1 | Early-stage | Non-cancerous | 27 | 21 | 381 | 0.043 | 0.216 | ns |
| timp1 | Late-stage | Non-cancerous | 23 | 21 | 347 | 0.013 | 0.076 | ns |

# 3. Graphs

The boxplots give some idea of the data distributions. However, the larger values are distorting the scale, making the plots difficult to read.

plot(p2)


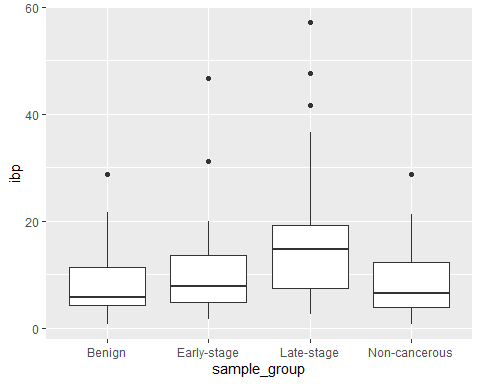


plot(p8)


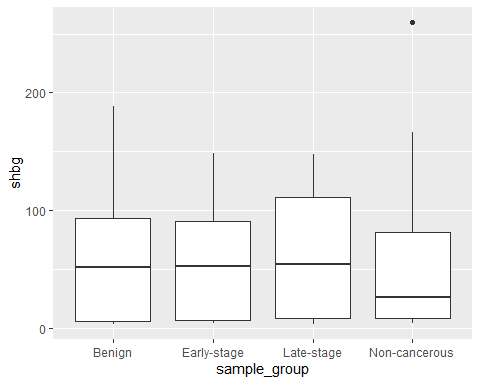


plot(p14)


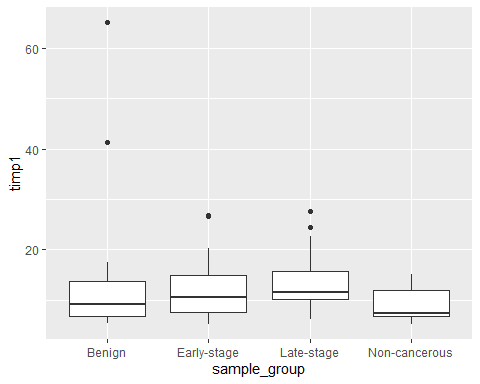


**PART 3**

**IBP2_LEG + TIMP1_GFQ**

Data summary

Name

df3

Number of rows

138

Number of columns

2

_______________________

Column type frequency:

character

1

numeric

1

________________________

Group variables

None

**Variable type: character**

skim_variable

n_missing

complete_rate

min

max

empty

n_unique

whitespace

sample_group

0

1

6

13

0

4

0

**Variable type: numeric**

skim_variable

n_missing

complete_rate

mean

sd

p0

p25

p50

p75

p100

hist

IBP2_LEG.TIMP1_GFQ

26

0.81

12.68

9.39

4.08

6.94

9.29

14.65

57.2

▇▂▁▁▁

Table 1.1 IBP2_LEG + TIMP1_GFQ Summaries by group. Medians and interquartile ranges used for robustness. The p-values are for non-parametric ANOVA. P<0.05 indicates that at least one group (NC, L, B, E) is different from another group.

mycontrols <- tableby.control(test=TRUE, total=FALSE,
 numeric.test="kwt", cat.test="chisq",
 numeric.stats=c("N", "median", "q1q3"),
 # numeric.stats=c("N", "meansd"),
 cat.stats=c("countpct"),
 stats.labels=list(N='Count', median='Median', q1q3='Q1,Q3'))

tab2 <- tableby(sample_group ~ ., data=df3, control=mycontrols)
summary(tab2)

|  | Benign (N=36) | Early-stage (N=32) | Late-stage (N=34) | Non-cancerous (N=36) | p value |
| --- | --- | --- | --- | --- | --- |
| **IBP2_LEG.TIMP1_GFQ** |  |  |  |  | 0.009 |
| Count | 25 | 31 | 29 | 27 |  |
| Median | 8.566 | 8.616 | 12.597 | 7.504 |  |
| Q1,Q3 | 6.365, 12.453 | 7.447, 12.328 | 10.164, 20.235 | 6.774, 12.693 |  |

Table 1.2 IBP2_LEG + TIMP1_GFQ Summaries by group, means and standard deviations. The p-values are for ANOVA. P<0.05 indicates that at least one group is different from another group.

mycontrols <- tableby.control(test=TRUE, total=FALSE,
 numeric.test="kwt", cat.test="chisq",
 # numeric.stats=c("N", "median", "q1q3"),
 numeric.stats=c("N", "meansd"),
 cat.stats=c("countpct"),
 stats.labels=list(N='Count', median='Median', q1q3='Q1,Q3'))

tab2 <- tableby(sample_group ~ ., data=df3, control=mycontrols)
summary(tab2)

|  | Benign (N=36) | Early-stage (N=32) | Late-stage (N=34) | Non-cancerous (N=36) | p value |
| --- | --- | --- | --- | --- | --- |
| **IBP2_LEG.TIMP1_GFQ** |  |  |  |  | 0.009 |
| Count | 25 | 31 | 29 | 27 |  |
| Mean (SD) | 10.615 (6.027) | 11.586 (8.508) | 17.878 (13.072) | 10.252 (5.777) |  |

# 3. Post-hoc tests IBP2_LEG + TIMP1_GFQ

These are pairwise Wilcoxon rank-sum tests. False Discovery is corrected with the Holm method.

- The first column is the biomarker
- The second and third columns are the pairs of groups being tested.
- The adjusted p-value is in the second-to-last column.
- The last column gives a “heads up” about which pairs of groups are statistically significant.

Table 3.0 IBP2_LEG + TIMP1_GFQ

| .y. | group1 | group2 | n1 | n2 | statistic | p | p.adj | p.adj.signif |
| --- | --- | --- | --- | --- | --- | --- | --- | --- |
| IBP2_LEG.TIMP1_GFQ | Benign | Early-stage | 25 | 31 | 361 | 0.671 | 1.000 | ns |
| IBP2_LEG.TIMP1_GFQ | Benign | Late-stage | 25 | 29 | 212 | 0.008 | 0.042 | * |
| IBP2_LEG.TIMP1_GFQ | Benign | Non-cancerous | 25 | 27 | 339 | 0.986 | 1.000 | ns |
| IBP2_LEG.TIMP1_GFQ | Early-stage | Late-stage | 31 | 29 | 275 | 0.009 | 0.042 | * |
| IBP2_LEG.TIMP1_GFQ | Early-stage | Non-cancerous | 31 | 27 | 476 | 0.377 | 1.000 | ns |
| IBP2_LEG.TIMP1_GFQ | Late-stage | Non-cancerous | 29 | 27 | 567 | 0.004 | 0.021 | * |

# 3. Graphs IBP2_LEG + TIMP1_GFQ

plot(p2)


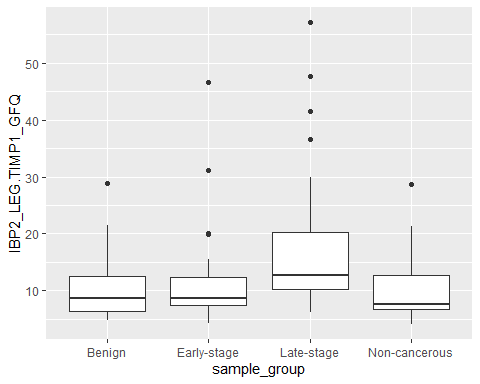


**IBP2_LIQ + TIMP1_GFQ**

Data summary

Name

df3

Number of rows

138

Number of columns

2

_______________________

Column type frequency:

character

1

numeric

1

________________________

Group variables

None

**Variable type: character**

skim_variable

n_missing

complete_rate

min

max

empty

n_unique

whitespace

sample_group

0

1

6

13

0

4

0

**Variable type: numeric**

skim_variable

n_missing

complete_rate

mean

sd

p0

p25

p50

p75

p100

hist

IBP2_LIQ.TIMP1_GFQ

19

0.86

7.25

4.15

0.71

4.73

6.73

8.63

22.7

▅▇▂▁▁

Table 1.1 IBP2_LIQ + TIMP1_GFQ Summaries by group. Medians and interquartile ranges used for robustness. The p-values are for non-parametric ANOVA. P<0.05 indicates that at least one group (NC, L, B, E) is different from another group.

mycontrols <- tableby.control(test=TRUE, total=FALSE,
 numeric.test="kwt", cat.test="chisq",
 numeric.stats=c("N", "median", "q1q3"),
 # numeric.stats=c("N", "meansd"),
 cat.stats=c("countpct"),
 stats.labels=list(N='Count', median='Median', q1q3='Q1,Q3'))

tab2 <- tableby(sample_group ~ ., data=df3, control=mycontrols)
summary(tab2)

|  | Benign (N=36) | Early-stage (N=32) | Late-stage (N=34) | Non-cancerous (N=36) | p value |
| --- | --- | --- | --- | --- | --- |
| **IBP2_LIQ.TIMP1_GFQ** |  |  |  |  | < 0.001 |
| Count | 30 | 29 | 28 | 32 |  |
| Median | 5.677 | 6.879 | 9.580 | 5.837 |  |
| Q1,Q3 | 3.910, 7.812 | 4.713, 8.639 | 6.681, 12.844 | 4.378, 7.425 |  |

Table 1.2 IBP2_LIQ + TIMP1_GFQ Summaries by group, means and standard deviations. The p-values are for ANOVA. P<0.05 indicates that at least one group is different from another group.

mycontrols <- tableby.control(test=TRUE, total=FALSE,
 numeric.test="kwt", cat.test="chisq",
 # numeric.stats=c("N", "median", "q1q3"),
 numeric.stats=c("N", "meansd"),
 cat.stats=c("countpct"),
 stats.labels=list(N='Count', median='Median', q1q3='Q1,Q3'))

tab2 <- tableby(sample_group ~ ., data=df3, control=mycontrols)
summary(tab2)

|  | Benign (N=36) | Early-stage (N=32) | Late-stage (N=34) | Non-cancerous (N=36) | p value |
| --- | --- | --- | --- | --- | --- |
| **IBP2_LIQ.TIMP1_GFQ** |  |  |  |  | < 0.001 |
| Count | 30 | 29 | 28 | 32 |  |
| Mean (SD) | 5.966 (3.212) | 7.329 (3.819) | 10.287 (5.258) | 5.720 (2.501) |  |

# 3. Post-hoc tests IBP2_LIQ + TIMP1_GFQ

These are pairwise Wilcoxon rank-sum tests. False Discovery is corrected with the Holm method.

- The first column is the biomarker
- The second and third columns are the pairs of groups being tested.
- The adjusted p-value is in the second-to-last column.
- The last column gives a “heads up” about which pairs of groups are statistically significant.

Table 3.0 IBP2_LIQ + TIMP1_GFQ

| .y. | group1 | group2 | n1 | n2 | statistic | p | p.adj | p.adj.signif |
| --- | --- | --- | --- | --- | --- | --- | --- | --- |
| IBP2_LIQ.TIMP1_GFQ | Benign | Early-stage | 30 | 29 | 348 | 0.1910 | 0.432 | ns |
| IBP2_LIQ.TIMP1_GFQ | Benign | Late-stage | 30 | 28 | 199 | 0.0004 | 0.002 | ** |
| IBP2_LIQ.TIMP1_GFQ | Benign | Non-cancerous | 30 | 32 | 484 | 0.9610 | 0.961 | ns |
| IBP2_LIQ.TIMP1_GFQ | Early-stage | Late-stage | 29 | 28 | 264 | 0.0230 | 0.092 | ns |
| IBP2_LIQ.TIMP1_GFQ | Early-stage | Non-cancerous | 29 | 32 | 566 | 0.1440 | 0.432 | ns |
| IBP2_LIQ.TIMP1_GFQ | Late-stage | Non-cancerous | 28 | 32 | 690 | 0.0002 | 0.001 | ** |

# 3. Graphs IBP2_LIQ + TIMP1_GFQ

plot(p2)


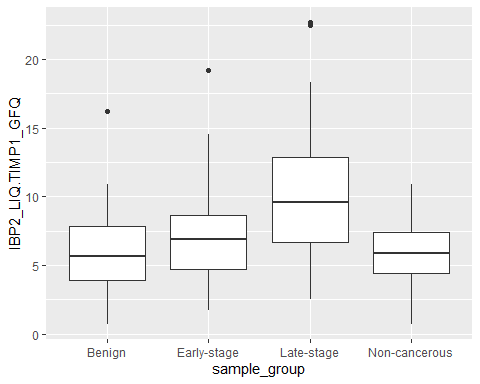


**IBP2_LEG + IBP2_LIQ + TIMP1_GFQ**

Data summary

Name

df3

Number of rows

207

Number of columns

2

_______________________

Column type frequency:

character

1

numeric

1

________________________

Group variables

None

**Variable type: character**

skim_variable

n_missing

complete_rate

min

max

empty

n_unique

whitespace

sample_group

0

1

6

13

0

4

0

**Variable type: numeric**

skim_variable

n_missing

complete_rate

mean

sd

p0

p25

p50

p75

p100

hist

IBP2_LEG.IBP2_LIQ.TIMP1_GFQ

30

0.86

10.33

8.54

0.71

5.38

7.72

12.45

57.2

▇▂▁▁▁

Table 1.1 IBP2_LEG + IBP2_LIQ + TIMP1_GFQ Summaries by group. Medians and interquartile ranges used for robustness. The p-values are for non-parametric ANOVA. P<0.05 indicates that at least one group (NC, L, B, E) is different from another group.

mycontrols <- tableby.control(test=TRUE, total=FALSE,
 numeric.test="kwt", cat.test="chisq",
 numeric.stats=c("N", "median", "q1q3"),
 # numeric.stats=c("N", "meansd"),
 cat.stats=c("countpct"),
 stats.labels=list(N='Count', median='Median', q1q3='Q1,Q3'))

tab2 <- tableby(sample_group ~ ., data=df3, control=mycontrols)
summary(tab2)

|  | Benign (N=54) | Early-stage (N=48) | Late-stage (N=51) | Non-cancerous (N=54) | p value |
| --- | --- | --- | --- | --- | --- |
| **IBP2_LEG.IBP2_LIQ.TIMP1_GFQ** |  |  |  |  | < 0.001 |
| Count | 43 | 45 | 44 | 45 |  |
| Median | 6.703 | 7.719 | 11.541 | 7.002 |  |
| Q1,Q3 | 4.762, 9.674 | 5.564, 11.482 | 7.025, 17.313 | 5.237, 9.148 |  |

Table 1.2 IBP2_LEG + IBP2_LIQ + TIMP1_GFQ Summaries by group, means and standard deviations. The p-values are for ANOVA. P<0.05 indicates that at least one group is different from another group.

mycontrols <- tableby.control(test=TRUE, total=FALSE,
 numeric.test="kwt", cat.test="chisq",
 # numeric.stats=c("N", "median", "q1q3"),
 numeric.stats=c("N", "meansd"),
 cat.stats=c("countpct"),
 stats.labels=list(N='Count', median='Median', q1q3='Q1,Q3'))

tab2 <- tableby(sample_group ~ ., data=df3, control=mycontrols)
summary(tab2)

|  | Benign (N=54) | Early-stage (N=48) | Late-stage (N=51) | Non-cancerous (N=54) | p value |
| --- | --- | --- | --- | --- | --- |
| **IBP2_LEG.IBP2_LIQ.TIMP1_GFQ** |  |  |  |  | < 0.001 |
| Count | 43 | 45 | 44 | 45 |  |
| Mean (SD) | 8.168 (5.717) | 9.944 (7.890) | 15.157 (11.734) | 8.059 (5.510) |  |

# 3. Post-hoc tests IBP2_LEG + IBP2_LIQ + TIMP1_GFQ

These are pairwise Wilcoxon rank-sum tests. False Discovery is corrected with the Holm method.

Table 3.0

| .y. | group1 | group2 | n1 | n2 | statistic | p | p.adj | p.adj.signif |
| --- | --- | --- | --- | --- | --- | --- | --- | --- |
| IBP2_LEG.IBP2_LIQ.TIMP1_GFQ | Benign | Early-stage | 43 | 45 | 817 | 0.2120 | 0.4980 | ns |
| IBP2_LEG.IBP2_LIQ.TIMP1_GFQ | Benign | Late-stage | 43 | 44 | 507 | 0.0001 | 0.0007 | *** |
| IBP2_LEG.IBP2_LIQ.TIMP1_GFQ | Benign | Non-cancerous | 43 | 45 | 962 | 0.9670 | 0.9670 | ns |
| IBP2_LEG.IBP2_LIQ.TIMP1_GFQ | Early-stage | Late-stage | 45 | 44 | 652 | 0.0050 | 0.0210 | * |
| IBP2_LEG.IBP2_LIQ.TIMP1_GFQ | Early-stage | Non-cancerous | 45 | 45 | 1185 | 0.1660 | 0.4980 | ns |
| IBP2_LEG.IBP2_LIQ.TIMP1_GFQ | Late-stage | Non-cancerous | 44 | 45 | 1456 | 0.0001 | 0.0006 | *** |

# 3. Graphs IBP2_LEG + IBP2_LIQ + TIMP1_GFQ

plot(p2)


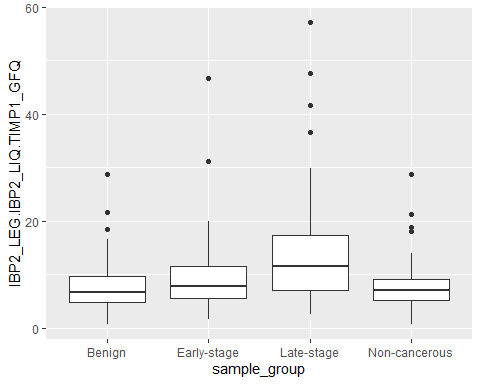


**End of Document**
