# Supplementary figures and images for: Quantification of putative ovarian cancer serum protein biomarkers using a multiplexed targeted mass spectrometry assay
Source: Clin Proteomics. 2024 Jan 3;21:1. doi: 10.1186/s12014-023-09447-4 (PMC10762856; doi:10.1186/s12014-023-09447-4)

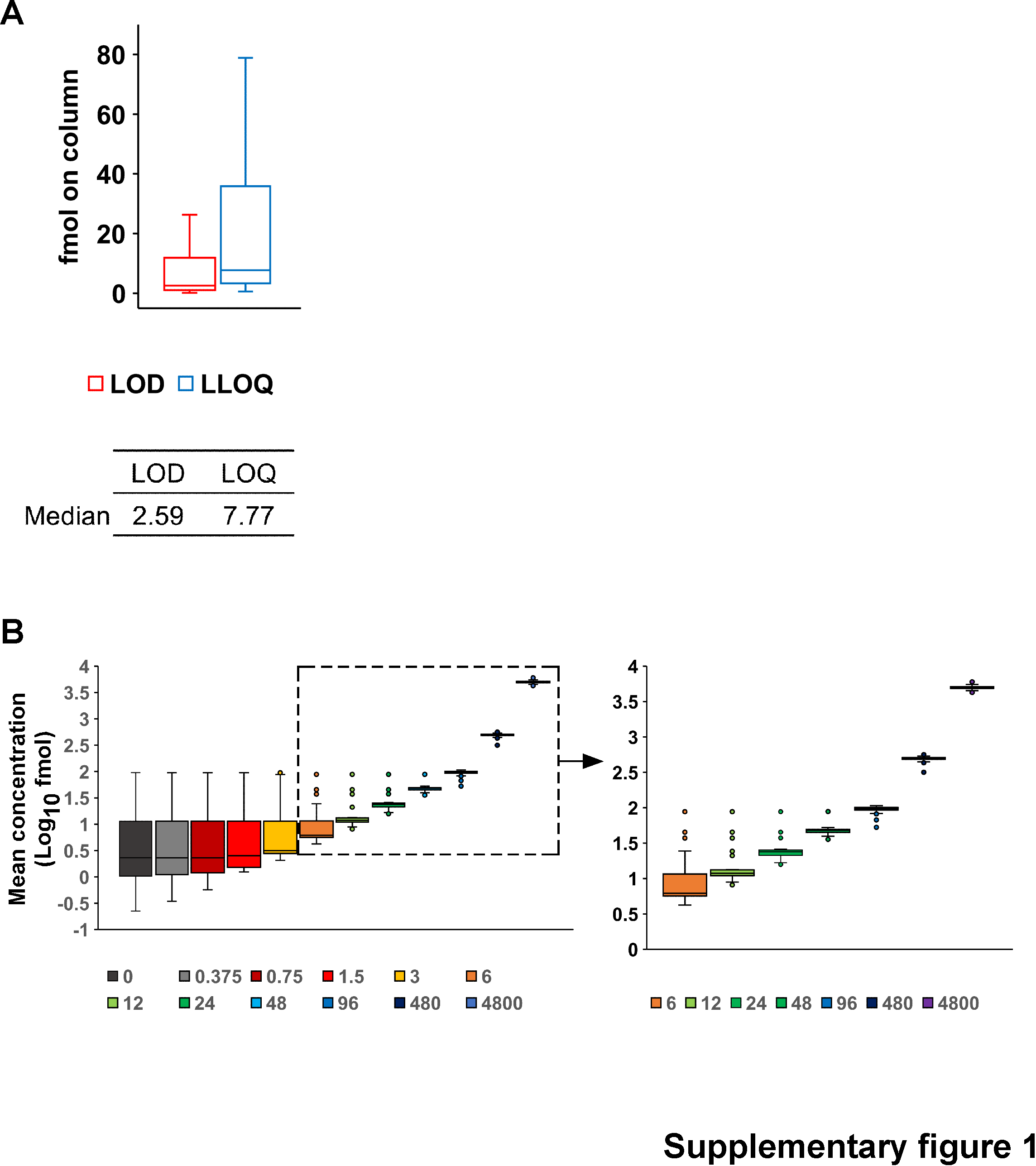

Supplement: Supplementary file 2 — Additional file 2: Figure S1. Calibration curve-based assay performance for 64 peptides. A LOD and LOQ. The horizontal red and blue lines in each box represent the median peptide LOD and LOQ in each matrix. The y-axis indicates the on-column concentration based on a 10 µL injection volume. The median LOD and LOQ is included in the table below the plot. B Evaluation of the accuracy of the observed abundance of each peptide standard. Enlarged range of the calibration curve from 6 - 4800 fmol. [file 12014_2023_9447_MOESM2_ESM.tif]

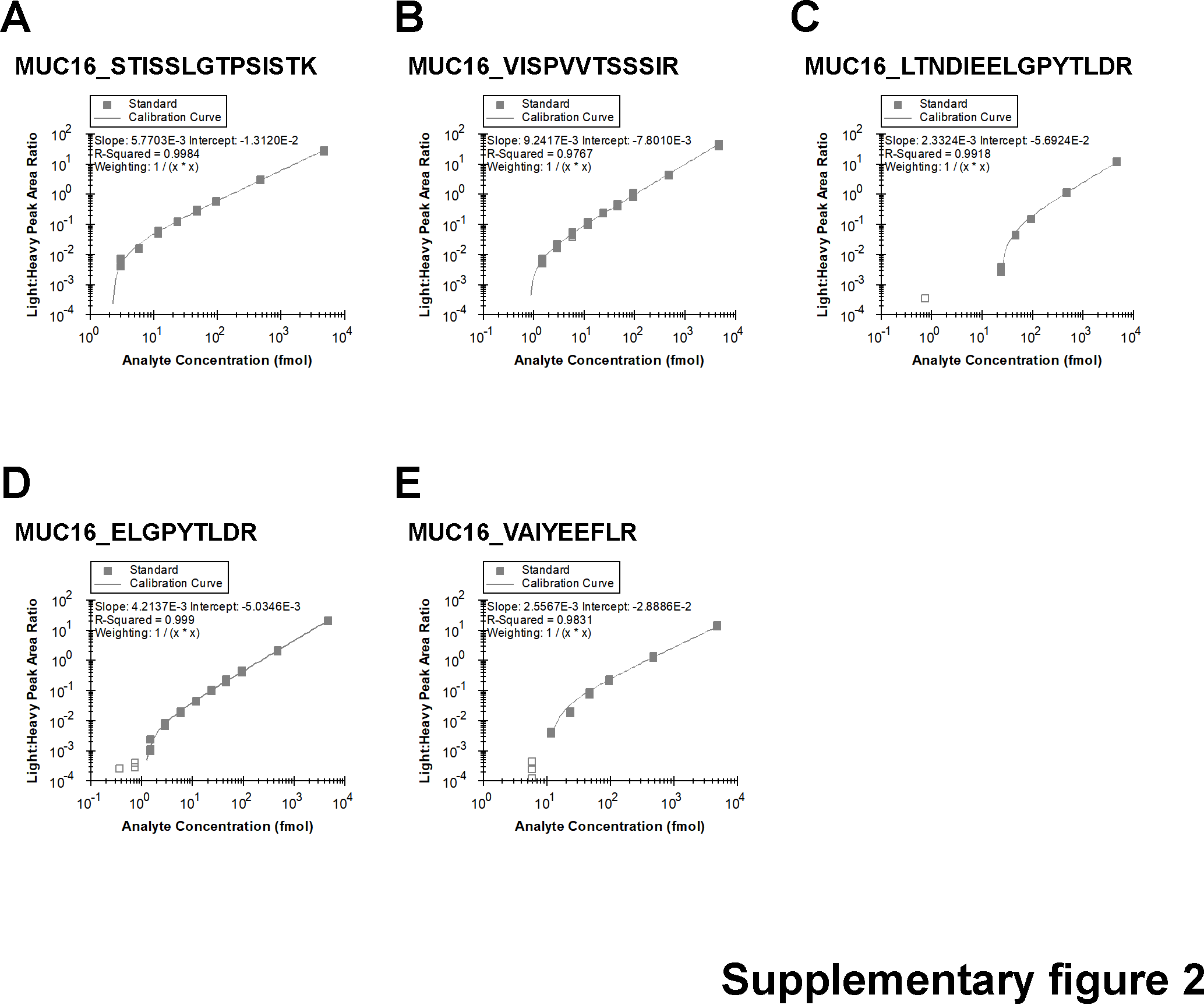

Supplement: Supplementary file 3 — Additional file 3: Figure S2. Calibration curves of peptides corresponding to MUC16 protein. Gray square indicates each standard point. White square indicates standard points that were excluded from calibration curve with the accuracy of > 20%. Black line indicates the calibration curves. [file 12014_2023_9447_MOESM3_ESM.tif]

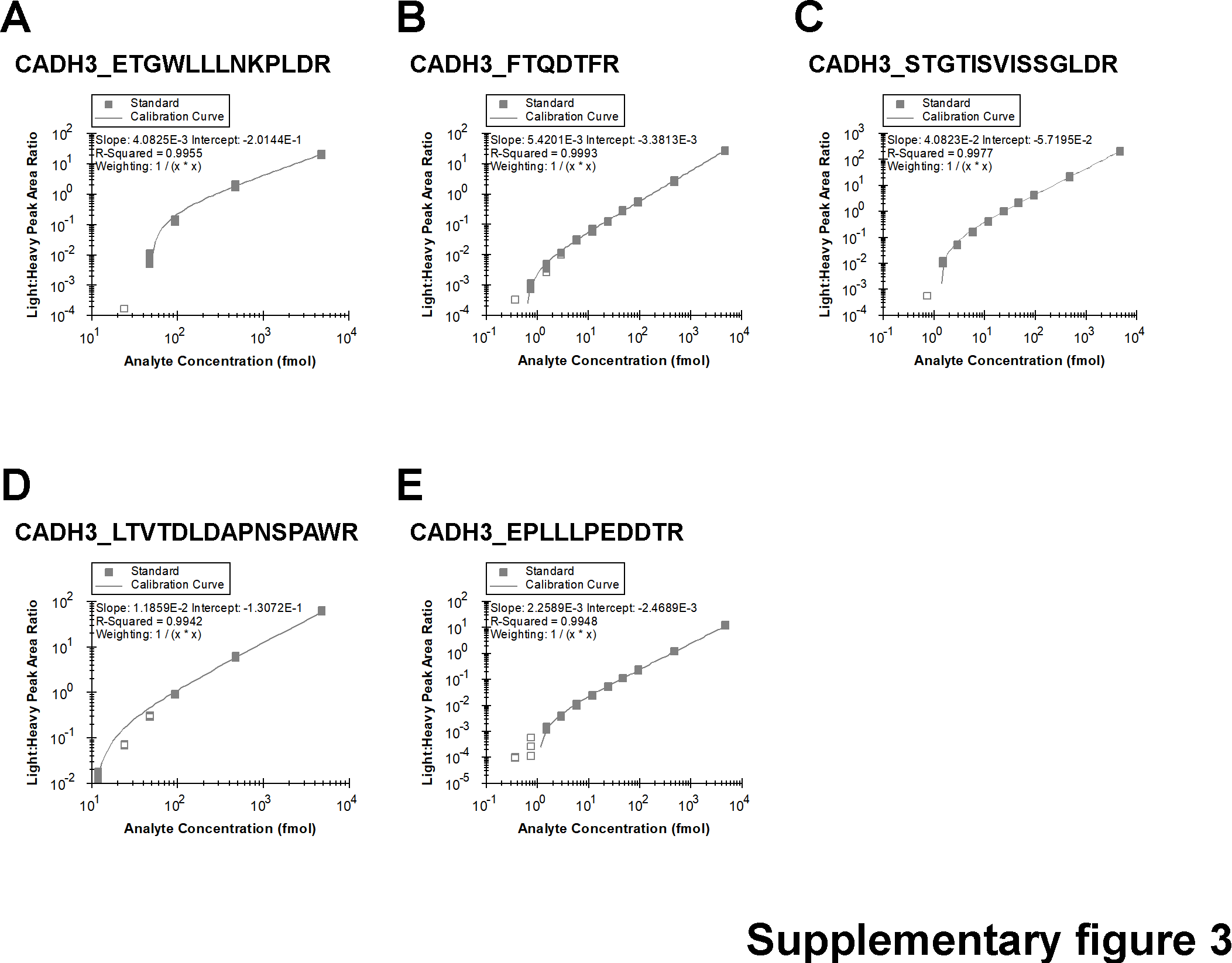

Supplement: Supplementary file 4 — Additional file 4: Figure S3. Calibration curves of peptides corresponding to CADH3 protein. Gray square indicates each standard point. White square indicates standard points that were excluded from calibration curve with the accuracy of > 20%. Black line indicates the calibration curves. [file 12014_2023_9447_MOESM4_ESM.tif]

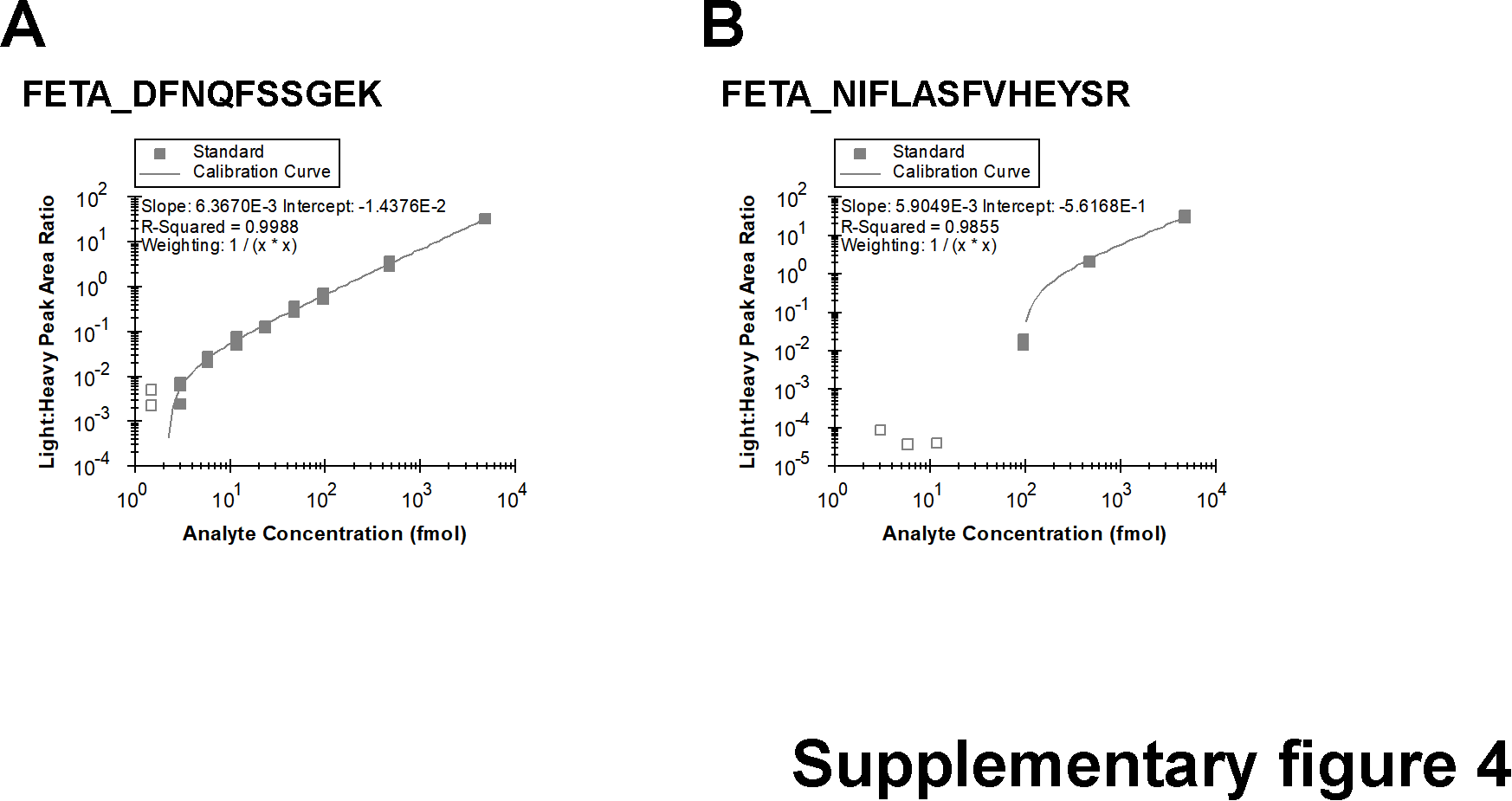

Supplement: Supplementary file 5 — Additional file 5: Figure S4. Calibration curves of peptides corresponding to FETA protein. Gray square indicates each standard point. White square indicates standard points that were excluded from calibration curve with the accuracy of > 20%. Black line indicates the calibration curves. [file 12014_2023_9447_MOESM5_ESM.tif]

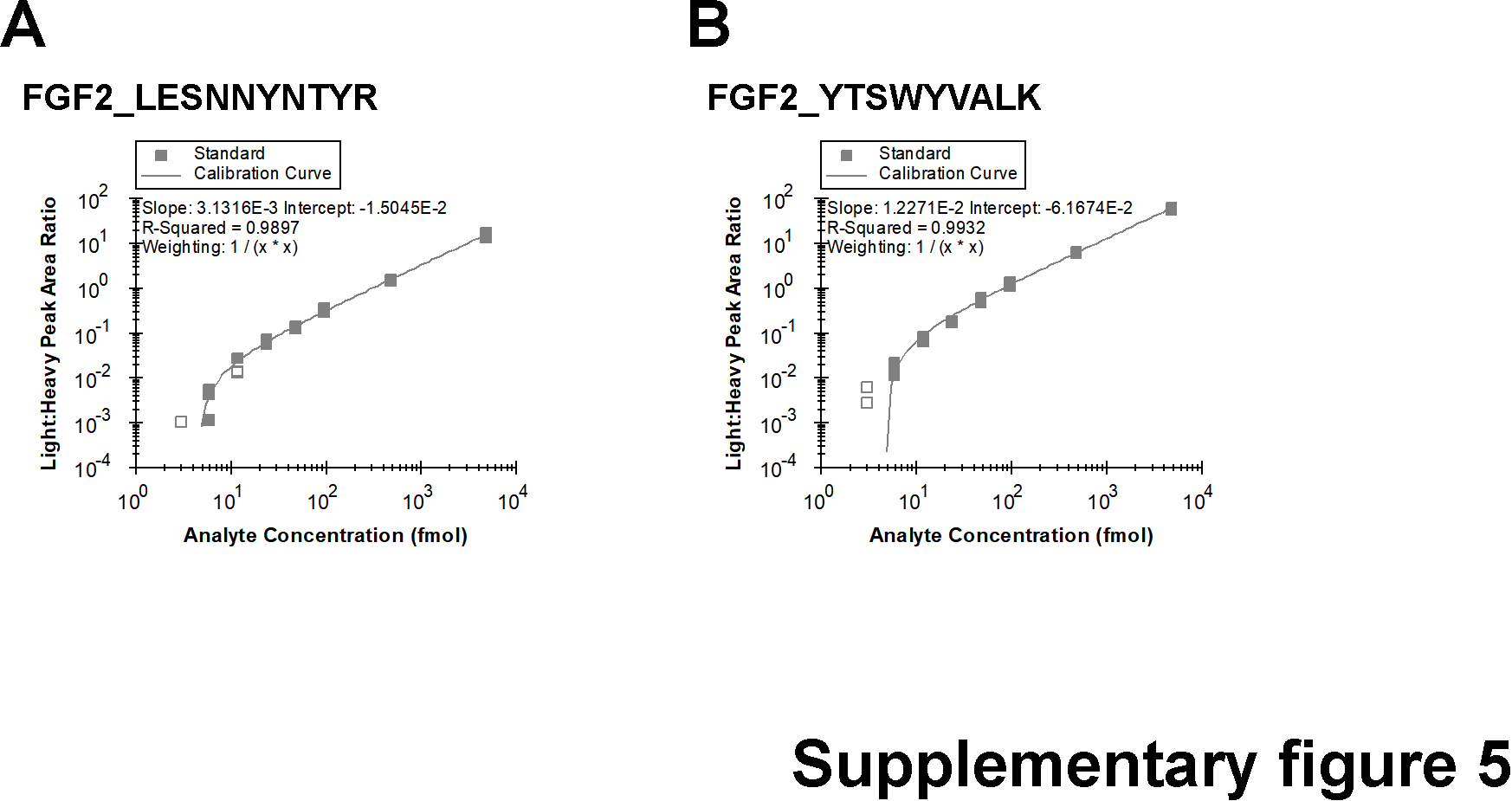

Supplement: Supplementary file 6 — Additional file 6: Figure S5. Calibration curves of peptides corresponding to FGF2 protein. Gray square indicates each standard point. White square indicates standard points that were excluded from calibration curve with the accuracy of > 20%. Black line indicates the calibration curves. [file 12014_2023_9447_MOESM6_ESM.tif]

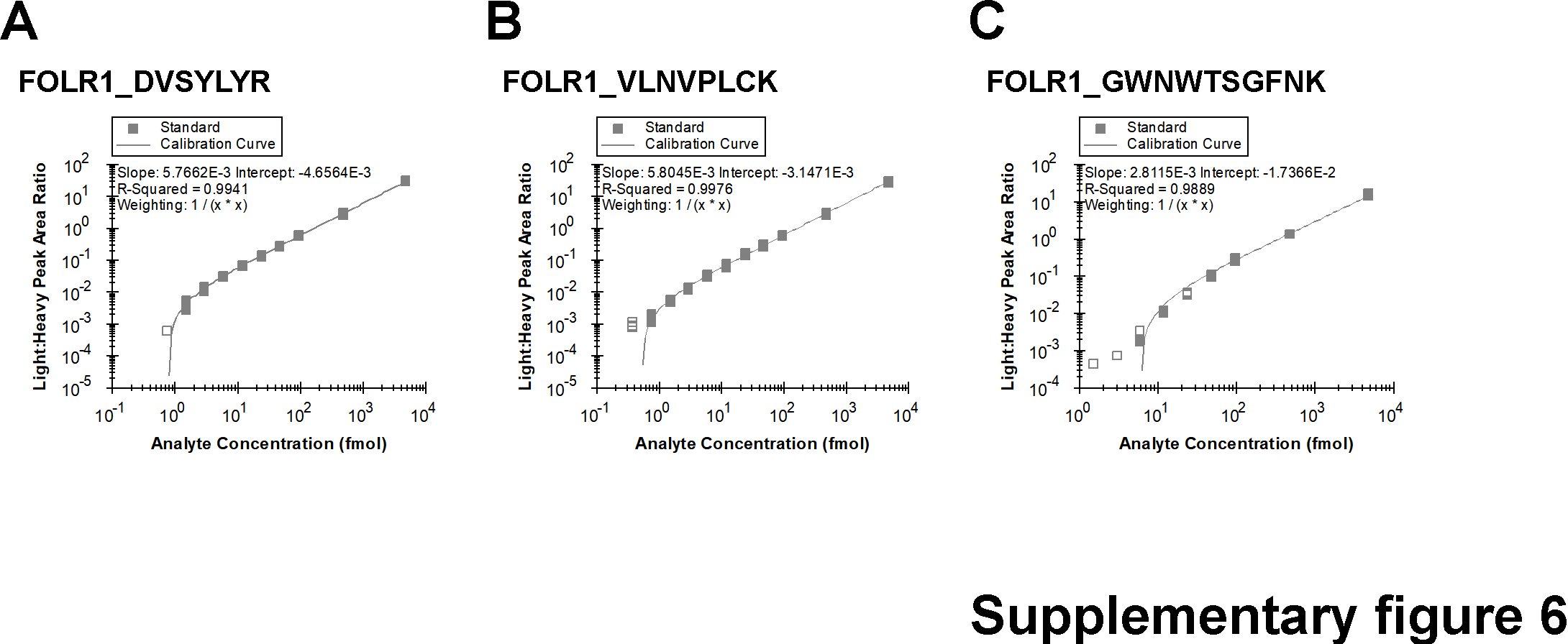

Supplement: Supplementary file 7 — Additional file 7: Figure S6. Calibration curves of peptides corresponding to FOLR1 protein. Gray square indicates each standard point. White square indicates standard points that were excluded from calibration curve with the accuracy of > 20%. Black line indicates the calibration curves. [file 12014_2023_9447_MOESM7_ESM.tif]

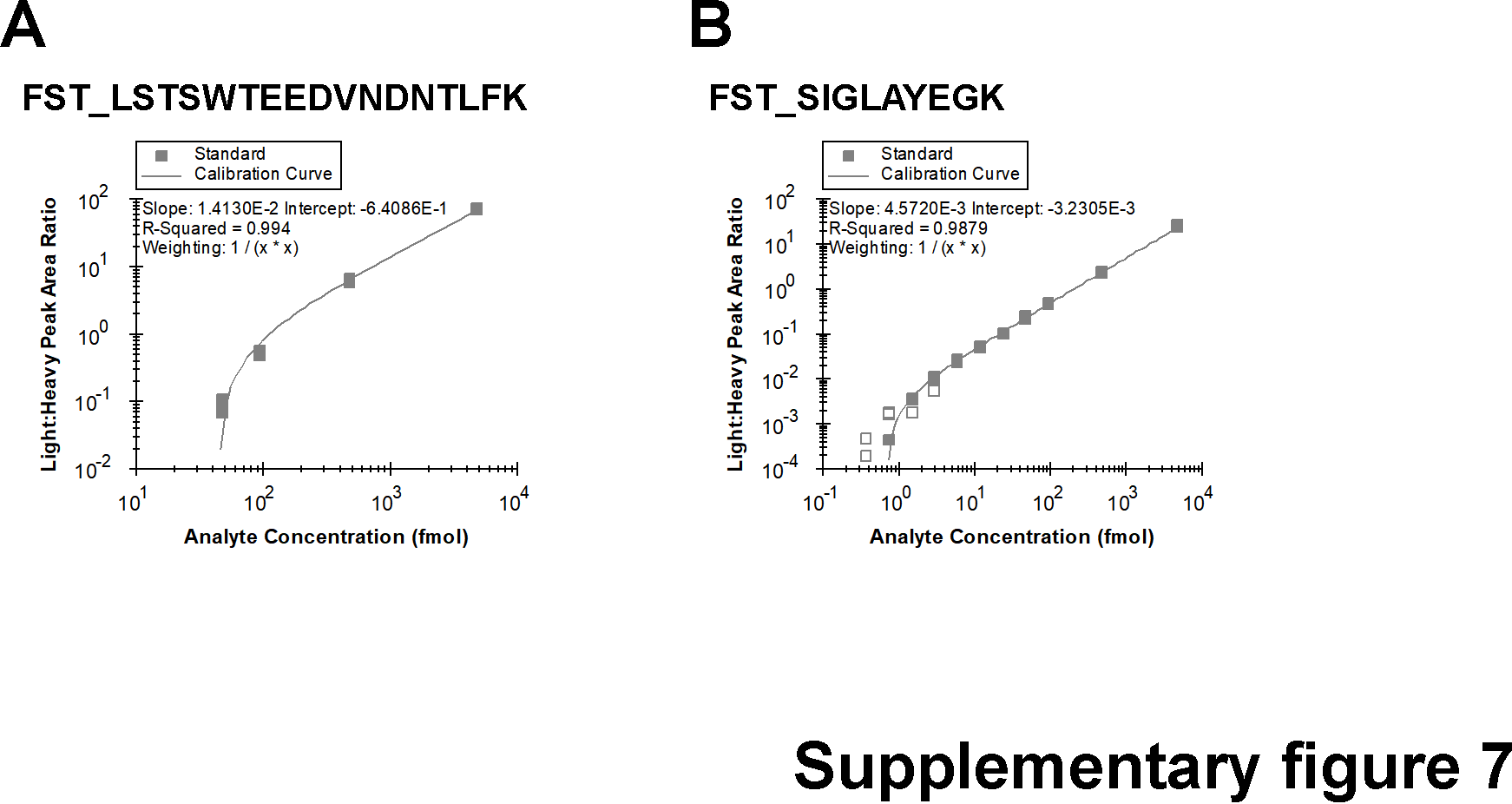

Supplement: Supplementary file 8 — Additional file 8: Figure S7. Calibration curves of peptides corresponding to FST protein. Gray square indicates each standard point. White square indicates standard points that were excluded from calibration curve with the accuracy of > 20%. Black line indicates the calibration curves. [file 12014_2023_9447_MOESM8_ESM.tif]

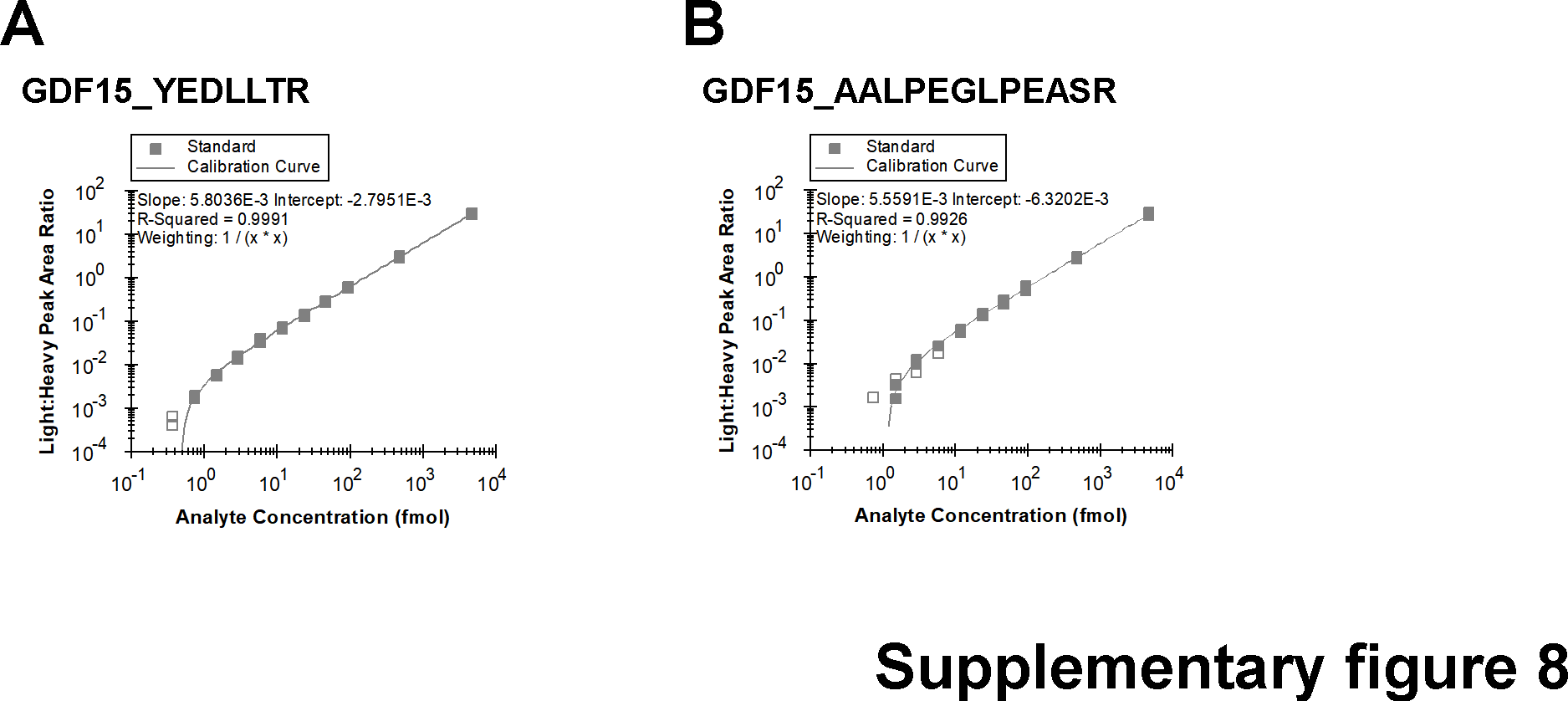

Supplement: Supplementary file 9 — Additional file 9: Figure S8. Calibration curves of peptides corresponding to GDF15 protein. Gray square indicates each standard point. White square indicates standard points that were excluded from calibration curve with the accuracy of > 20%. Black line indicates the calibration curves. [file 12014_2023_9447_MOESM9_ESM.tif]

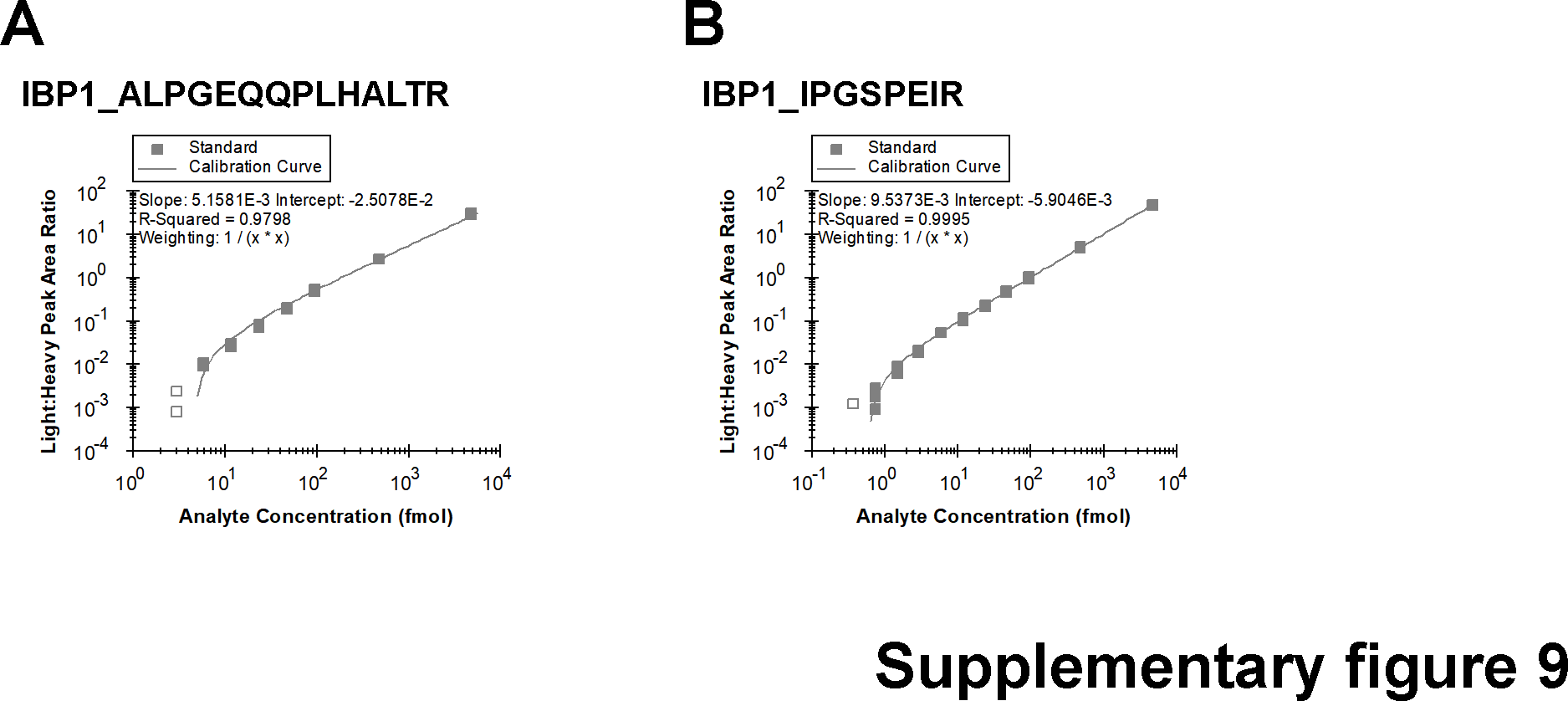

Supplement: Supplementary file 10 — Additional file 10: Figure S9. Calibration curves of peptides corresponding to IBP1 protein. Gray square indicates each standard point. White square indicates standard points that were excluded from calibration curve with the accuracy of > 20%. Black line indicates the calibration curves. [file 12014_2023_9447_MOESM10_ESM.tif]

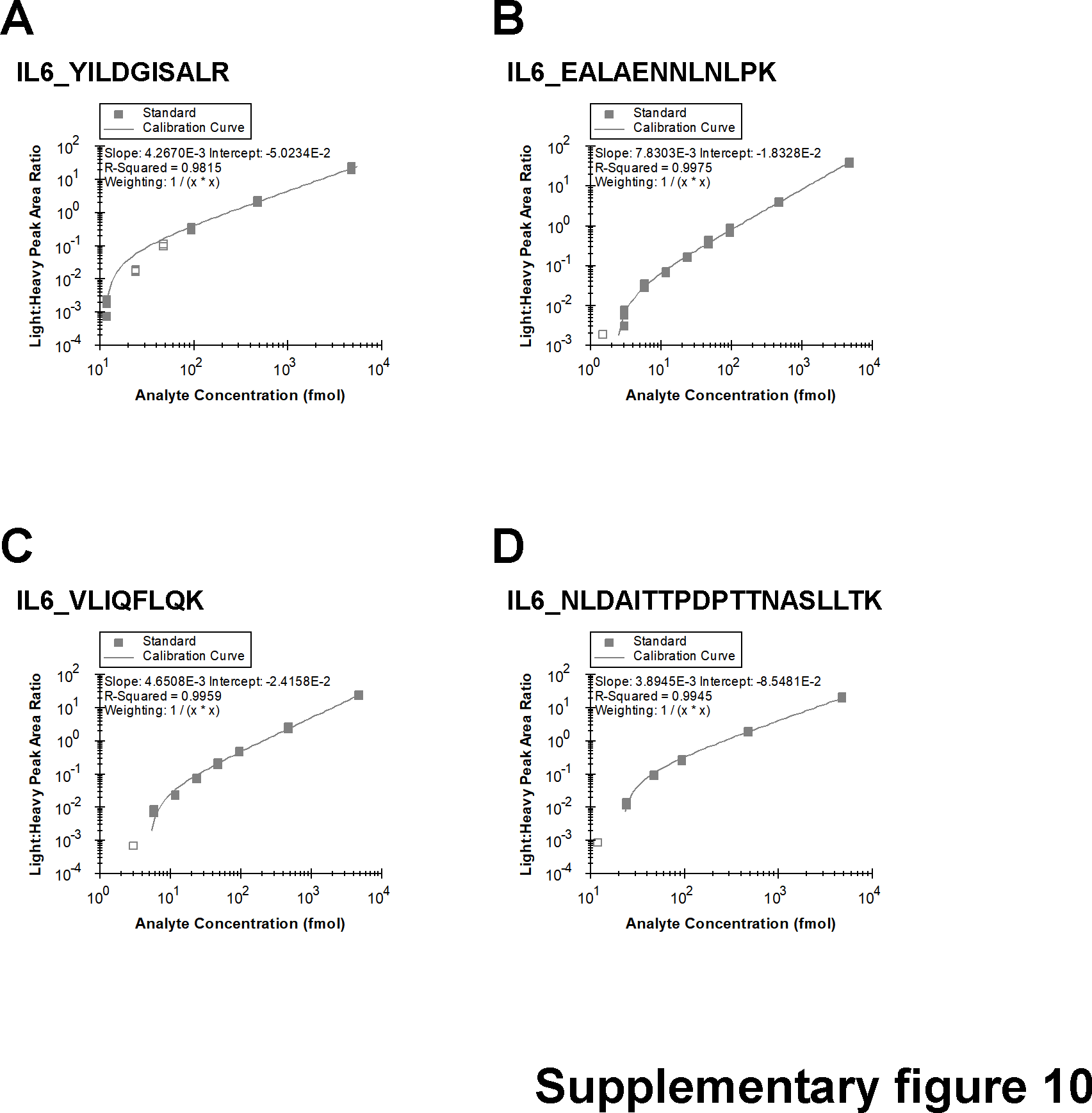

Supplement: Supplementary file 11 — Additional file 11: Figure S10. Calibration curves of peptides corresponding to IL6 protein. Gray square indicates each standard point. White square indicates standard points that were excluded from calibration curve with the accuracy of > 20%. Black line indicates the calibration curves. [file 12014_2023_9447_MOESM11_ESM.tif]

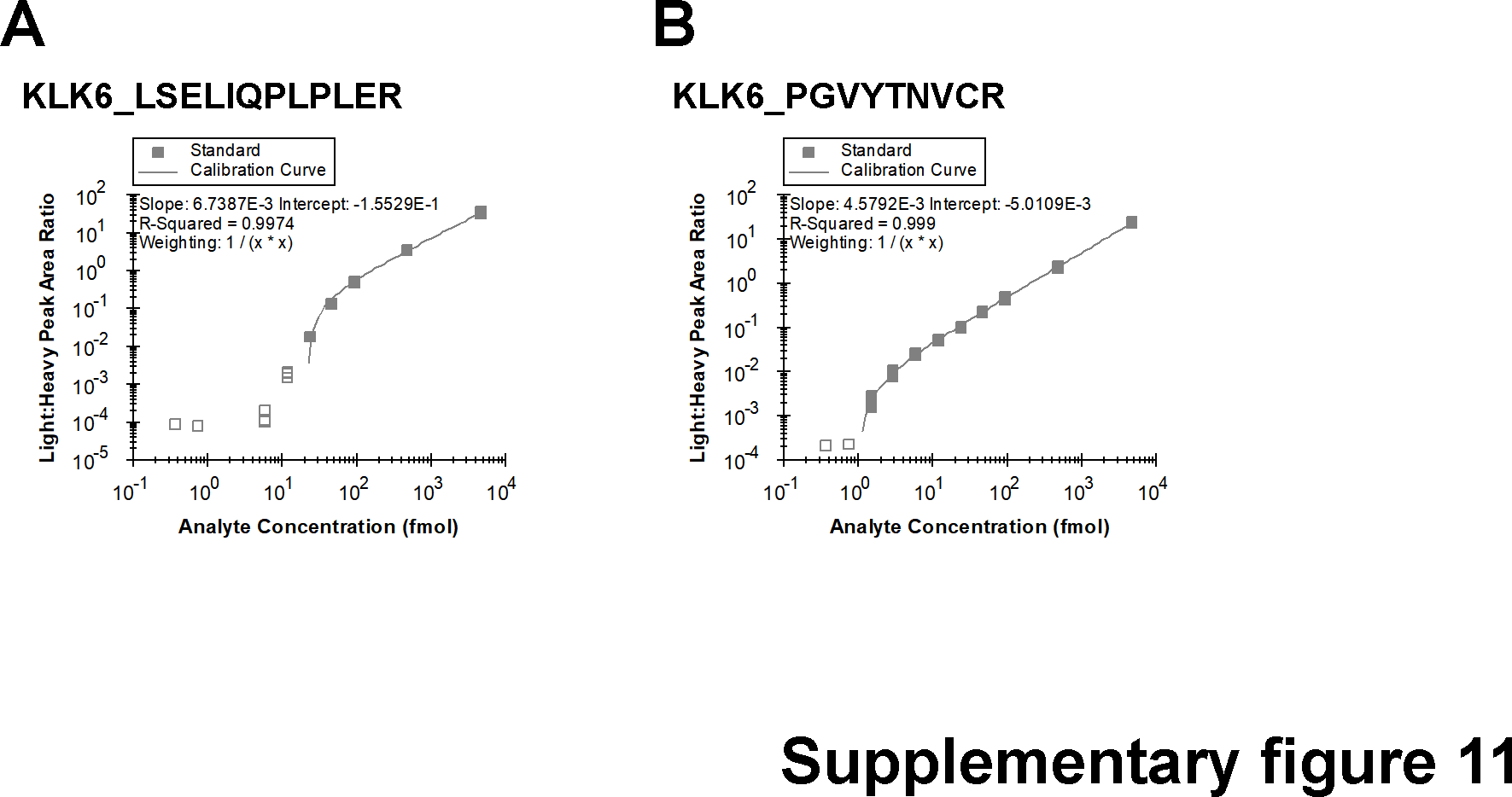

Supplement: Supplementary file 12 — Additional file 12: Figure S11. Calibration curves of peptides corresponding to KLK6 protein. Gray square indicates each standard point. White square indicates standard points that were excluded from calibration curve with the accuracy of > 20%. Black line indicates the calibration curves. [file 12014_2023_9447_MOESM12_ESM.tif]

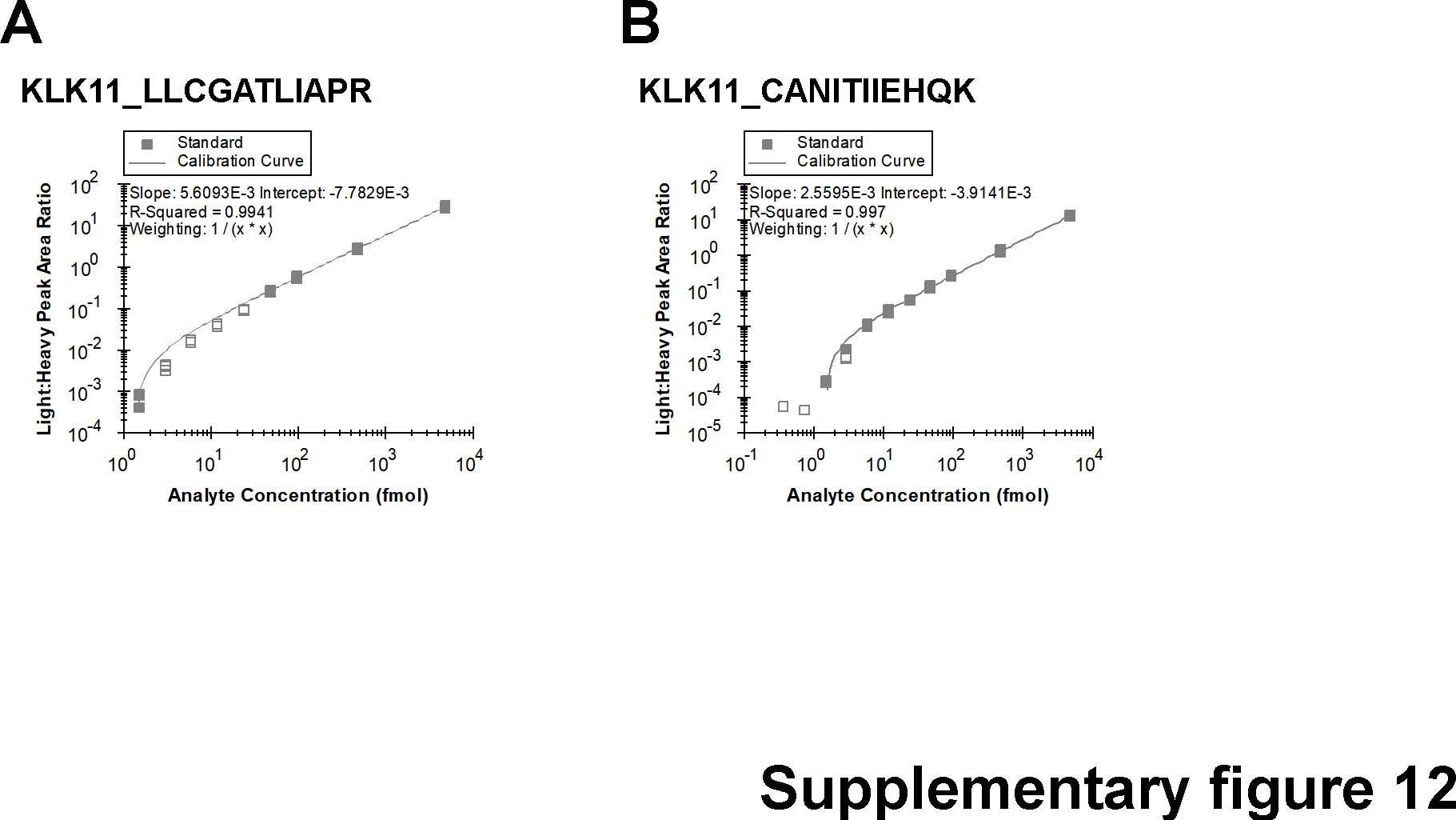

Supplement: Supplementary file 13 — Additional file 13: Figure S12. Calibration curves of peptides corresponding to KLK11 protein. Gray square indicates each standard point. White square indicates standard points that were excluded from calibration curve with the accuracy of > 20%. Black line indicates the calibration curves. [file 12014_2023_9447_MOESM13_ESM.tif]

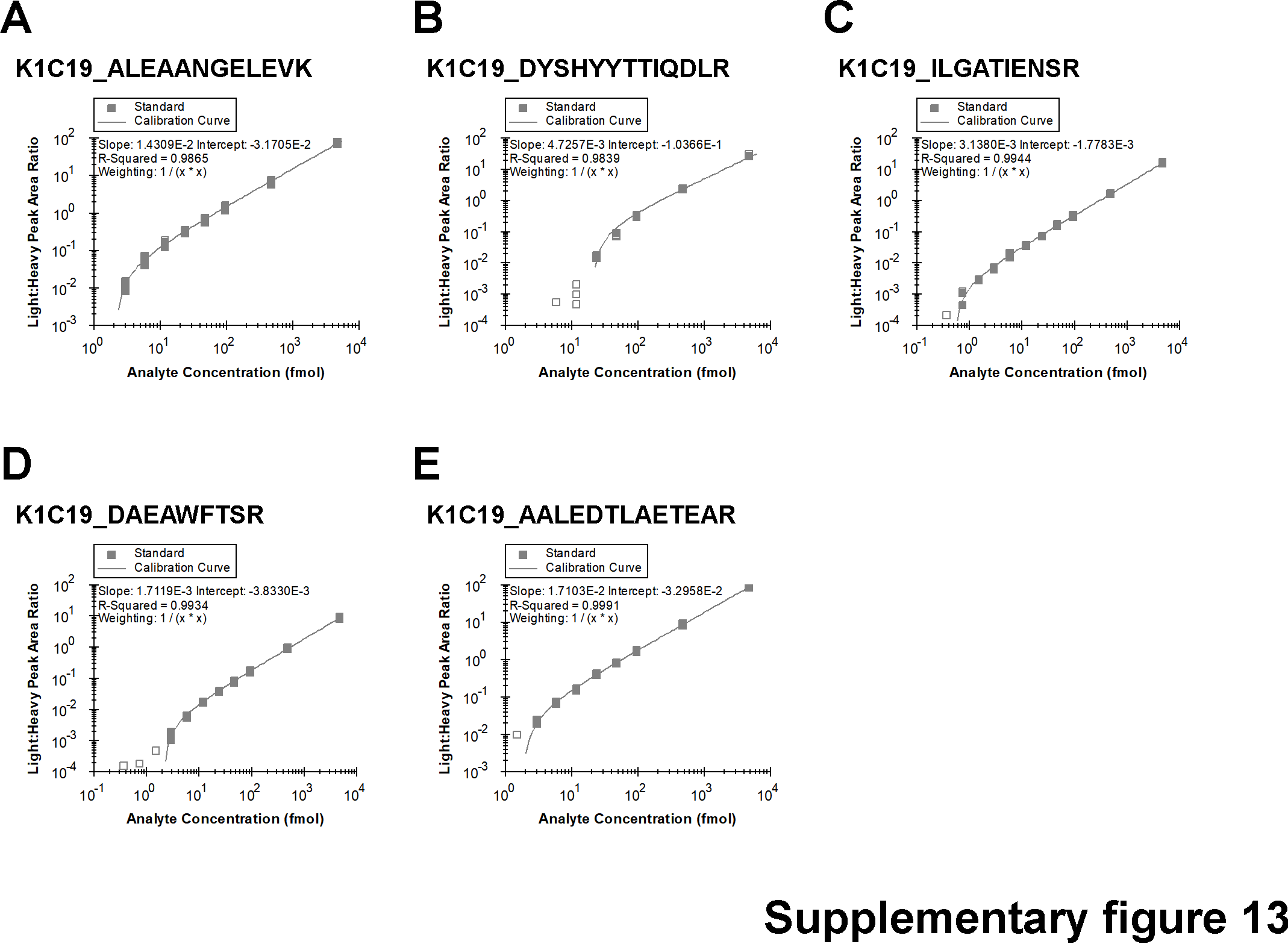

Supplement: Supplementary file 14 — Additional file 14: Figure S13. Calibration curves of peptides corresponding to K1C19 protein. Gray square indicates each standard point. White square indicates standard points that were excluded from calibration curve with the accuracy of > 20%. Black line indicates the calibration curves. [file 12014_2023_9447_MOESM14_ESM.tif]

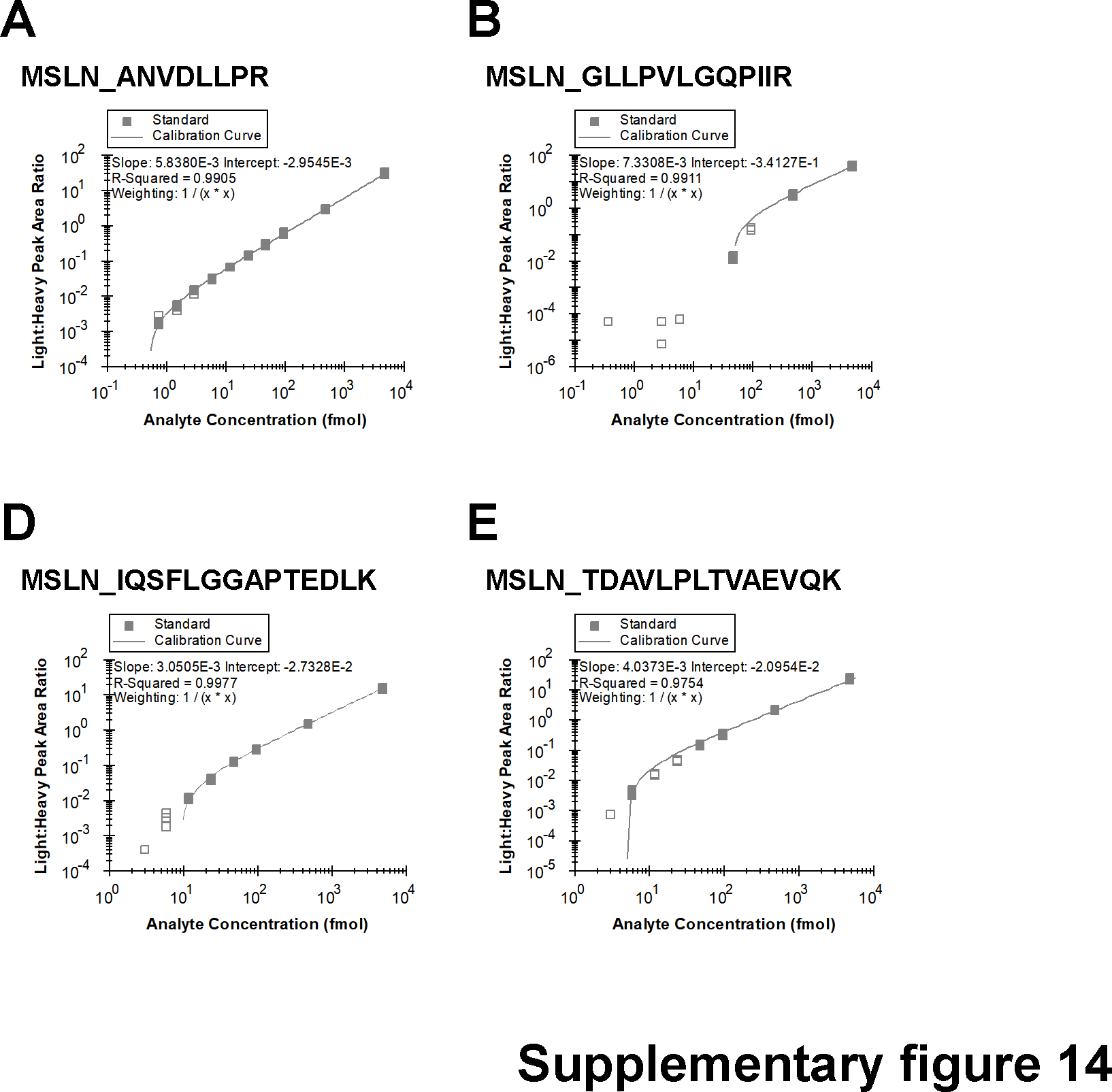

Supplement: Supplementary file 15 — Additional file 15: Figure S14. Calibration curves of peptides corresponding to MSLN protein. Gray square indicates each standard point. White square indicates standard points that were excluded from calibration curve with the accuracy of > 20%. Black line indicates the calibration curves. [file 12014_2023_9447_MOESM15_ESM.tif]

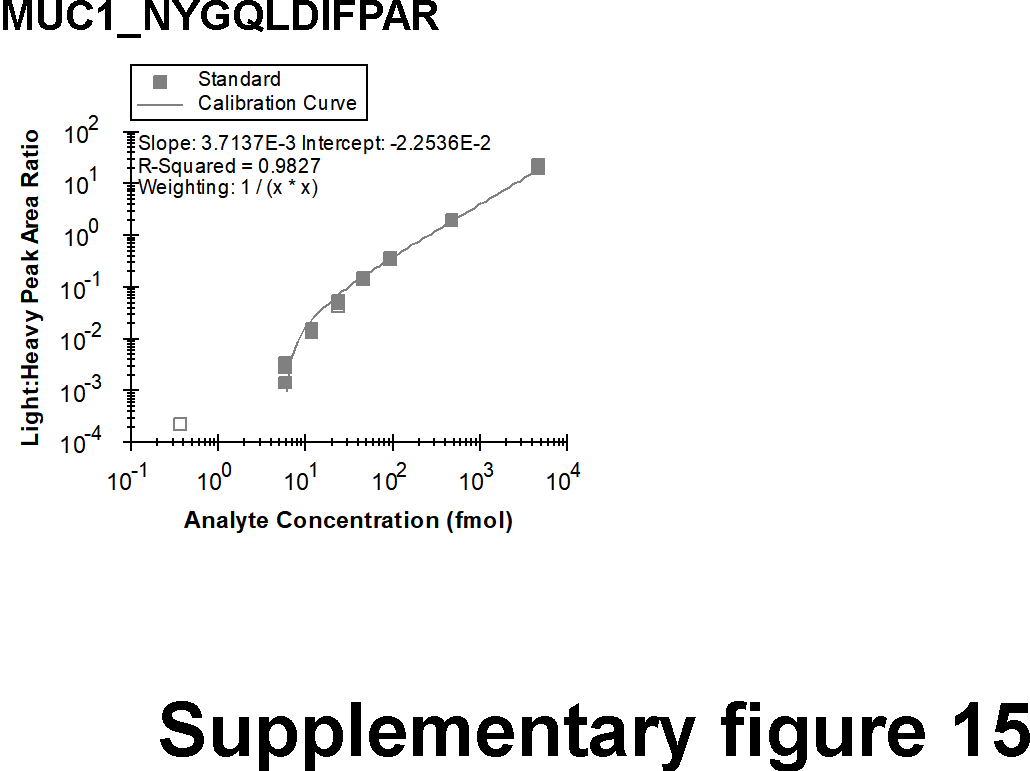

Supplement: Supplementary file 16 — Additional file 16: Figure S15. Calibration curves of a peptide corresponding to MUC1 protein. Gray square indicates each standard point. White square indicates standard points that were excluded from calibration curve with the accuracy of > 20%. Black line indicates the calibration curves. [file 12014_2023_9447_MOESM16_ESM.tif]

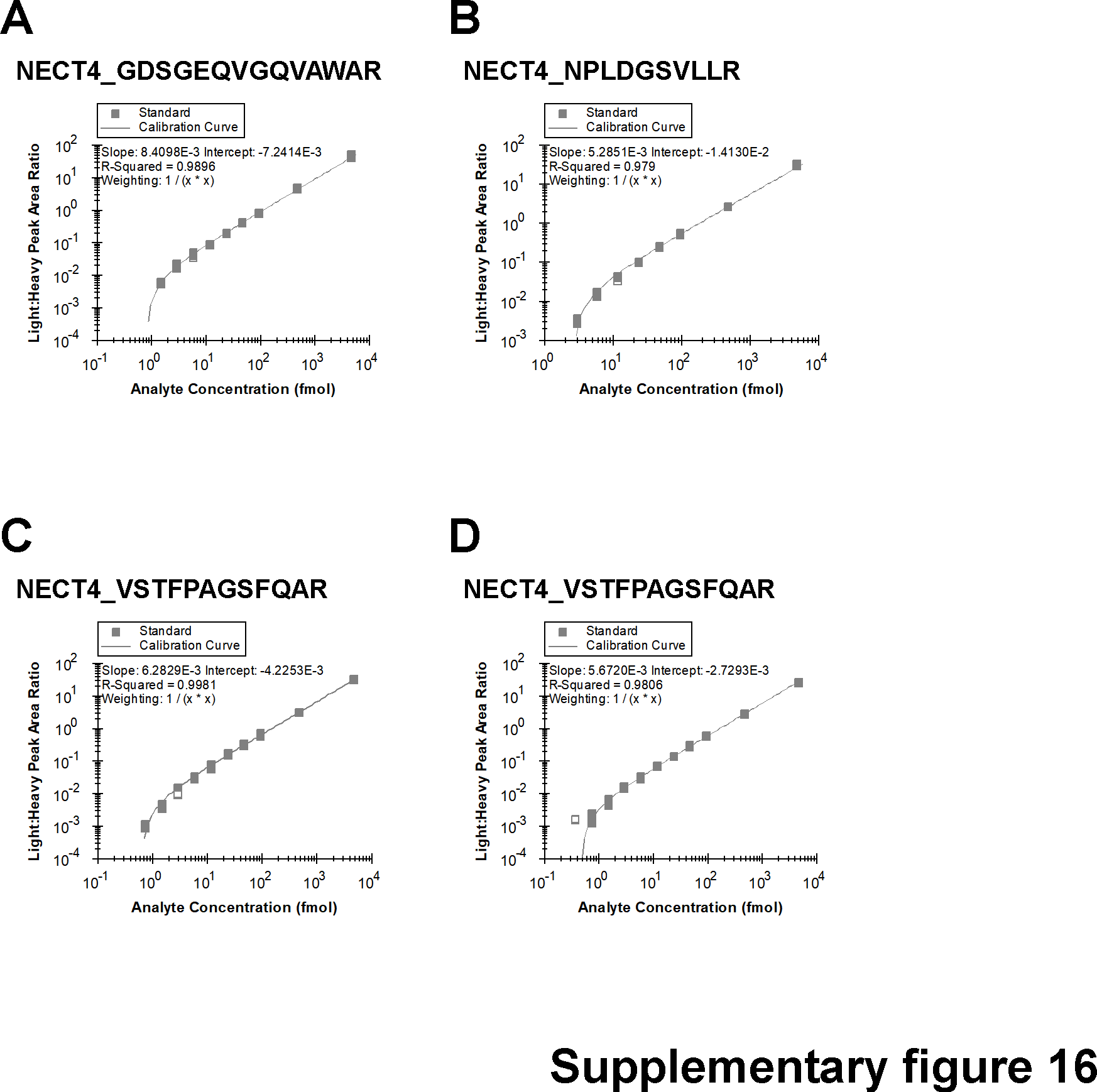

Supplement: Supplementary file 17 — Additional file 17: Figure S16. Calibration curves of peptides corresponding to NECT4 protein. Gray square indicates each standard point. White square indicates standard points that were excluded from calibration curve with the accuracy of > 20%. Black line indicates the calibration curves. [file 12014_2023_9447_MOESM17_ESM.tif]

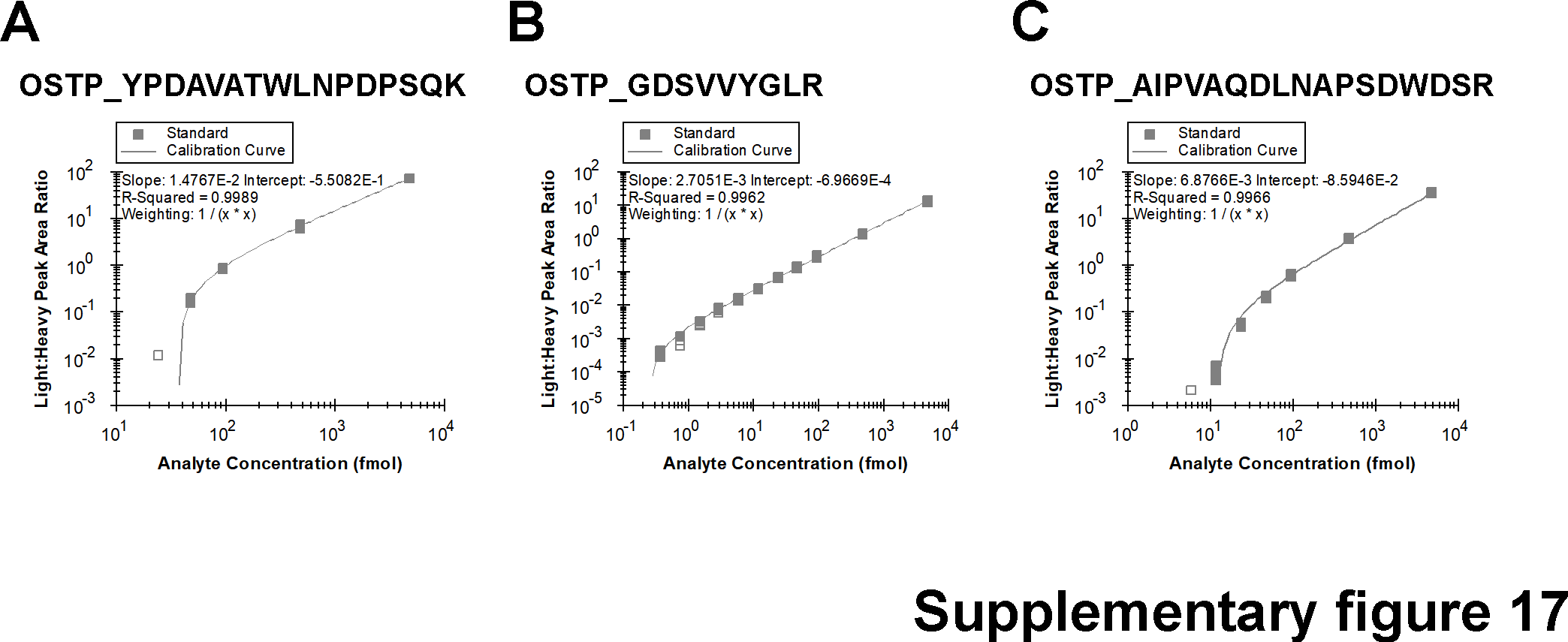

Supplement: Supplementary file 18 — Additional file 18: Figure S17. Calibration curves of peptides corresponding to OSTP protein. Gray square indicates each standard point. White square indicates standard points that were excluded from calibration curve with the accuracy of > 20%. Black line indicates the calibration curves. [file 12014_2023_9447_MOESM18_ESM.tif]

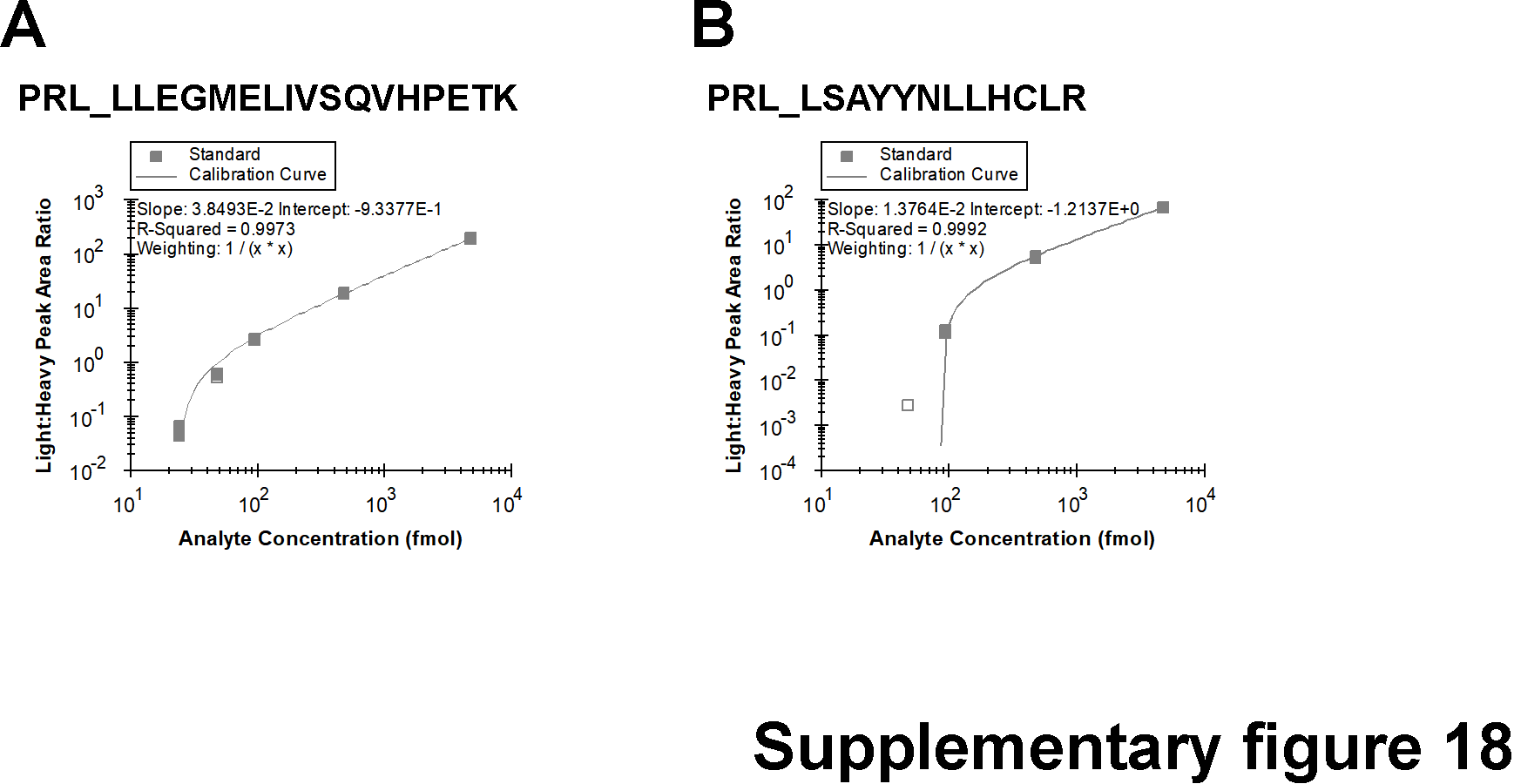

Supplement: Supplementary file 19 — Additional file 19: Figure S18. Calibration curves of peptides corresponding to PRL protein. Gray square indicates each standard point. White square indicates standard points that were excluded from calibration curve with the accuracy of > 20%. Black line indicates the calibration curves. [file 12014_2023_9447_MOESM19_ESM.tif]

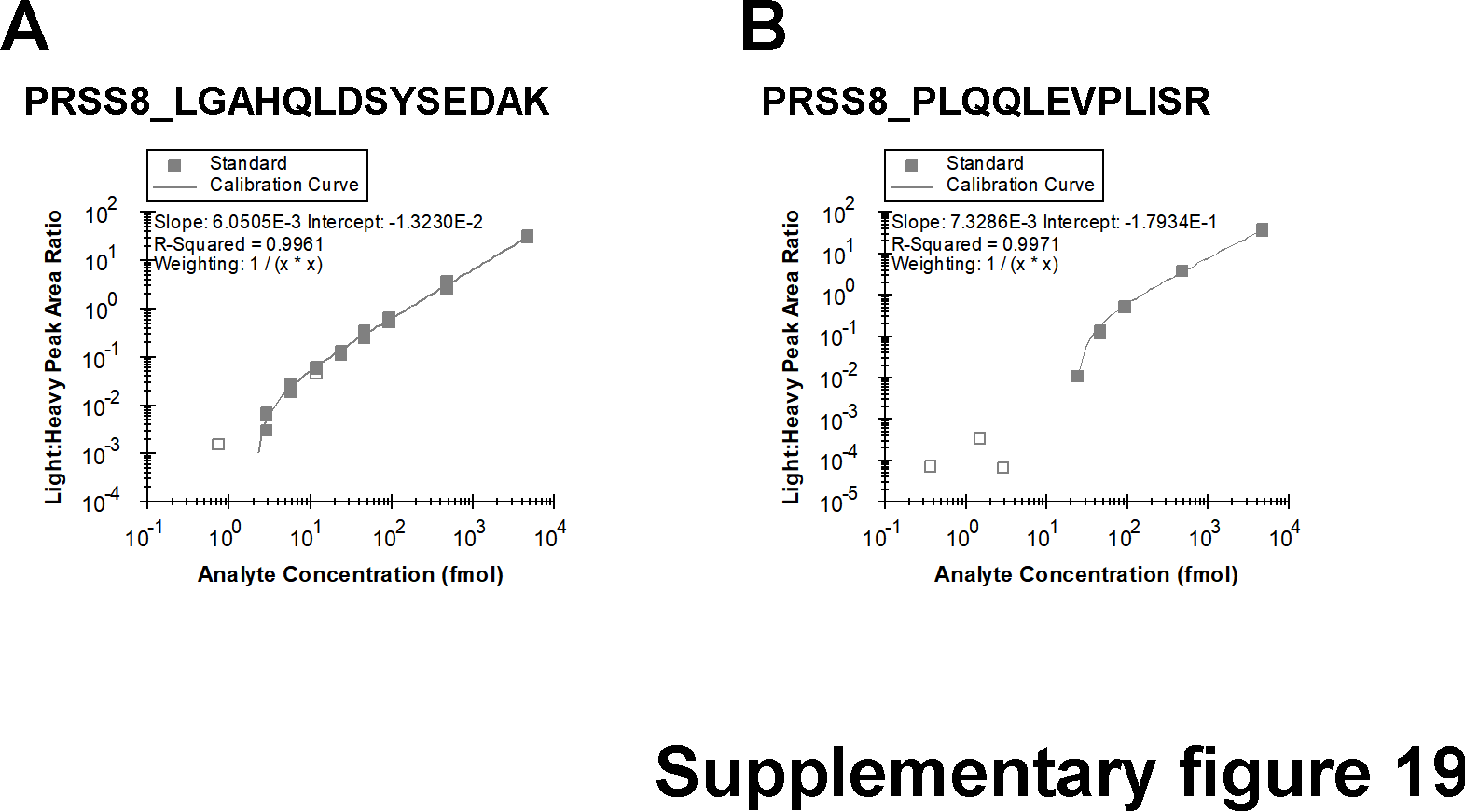

Supplement: Supplementary file 20 — Additional file 20: Figure S19. Calibration curves of peptides corresponding to PRSS8 protein. Gray square indicates each standard point. White square indicates standard points that were excluded from calibration curve with the accuracy of > 20%. Black line indicates the calibration curves. [file 12014_2023_9447_MOESM20_ESM.tif]

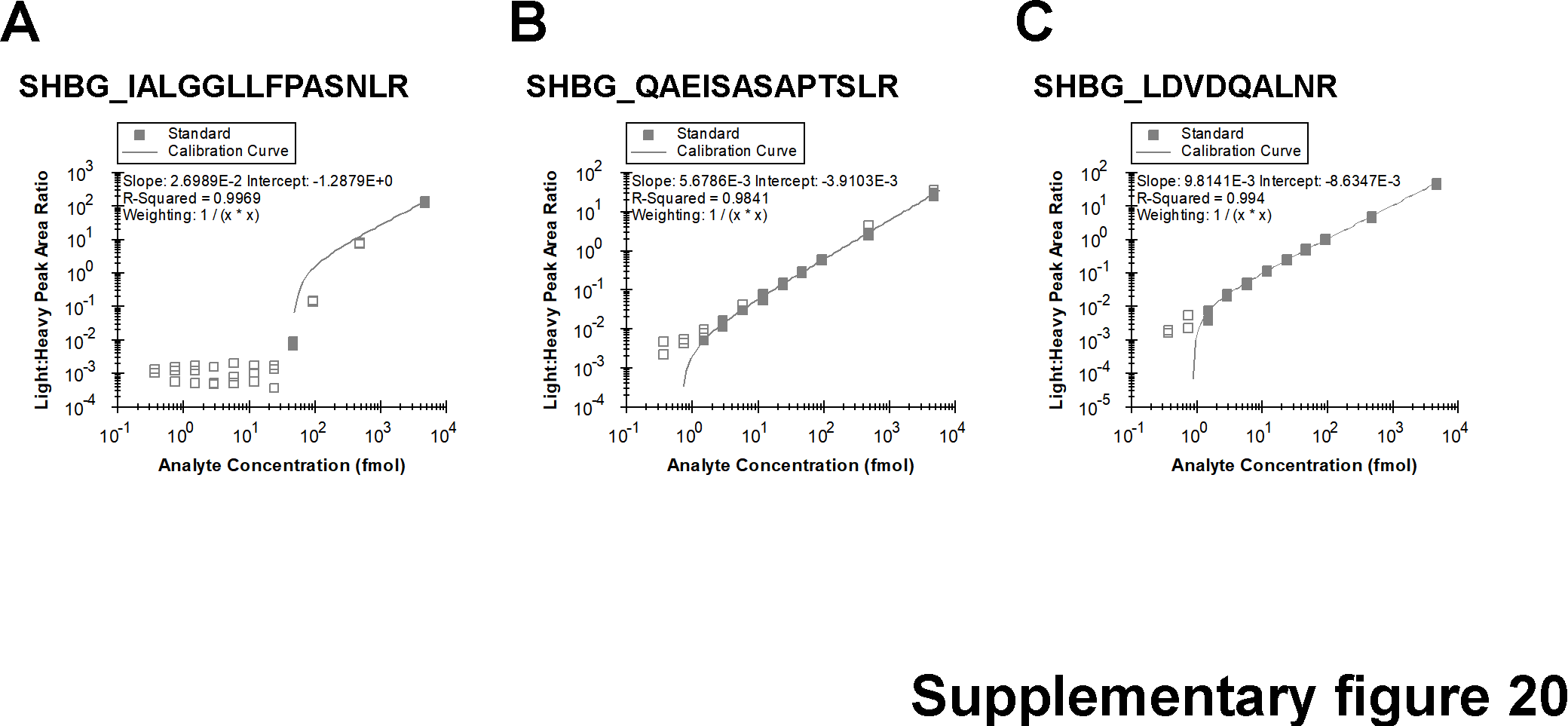

Supplement: Supplementary file 21 — Additional file 21: Figure S20. Calibration curves of peptides corresponding to SHBG protein. Gray square indicates each standard point. White square indicates standard points that were excluded from calibration curve with the accuracy of > 20%. Black line indicates the calibration curves. [file 12014_2023_9447_MOESM21_ESM.tif]

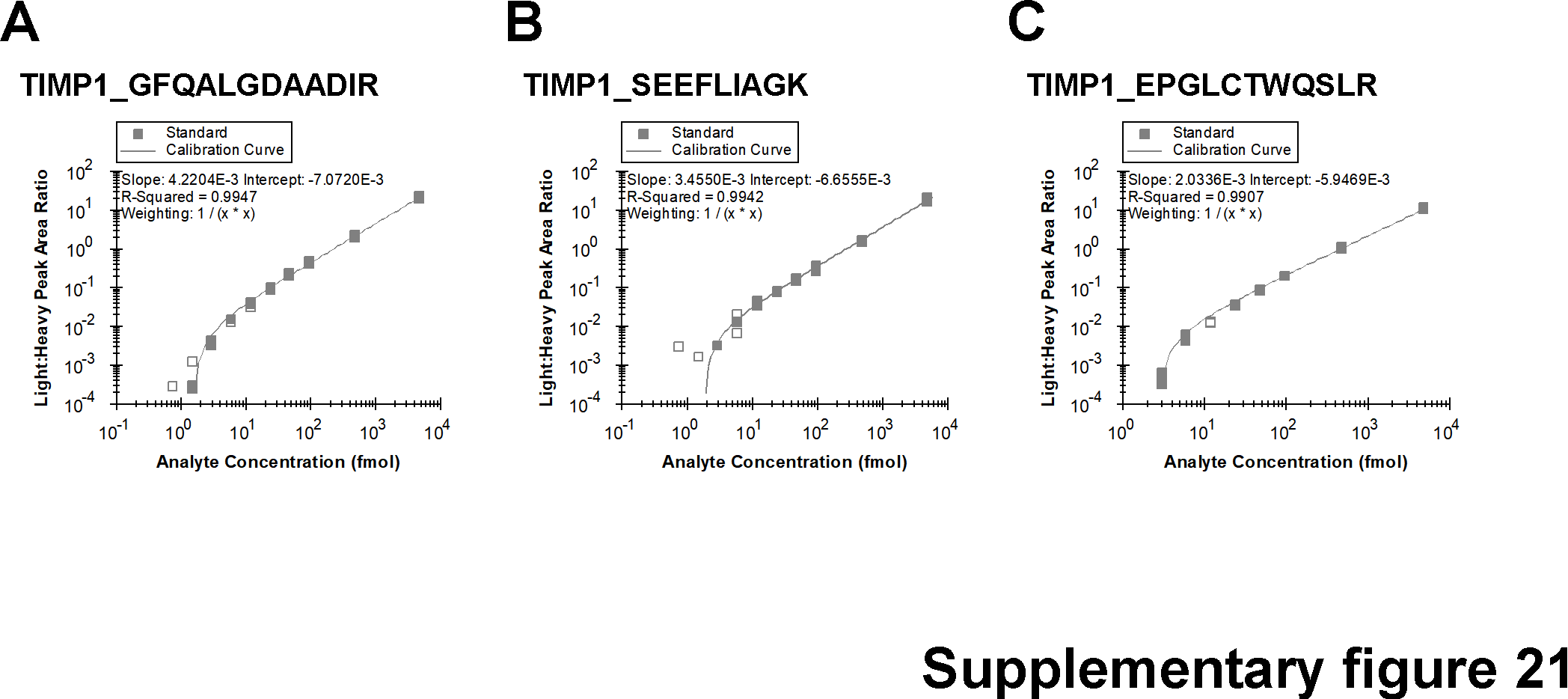

Supplement: Supplementary file 22 — Additional file 22: Figure S21. Calibration curves of peptides corresponding to TIMP1 protein. Gray square indicates each standard point. White square indicates standard points that were excluded from calibration curve with the accuracy of > 20%. Black line indicates the calibration curves. [file 12014_2023_9447_MOESM22_ESM.tif]

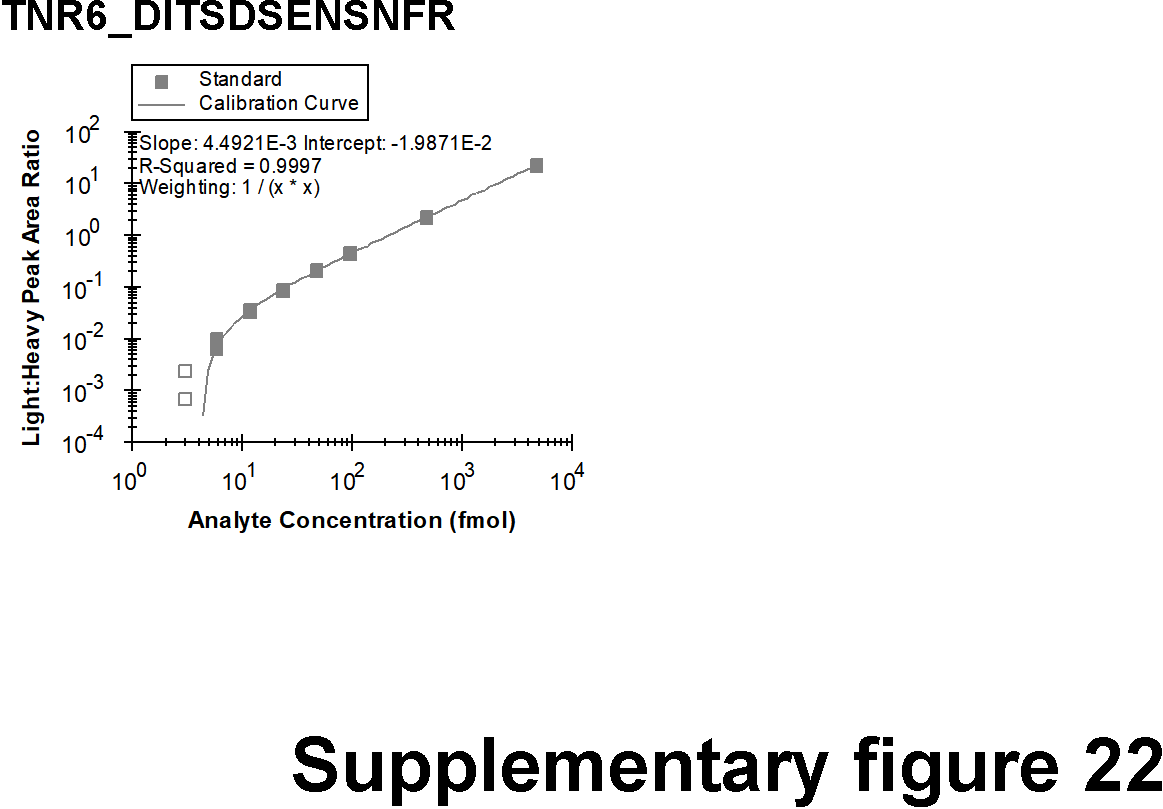

Supplement: Supplementary file 23 — Additional file 23: Figure S22. Calibration curves of a peptide corresponding to TNR6 protein. Gray square indicates each standard point. White square indicates standard points that were excluded from calibration curve with the accuracy of > 20%. Black line indicates the calibration curves. [file 12014_2023_9447_MOESM23_ESM.tif]

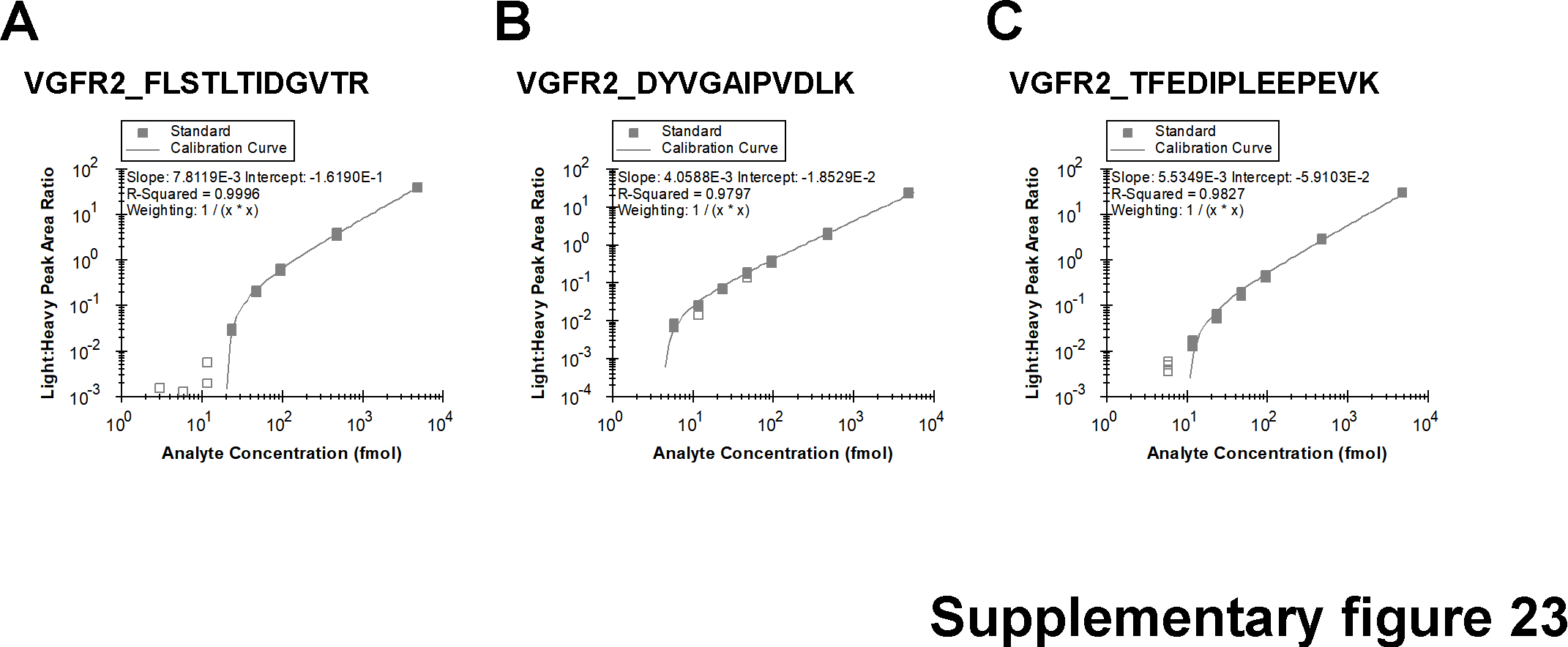

Supplement: Supplementary file 24 — Additional file 24: Figure S23. Calibration curves of peptides corresponding to VGFR2 protein. Gray square indicates each standard point. White square indicates standard points that were excluded from calibration curve with the accuracy of > 20%. Black line indicates the calibration curves. [file 12014_2023_9447_MOESM24_ESM.tif]

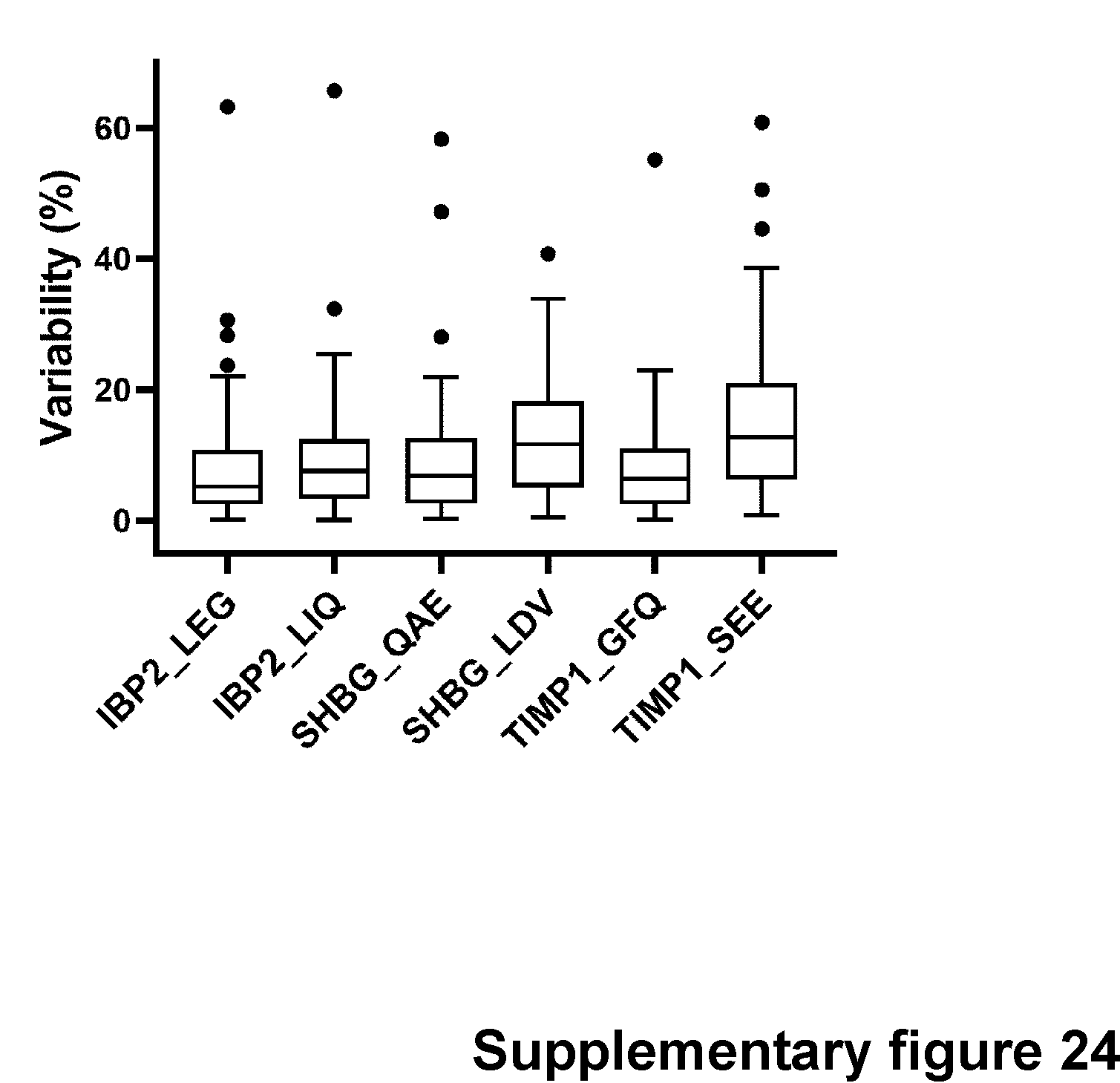

Supplement: Supplementary file 25 — Additional file 25: Figure S24. Evaluation of precision of quantified peptides. Variability for each peptide from all 69 serum samples including non-cancerous (n=18), benign ovarian condition (n=18), early-stage serous ovarian (n=16), and late-stage serous ovarian (n=17) patients. [file 12014_2023_9447_MOESM25_ESM.tif]

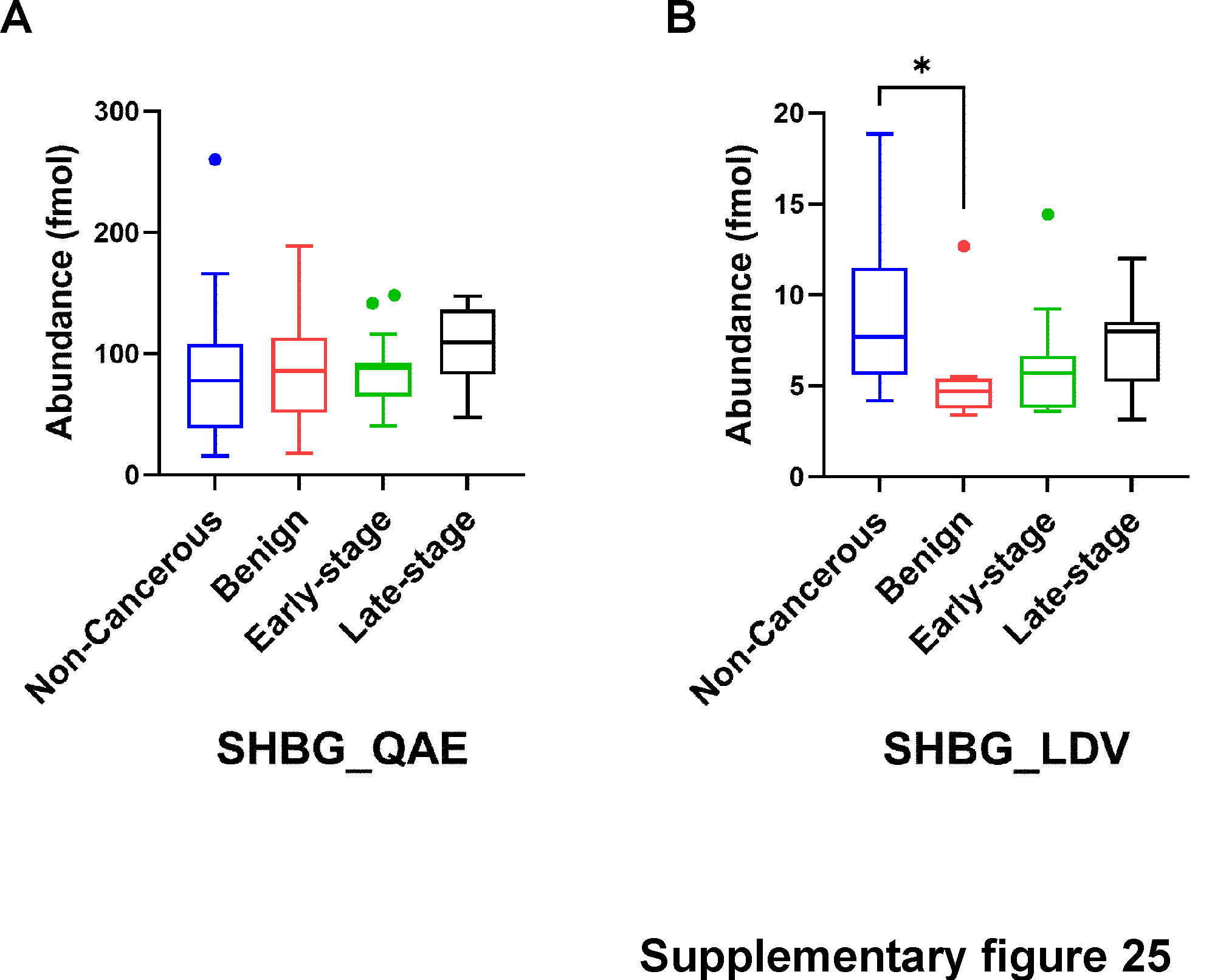

Supplement: Supplementary file 26 — Additional file 26: Figure S25. Statistical analysis of SHBG_QAE and SHBG_LDV corresponding to SHBG protein. A SHBG_QAE peptide. B SHBG_LDV peptide. A post-hoc test was used for pairwise comparison of the abundance of peptides from non-cancerous, benign ovarian condition (benign), early-stage serous ovarian cancer (early-stage), and late-stage serous ovarian cancer (late-stage) sera. The abundance indicates the on-column concentration based on an injection volume of 10 µL. * indicates p < 0.05. [file 12014_2023_9447_MOESM26_ESM.tif]

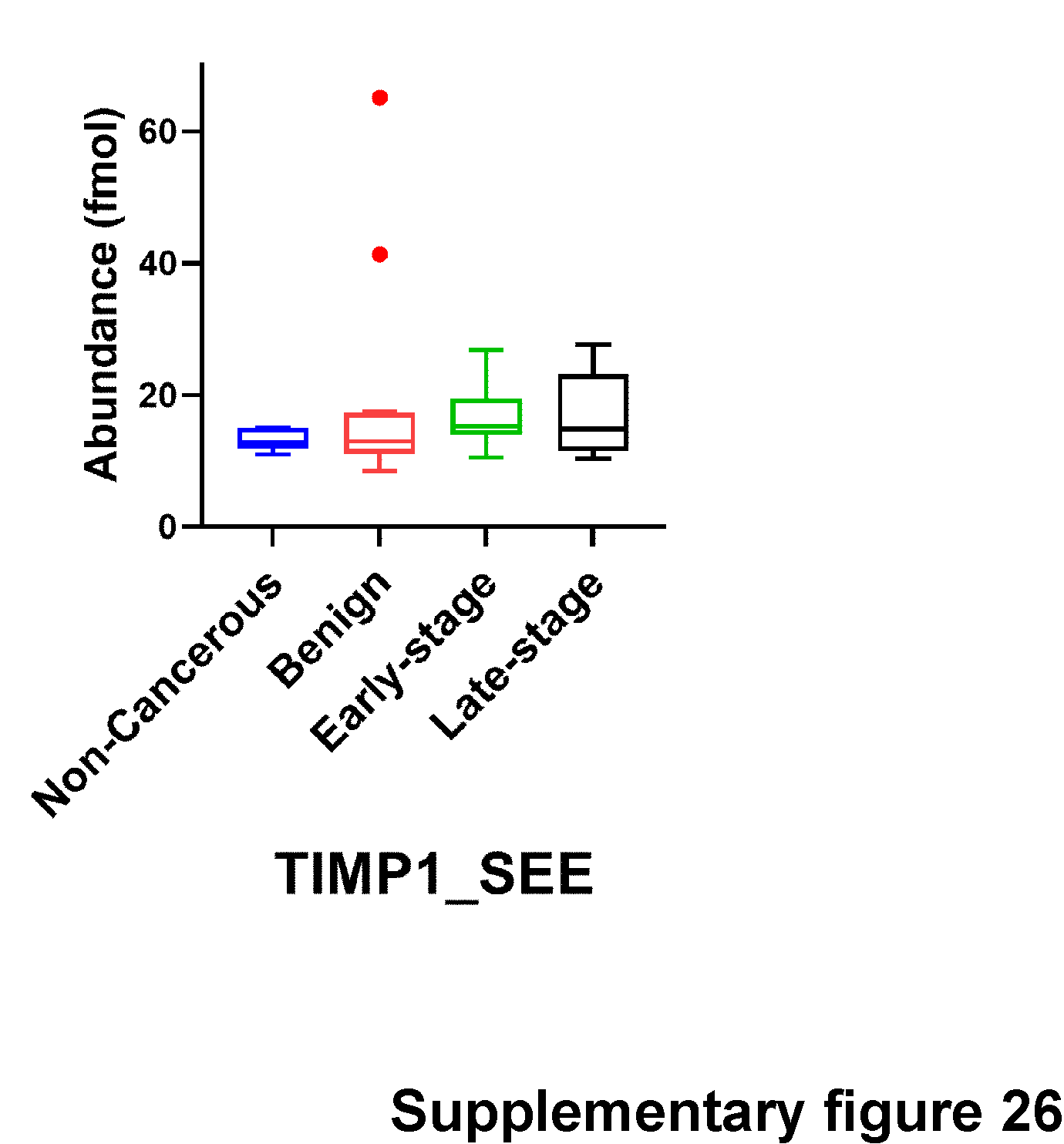

Supplement: Supplementary file 27 — Additional file 27: Figure S26. Statistical analysis of TIMP1_SEE corresponding to TIMP1 protein. The abundance indicates the on-column concentration based on an injection volume of 10 µL. [file 12014_2023_9447_MOESM27_ESM.tif]
